# Pyrrole–Aminopyrimidine Ensembles: Cycloaddition of Guanidine to Acylethynylpyrroles

**DOI:** 10.3390/molecules26061692

**Published:** 2021-03-17

**Authors:** Olga V. Petrova, Arsalan B. Budaev, Elena F. Sagitova, Igor A. Ushakov, Lyubov N. Sobenina, Andrey V. Ivanov, Boris A. Trofimov

**Affiliations:** A.E. Favorsky Irkutsk Institute of Chemistry, Siberian Branch, Russian Academy of Sciences, 1 Favorsky Street, 664033 Irkutsk, Russia; ol_petr@irioch.irk.ru (O.V.P.); arsalan@irioch.irk.ru (A.B.B.); sagitova@irioch.irk.ru (E.F.S.); igor-papa71@mail.ru (I.A.U.); sobenina@irioch.irk.ru (L.N.S.); ivanov@irioch.irk.ru (A.V.I.)

**Keywords:** acylethynylpyrroles, guanidine, cyclocondensation, aminopyrimidine

## Abstract

An efficient method for the synthesis of pharmaceutically prospective pyrrole–aminopyrimidine ensembles (in up to 91% yield) by the cyclocondensation of easily available acylethynylpyrroles with guanidine nitrate has been developed. The reaction proceeds under heating (110–115 °C, 4 h) in the KOH/DMSO system. In the case of 2-benzoylethynylpyrrole, the unexpected addition of the formed pyrrole–aminopyrimidine as N- (NH moiety of the pyrrole ring) and C- (CH of aminopyrimidine) nucleophiles to the triple bond is observed.

## 1. Introduction

One of the main trends in modern organic chemistry is the synthesis of heterocyclic ensembles, each of the fragments of which has promising biological activity. These ensembles include, in particular, pyrrole–pyrimidines, which combine two of the most fundamental life-supporting molecular systems in their molecule and represent privileged objects for drug design.

The pyrrole core is a key structural motif in a plethora of natural products such as chlorophyll, hemoglobin, bile pigments, vitamin B_12_, and others [1]. Pyrrole and its derivatives are also important components of a number of pharmaceuticals and new compounds with a variety of pharmacological activity [2,3]. Basing on pyrroles, anti-tumor agent sunitinib, the anti-hyperlipidemic atorvastatin [2,3], neotropic aloracetam [2], antipsychotic elopiprazole [2], and nonsteroidal anti-inflammatory agent tolmetin [2] were created.

The pyrimidine ring is a main structural moiety of nucleic acids, vitamins, coenzymes, and uric acid [4], as well as the frequent scaffold in medicines [4,5]. According to the literature data, the presence of the amino group in pyrimidine enhances its pharmacological properties [5]. The aminopyrimidine ring is a fragment of nucleotide bases in DNA and RNA, which are important components of living cells [4]. Substituted 2-aminopyrimidines exhibit cardiotonic [6], antitrypanosomal and antiplasmodial [7], antimicrobial [8,9,10,11] and antiplatelet aggregation activity [12]. They are also ligands of histamine H_4_ and H_3_ receptors [13,14], inhibitors of IRAK4 (interleukin-1 receptor-associated kinase 4) [15], the vascular endothelial growth factor inhibitor [16], serine/threonine protein kinase inhibitors, candidates for treating drug-resistant tuberculosis [17]. There are numerous 2-aminopyrimidine-tailored pharmaceuticals [18,19] including antiviral (Lamivudine [18], Etravirine and Rilpivirine [19]), anti-cancer (Imatinib [18,19], Erlotinib, Lapatinib [18], Nilotinib, Dabrafenib, Ceritinib, Osimertinib, and Pazopanib [19]), anxiolytic (Buspirone) [19], hypolipidemic (Rosuvastatin) [18], etc.

2-Aminopyrimidines with pyrrole substituents represent molecular systems, which could be particularly promising for medicinal chemistry, due to the presence of two pharmacologically active units in their structure. This assumption is supported by the fact that among pyrrole–aminopyrimidines there are inhibitors of JAK2 (Janus kinase 2) [20,21], Cdc7 kinase (Cell division cycle 7-related protein kinase) [22,23], and Polo-like kinase 1 [24], as well as representatives with prominent antifungal activity against *Aspergillus niger* [25].

The fact that pyrrole–aminopyrimidines have a great potential for cancer treatment [20,21,22,23,24] generates interest in the targeted synthesis of these derivatives.

Few known syntheses of pyrrole–aminopyrimidine ensembles are based on three approaches. The first is to build up the pyrrole moiety on aminopyrimidines starting from 1-(2-aminopyrimidin-4-yl)-2-bromoethanones [20,23,26].

The second approach involves construction of the aminopyrimidine ring via the addition of guanidine to pyrroles with ethylenic substituents. Among the latter are 3-dimethylamino-2-(pyrrole-2-carbonyl)acrylonitrile [27], benzylidene acetyl pyrrole [28], pyrrolylenaminone [20], pyrrolyl vinamidinium salts [29].

The third approach to the synthesis of pyrrole–aminopyrimidine ensembles is the coupling of halopyrimidines with pyrroles under Buchwald–Hartwig conditions [21] or their boronate derivative under Suzuki reaction conditions and PdCl_2_(dppf) catalysis [21]. (Pyrrol-2-yl)-2-aminopyrimidine was also obtained from *N*-Boc-pyrrole, which was deprotonated upon treatment with LiTMP (Lithium tetramethylpiperidide) and, after subsequent transmetalation (using ZnCl_2_·TMEDA) and coupling with 2,4-dichloropyrimidine, was transformed into *N*-Boc-2-(2-chloro-4-pyrimidyl)pyrrole [30]. The chloro group of the latter was substituted with allylamine, and after subsequent cleavage of the allyl group under classical conditions afforded the target product.

The most common approach to the synthesis of aminopyrimidines, i.e., cyclocondensation of alkynones with guanidine [31,32,33,34,35,36,37,38], was not always applied to the preparation of pyrrole–aminopyrimidines.

**Scheme 1:**
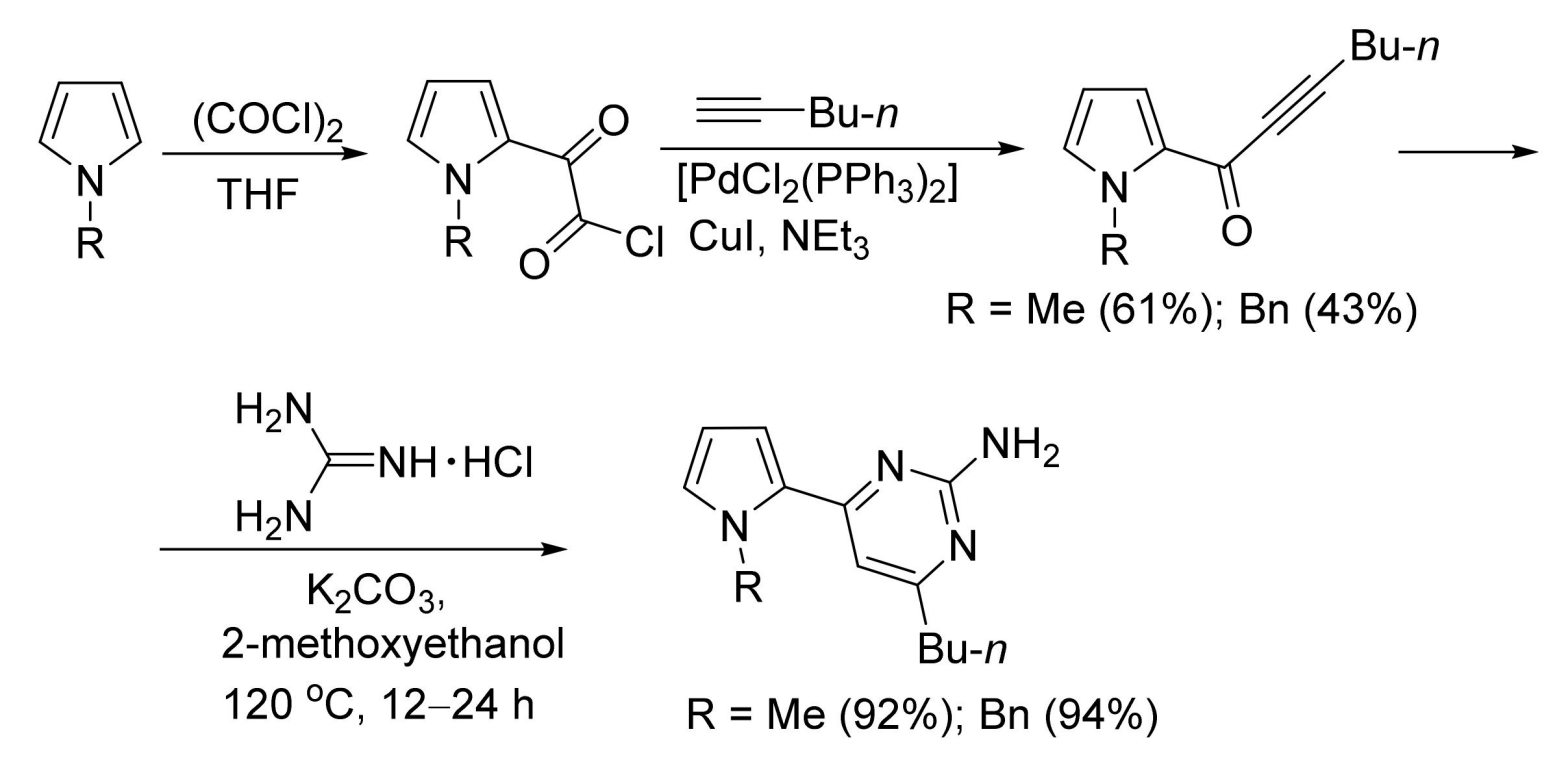
Synthesis of pyrrole–aminopyrimidines from pyrrol-2-ylhexynones and guanidine. Previous work [39].

To our knowledge, there is only one work concerning the synthesis of pyrrole–aminopyrimidines from guanidine and pyrrol-2-ylhexynones [39], obtained by glyoxylation of *N*-methyl- and *N*-benzylpyrroles with oxalyl chloride and subsequent Pd/Cu-catalyzed decarbonylative alkynylation of the pyrrolylglyoxylyl chlorides with hexyne (Scheme 1).

This is likely due to the fact that, until recently, alkynones with pyrrole substituents were difficult to obtain. Such compounds have become readily available owing to the discovery of the cross-coupling reaction of pyrroles with acylhaloacetylenes in the medium of solid oxides and metal salts [40,41,42], and was widely used by us in the synthesis of diverse pyrrole heterocyclic ensembles and fused heterocyclic systems [42].

## 2. Results and Discussion

**Scheme 2:**
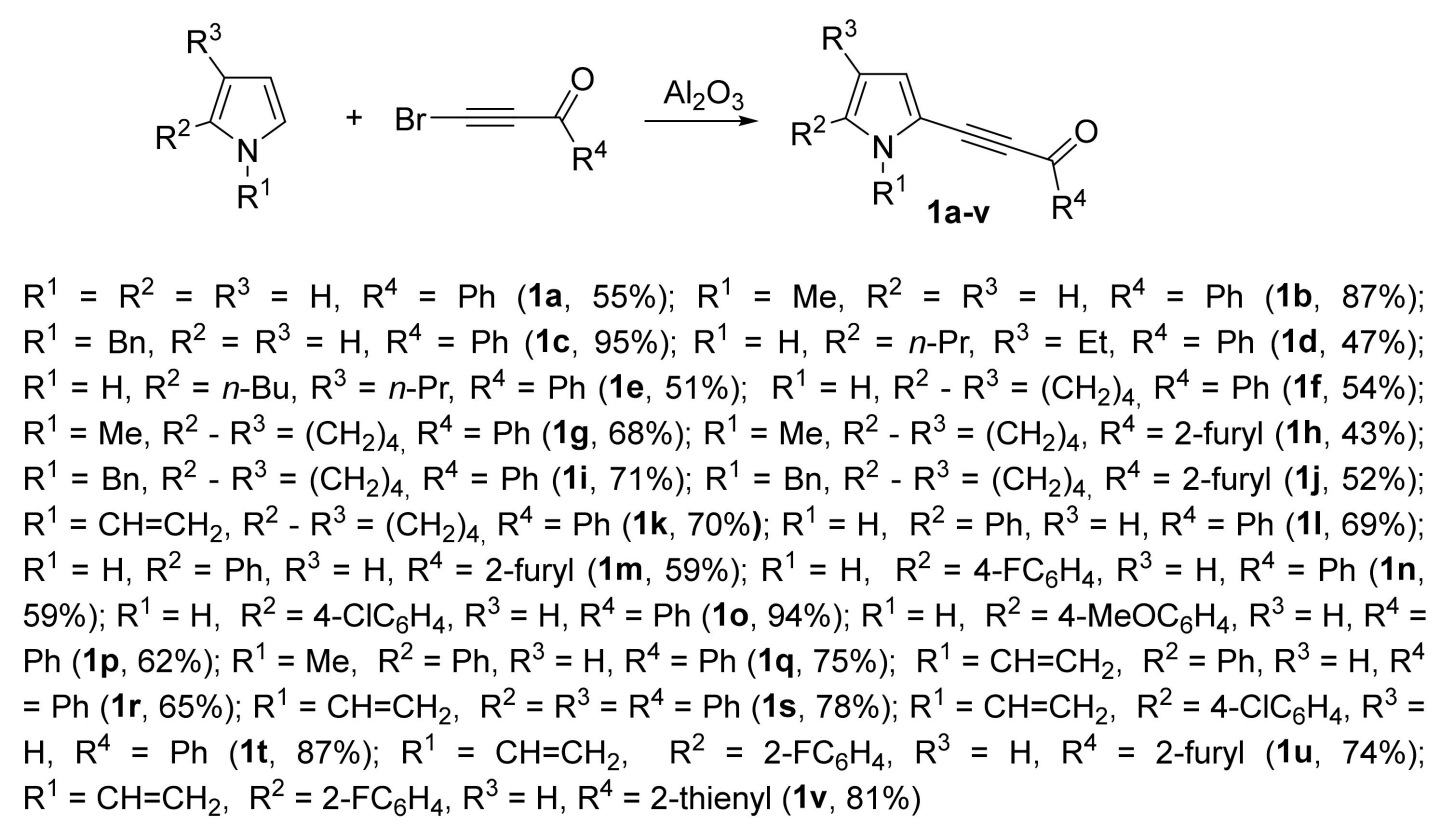
Synthesis of 2-acylethynylpyrroles.

In the present paper, we have developed an effective method for the assembly of pyrrole–aminopyrimidines via the reaction of 2-acylethynylpyrroles **1a–v**, obtained according to Scheme 2, with guanidine nitrate.

**Scheme 3:**
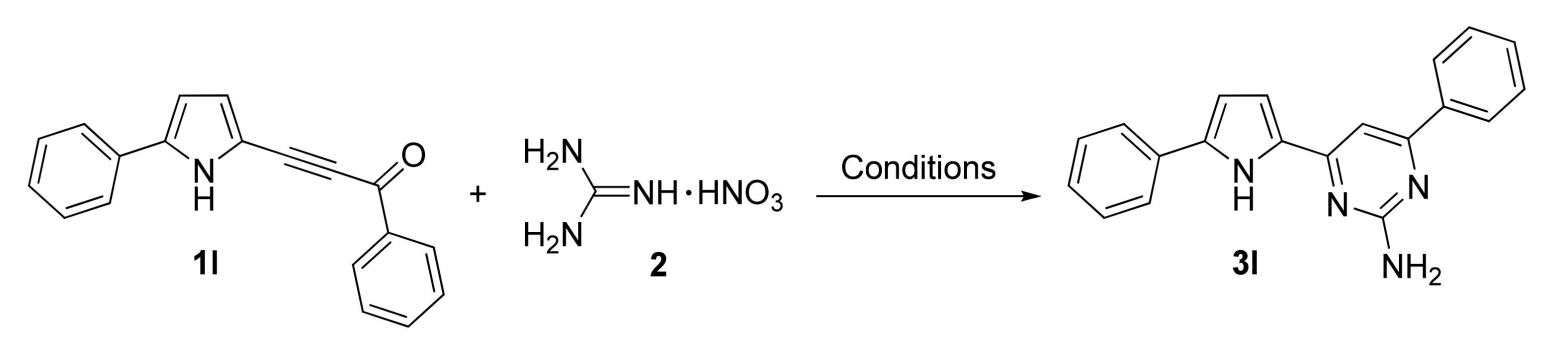
The reaction of 2-benzoylethynyl-5-phenylpyrrole (**1l**) with guanidine nitrate.

To commence the investigation, 2-benzoylethynyl-5-phenylpyrrole (**1l**, 1.0 equiv) was refluxed with guanidine nitrate (**2**, 1.0 equiv) in the presence of Na_2_CO_3_ (2.0 equiv) in MeCN for 4 h (Scheme 3). These conditions are known [36] to be effective for the synthesis of 2-aminopyrimidines from alkynones and guanidine. However, in our case, the yield of the target pyrrole–aminopyrimidine **3l** did not exceed 11%.

Therefore, in order to find optimal reaction conditions for the construction of pyrrole–aminopyrimidine ensembles, we screened combinations of bases, solvents, reagent ratio, temperature, and reaction time using the same pyrrole **1l** and guanidine nitrate as reference reagents (Scheme 3, Table 1). The reaction was carried out open to air and controlled by the ^1^H-NMR spectroscopy.

The results showed that the yield of the pyrrole–aminopyrimidine **3l** depended on the nature of the base, solvent and other reaction conditions. As seen from Table 1, among the bases tested, only KOH, Cs_2_CO_3_ and K_3_PO_4_ demonstrated a good activity (entries 11, 12, 16 and 18–22). However, in the case of the latter two bases, a significant excess of both the base and guanidine should be used to achieve a preparatively acceptable yield of aminopyrimidine **3l**. As the solvents, MeCN, *t*-BuOH, THF (tetrahydrofuran) and DMSO (dimethyl sulfoxide) were checked and it was established that yields of aminopyrimidine **3l** reached a maximum, when the reaction was carried out in DMSO in the presence of KOH (entry 22). Using this catalytic system, the effect of the ratio of reagents and reaction conditions on the yield of the target product was examined. Finally, the desired aminopyrimidine **3l** was obtained in 89% isolated yield, when 2-benzoylethynyl-5-phenylpyrrole (**1l**), guanidine and KOH in a ratio of 1:1:1.5 were reacted in DMSO at 110–115 °C for 4 h (entry 22).

**Figure 1:**
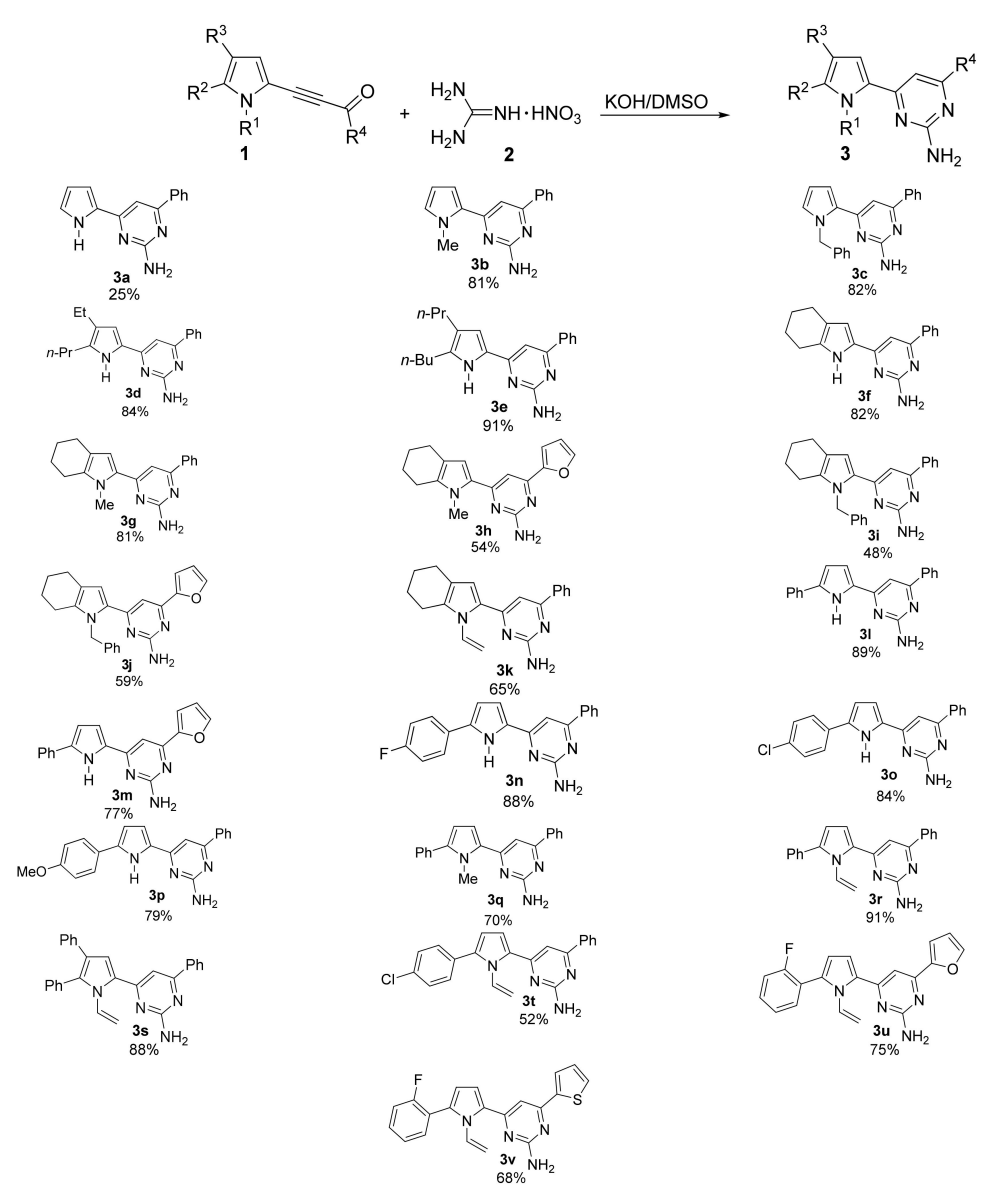
Synthesis of pyrrole–aminopyrimidine ensembles **3a**–**v** from 2-acylethynylpyrroles **1a**–**v** and guanidine nitrate. Reagents and conditions: (i) guanidine nitrate (0.40 mmol), KOH·0.5H_2_O (0.60 mmol), DMSO (8 mL), 110–115 °C, 0.5 h; (ii) 2-acylethynylpyrrole (0.40 mmol), 110–115 °C, 4 h.

Having established near to optimal reaction conditions, we have further investigated the substrate scope of this reaction (Figure 1). The experiments revealed that the reaction of 2-acylethynylpyrroles **1a**–**v** with guanidine nitrate in these conditions proceeded efficiently and selectively thus offering a short-cut to pyrrole–aminopyrimidine ensembles **3a**–**v** in good to high yields.

As follows from Figure 1, the reaction tolerates different (aliphatic, cycloaliphatic, aromatic and vinyl) substituents in the pyrrole ring, i.e., the synthesis has quite general character.

**Scheme 4:**
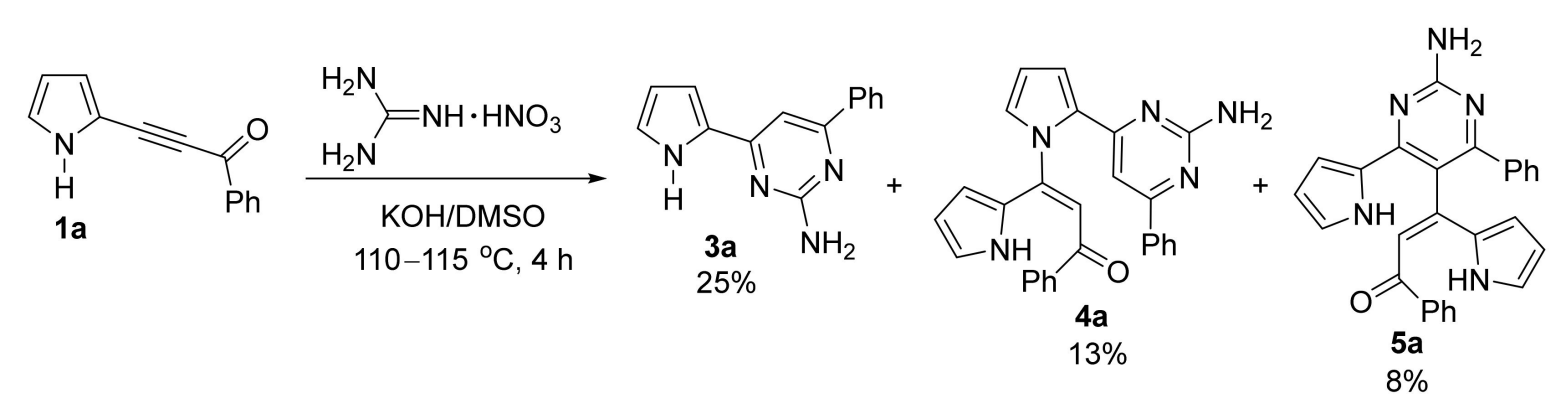
The reaction of 2-benzoylethynylpyrrole **1a** with guanidine nitrate.

The moderate yield of aminopyrimidine **3a** is due to the formation of side products: 3-[2-(2-amino-6-phenylpyrimidin-4-yl)-1*H*-pyrrol-1-yl]-1-phenyl-3-(1*H*-pyrrol-2-yl)prop-2-en-1-one (**4a**, 13%) and 3-[2-amino-4-phenyl-6-(1*H*-pyrrol-2-yl)pyrimidin-5-yl]-1-phenyl-3-(1*H*-pyrrol-2-yl)prop-2-en-1-one (**5a**, 8%) (Scheme 4). Using the reagents ratio of **1a**:**2**:KOH = 1:2:2.5 allowed to increase the yield of the aminopyrimidine **3a** to 40%, the content of adduct **4a** in this case in the reaction mixture did not exceed 6% and adduct **5** was not detected at all.

**Scheme 5:**
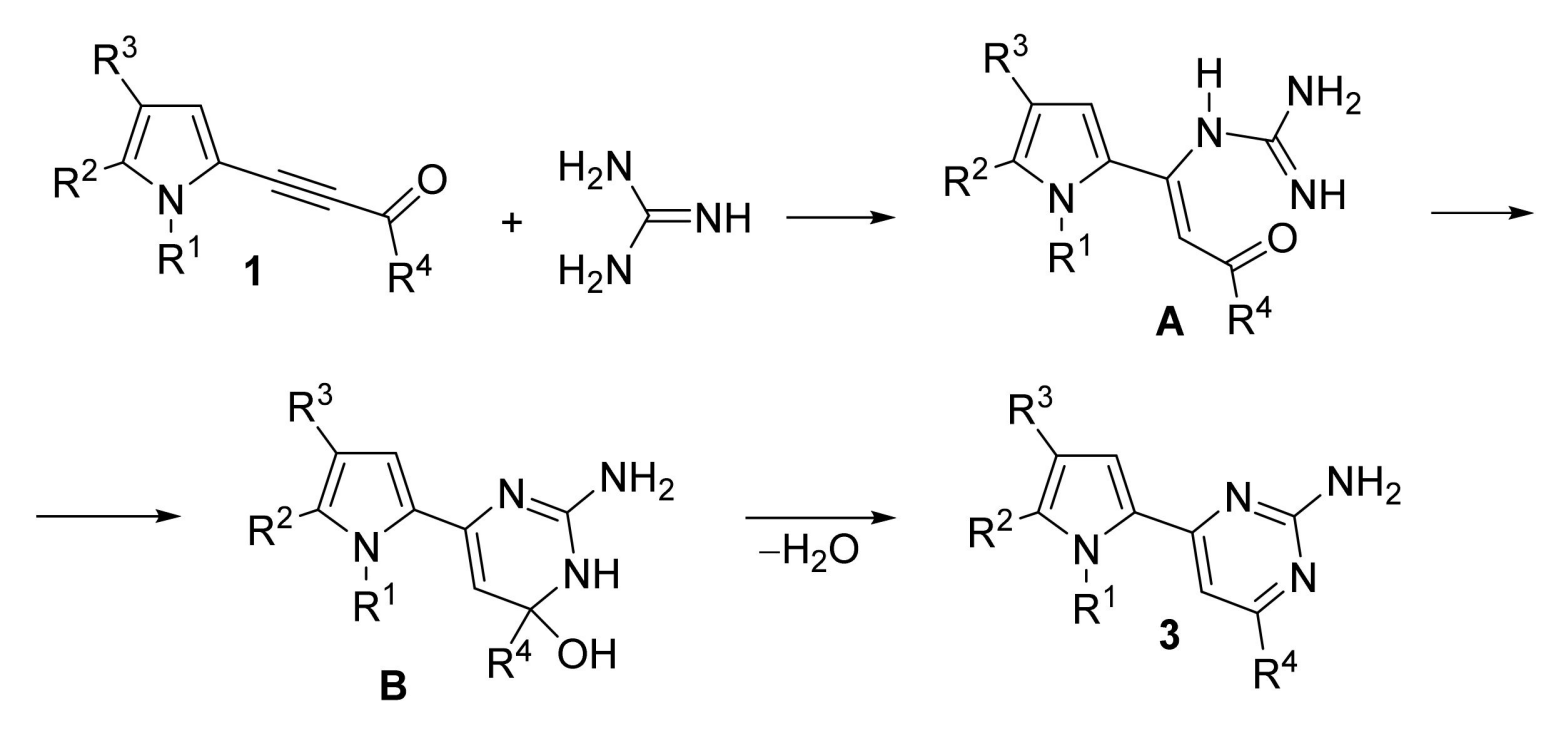
Proposed route of pyrrole–aminopyrimidine ensembles **3** formation.

The formation of pyrrole–aminopyrimidine **3**, probably, proceeds as nucleophilic addition of guanidine to the triple bond of 2-acylethynylpyrrole **1**, intramolecular cyclization of adduct **A** with participation of the carbonyl group and elimination of water from the intermediate 3,4-dihydropyrimidin-4-ol **B** (Scheme 5).

**Scheme 6:**
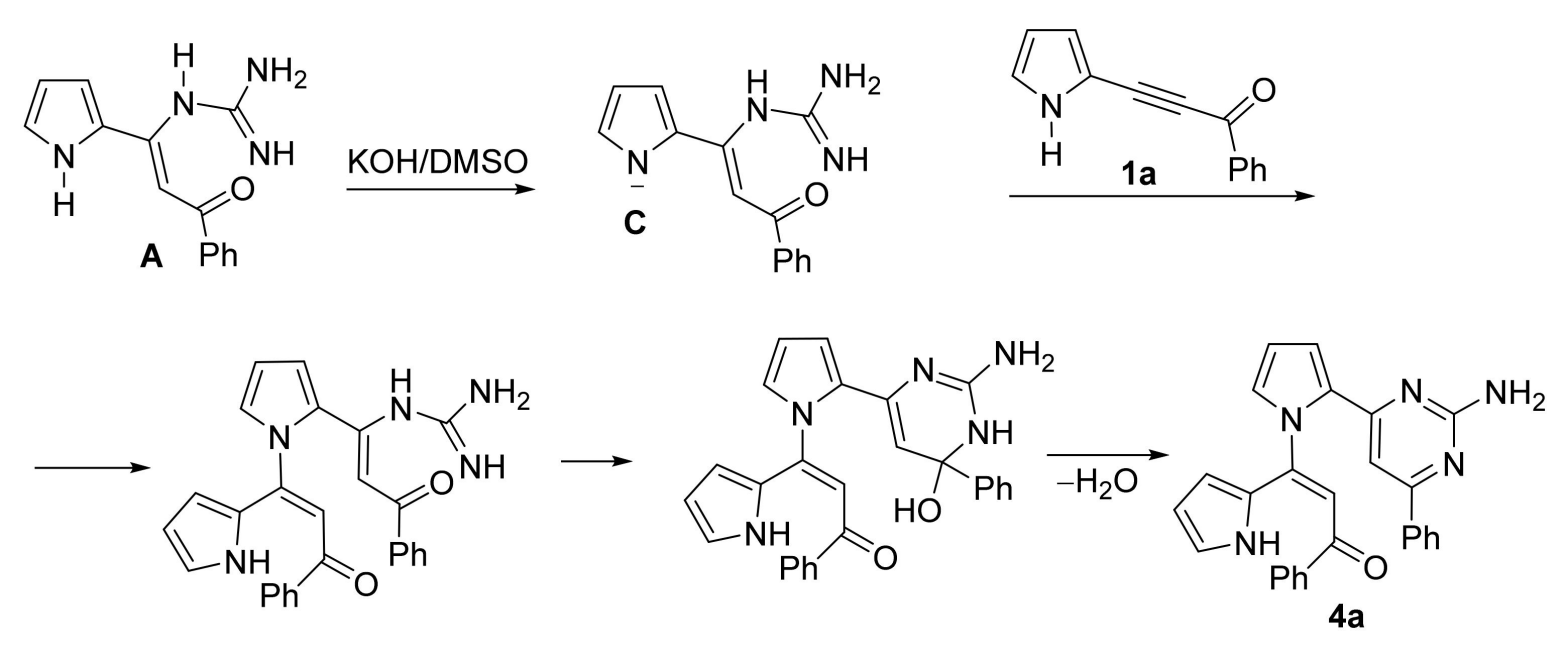
Proposed route of adduct **4a** formation.

The formation of compound **4** likely involves the nucleophilic addition of N-anion **C** of adduct **A**, existing in the reaction mixture due to the deprotonating ability of the super-base KOH/DMSO system, to the starting 2-acylethynylpyrrole **1a** (Scheme 6).

**Scheme 7:**
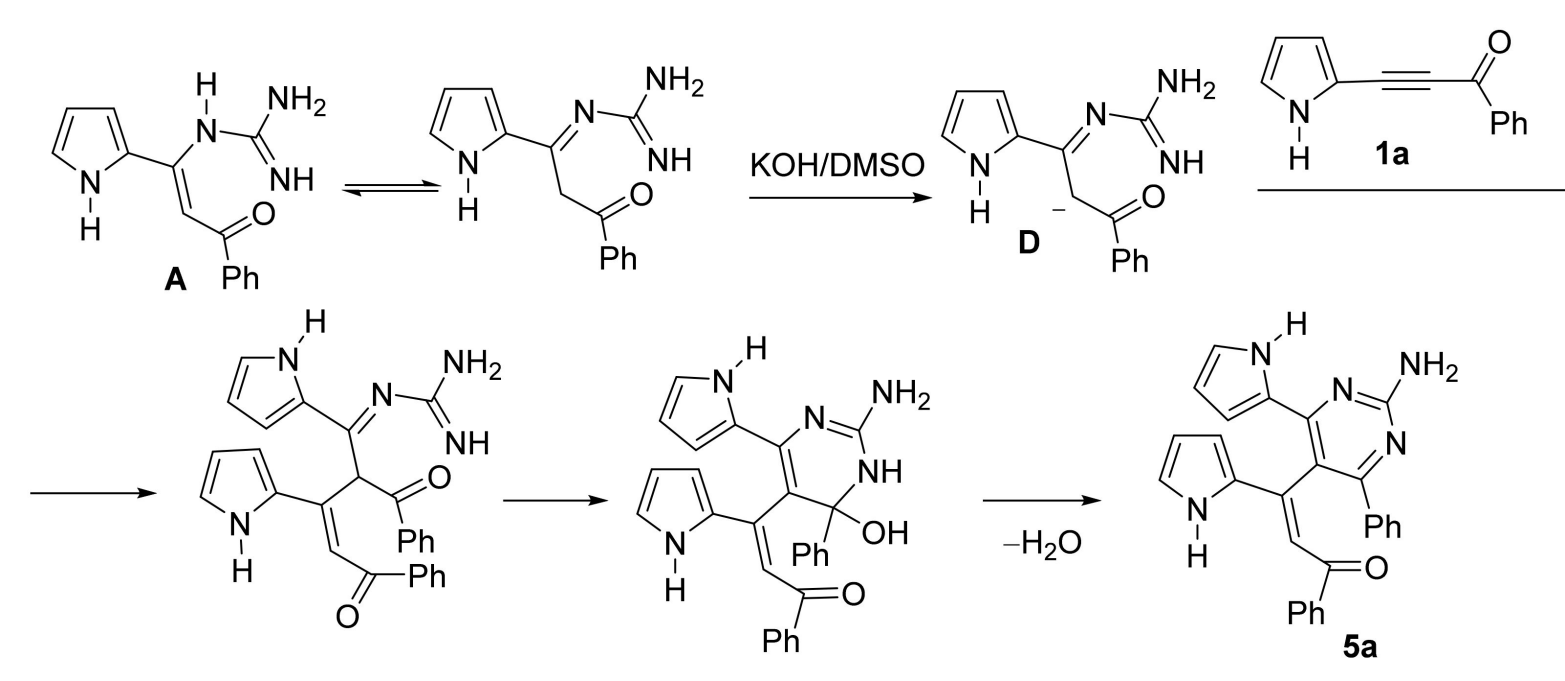
Proposed route of adduct **5a** formation.

Adduct **5**, is, probably, a result of the attack of carbocentered anion **D** of the aminopyrimidine ring to the triple bond of pyrrole **1a** (Scheme 7).

A control experiment showed that adducts **4** and **5** did not result from the direct addition of aminopyrimidine **3a** to the triple bond of the starting 2-acylethynylpyrrole **1a**.

## 3. Materials and Methods

### 3.1. General Procedures

^1^H, ^13^C, ^15^N and ^19^F NMR spectra (Nuclear magnetic resonance spectra) were recorded in CDCl_3_ and DMSO-d_6_ using a Bruker Avance 400 NMR spectrometer (Germany, 400.13, 100.6, 40.5 and 376.5 MHz, respectively). The assignment of signals in the ^1^H NMR spectra was made using COSY and NOESY experiments. Resonance signals of carbon atoms were assigned based on ^1^H-^13^C HSQC and ^1^H-^13^C HMBC experiments. The values of the δ ^15^N were measured through the 2D ^1^H-^15^N HMBC experiment. The chemical shifts (δ) are given in ppm and referenced to residual solvent: 7.27 ppm (CDCl_3_) and 2.50 ppm (DMSO-d_6_) for ^1^H, 77.1 ppm (CDCl_3_) and 39.5 ppm (DMSO-d_6_) for ^13^C and ^15^N-MeNO_2_ (0.0 ppm), respectively. The ^19^F chemical shifts were referenced to CFCl_3_. Coupling constants in hertz (Hz) were measured from one-dimensional spectra and multiplicities were abbreviated as following: br (broad), s (singlet), d (doublet), dd (doublet of doublets), m (multiplet). The chemical shifts were recorded in ppm, coupling constants (J) in Hz. ^1^H-,^13^C- and ^19^F- NMR spectra of all new synthetized molecules available in Supplementary Materials.

Infrared (IR) spectra were obtained on a Varian 3100 IF-IR spectrometer (Germany; 400–4000 cm^−1^, KBr pellets or films). The (C, H, N) microanalyses were performed on a Flash EA 1112 CHNS-O/MAS (CHN Analyzer, Thermo Fisher Scientific, Monza, Italy) instrument. The chlorine and sulfur content was determined by using the titrimetric method. Fluorine content was determined on a SPECOL 11 (Carl Zeiss, Jena, Germany) spectrophotometer. Melting points (uncorrected) were determined with melting point SMP50 (Stone, Staffordshire, UK).

### 3.2. Synthetic Procedures

2-Acylethynylpyrroles **1a,f**–**l,n**–**r** were obtained from pyrroles and 2-acylbromoacetylenes accordingly to methodology [40,41,42]. Physico-chemical characteristics 2-acylethynylpyrroles **1a,f**–**l,n**–**r** were given in [40,43,44,45,46,47]. 2-Acylethynylpyrroles **1b,c**–**e,m,s**–**v** were obtained accordingly to the following procedure:

The corresponding pyrrole (1 mmol) and acyl(bromo)acetylene (1 mmol) were carefully ground in a porcelain mortar with alumina [10-fold amount by weight of combined mass of pyrrole and acyl(bromo)acetylene] at 20–25 °C for 5 min. The reaction mixture was left for 2 h. Then the mixture was subjected to column chromatography (alumina, eluent–*n*-hexane/diethyl ether, gradient 1:0–1:1); this afforded pure 2-acylethynylpyrroles **1b,c**–**e,m,s**–**v**.

*3-(1-Methyl-1H-pyrrol-2-yl)-1-phenylprop-2-yn-1-one* (**1b**): 182 mg (87%), yellow crystals, m.p. 59 °C; ^1^H-NMR (400.13 MHz, CDCl_3_) δ: 8.20–8.18 (m, 2H, H-2,6, COPh), 7.64–7.61 (m, 1H, H-4, COPh), 7.54–7.50 (m, 2H, H-3,5, COPh), 6.86–6.85 (m, 2H, H-3,5, pyrrole), 6.21–6.20 (m, 1H, H-4, pyrrole), 3.85 (s, 3H, NMe); ^13^C-NMR (100.6 MHz, CDCl_3_) δ: 176.9, 136.7, 133.4, 128.8 (2C), 128.3 (2C), 127.6, 120.9, 112.5, 109.4, 94.7, 87.4, 34.6; IR (KBr) ν: 3114, 2936, 2362, 2168, 1630, 1448, 1326, 1255, 1173, 1035, 998, 729, 695, 649. Anal. Calcd. for C_14_H_11_NO: C, 80.36%; H, 5.30%; N, 6.69%. Found: C, 80.12%; H, 5.03%; N, 6.37%.

*3-(1-Benzyl-1H-pyrrol-2-yl)-1-phenylprop-2-yn-1-one* (**1c**): 271 mg (95%), light yellow crystals, m.p. 111 °C; ^1^H-NMR (400.13 MHz, CDCl_3_) δ: 8.07–8.04 (m, 2H, H-2,6, COPh), 7.61–7.57 (m, 1H, H-4, COPh), 7.47–7.43 (m, 2H, H-3,5, COPh), 7.39–7.35 (m, 2H, H-3,5 Ph), 7.33–7.27 (m, 1H, H-4, Ph), 7.23–7.21 (m, 2H, H-2,6, Ph), 6.92–6.91 (m, 2H, H-3,5, pyrrole), 6.28–6.27 (m, 1H, H-4, pyrrole), 5.34 (s, 2H, CH_2_); ^13^C-NMR (100.6 MHz, CDCl_3_) δ: 177.4, 137.1, 133.7, 129.2 (2C), 129.0 (2C), 128.9, 128.6 (2C), 128.0, 127.2 (2C), 126.9, 121.4, 112.9, 110.3, 95.1, 87.3, 51.9; IR (KBr) ν: 3115, 3061, 3027, 2170, 1612, 1572, 1470, 1445, 1412, 1329, 1308, 1260, 1218, 1165, 1072, 1000, 748, 730, 697, 651. Anal. Calcd. for C_20_H_15_NO: C, 84.19%; H, 5.30%; N, 4.91%. Found: C, 84.12%; H, 5.37%; N, 4.87%.

*3-(4-Ethyl-5-propyl-1H-pyrrol-2-yl)-1-phenylprop-2-yn-1-one* (**1d**): 125 mg (47%), yellow crystals, m.p. 162 °C; ^1^H-NMR (400.13 MHz, CDCl_3_) δ: 8.57 (br s, 1H, NH), 8.19–8.16 (m, 2H, H-2,6, Ph), 7.61–7.58 (m, 1H, H-4, Ph), 7.51–7.47 (m, 2H, H-3,5, Ph), 6.74 (d, *J* = 2.3 Hz, 1H, H-3, pyrrole), 2.61–2.57 (m, 2H, CH_2_), 2.47–2.41 (m, 2H, CH_2_), 1.69–1.60 (m, 2H, CH_2_), 1.21–1.17 (m, 3H, CH_3_), 0.99–0.96 (m, 3H, CH_3_); ^13^C-NMR (100.6 MHz, CDCl_3_) δ: 177.7, 137.2, 136.2, 133.6, 129.3 (2C), 128.5 (2C), 124.8, 121.2, 107.1, 93.7, 91.5, 28.1, 22.8, 18.8, 15.3, 13.9; IR (KBr) ν: 3438, 2955, 2867, 2430, 2362, 2160, 1601, 1564, 1473, 1345, 1256, 1164, 1033, 829, 692, 645. Anal. Calcd. for C_18_H_19_NO: C, 81.47%; H, 7.22%; N, 5.28%. Found: C, 81.23%; H, 7.08%; N, 5.19%.

*3-(5-Butyl-4-propyl-1H-pyrrol-2-yl)-1-phenylprop-2-yn-1-one* (**1e**): 150 mg (51%), yellow crystals, m.p. 62–63 °C; ^1^H-NMR (400.13 MHz, CDCl_3_) δ: 8.57 (br s, 1H, NH), 8.19–8.17 (m, 2H, H-2,6, Ph), 7.61–7.58 (m, 1H, H-4, Ph), 7.51–7.48 (m, 2H, H-3,5, Ph), 6.71 (d, *J* = 2.3 Hz, 1H, H-3, pyrrole), 2.62–2.59 (m, 2H, CH_2_), 2.40–2.36 (m, 2H, CH_2_), 1.61–1.55 (m, 4H, 2CH_2_), 1.41–1.33 (m, 2H, CH_2_), 0.98–0.93 (m, 6H, 2CH_3_); ^13^C-NMR (100.6 MHz, CDCl_3_) δ: 177.7, 137.1, 137.0, 133.4, 129.2 (2C), 128.4 (2C), 122.9, 121.9, 106.9, 93.9, 92.4, 31.6, 27.6, 25.8, 24.0, 22.4, 13.9, 13.7; IR (film) ν: 3298, 2956, 2928, 2865, 2377, 2157, 1614, 1567, 1469, 1318, 1241, 1164, 1040, 976, 823, 698, 646. Anal. Calcd. for C_20_H_23_NO: C, 81.87%; H, 7.90%; N, 4.77%. Found: C, 81.64%; H, 7.55%; N, 4.70%.

*1-(Furan-2-yl)-3-(5-phenyl-1H-pyrrol-2-yl)prop-2-yn-1-one* (**1m**): 154 mg (59%), red crystals, m.p. 164 °C; ^1^H-NMR (400.13 MHz, CDCl_3_) δ: 9.15 (br s, 1H, NH), 7.69–7.68 (m, 1H, H-5, furyl), 7.57–7.55 (m, 2H, H-2,6, Ph), 7.45–7.39 (m, 3H, H-3,4,5, Ph), 7.34–7.30 (m, 1H, H-3, furyl), 6.91 (dd, *J* = 2.5, 3.8 Hz, 1H, H-3, pyrrole), 6.60–6.57 (m, 2H, H-4, furyl, H-4, pyrrole); ^13^C-NMR (100.6 MHz, CDCl_3_) δ: 164.7, 153.2, 147.7, 137.7, 131.0, 129.2 (2C), 128.1, 124.8 (2C), 122.6, 120.1, 112.7, 110.7, 108.4, 92.5, 88.1; IR (KBr) ν: 3311, 2172, 1661, 1608, 1550, 1457, 1388, 1258, 1160, 1043, 972, 910, 760, 695, 593. Anal. Calcd. for C_17_H_11_NO_2_: C, 78.15%; H, 4.24%; N, 5.36%. Found: C, 78.04%; H, 4.13%; N, 5.22%.

*3-(4,5-Diphenyl-1-vinyl-1H-pyrrol-2-yl)-1-phenylprop-2-yn-1-one* (**1s**): 291 mg (78%), yellow crystals, m.p. 106 °C; ^1^H-NMR (400.13 MHz, CDCl_3_) δ: 8.21–8.20 (m, 2H, Ph), 7.65–7.62 (m, 1H, Ph), 7.55–7.51 (m, 2H, Ph), 7.42–7.40 (m, 3H, Ph), 7.33–7.31 (m, 2H, Ph), 7.23–7.15 (m, 6H, Ph, H-3, pyrrole), 6.79 (dd, *J* = 9.0, 15.8 Hz, 1H, H_X_), 5.19 (d, *J* = 15.8 Hz, 1H, H_B_), 5.67 (d, *J* = 9.0 Hz, 1H, H_A_); ^13^C-NMR (100.6 MHz, CDCl_3_) δ: 177.4, 137.1, 135.3, 134.2, 133.8, 131.0 (2C), 130.9, 130.6, 129.4 (2C), 128.8, 128.7 (2C), 128.6 (2C), 128.4 (2C), 128.1 (2C), 126.5, 125.2, 123.1, 111.9, 108.8, 95.4, 87.8; IR (KBr) ν: 3060, 2162, 1617, 1575, 1455, 1385, 1310, 1269, 1165, 1008, 821, 767, 697. Anal. Calcd. for C_27_H_19_NO: C, 86.84%; H, 5.13%; N, 3.75%. Found: C, 86.61%; H, 5.03%; N, 3.88%.

*3-(5-(4-Chlorophenyl)-1-vinyl-1H-pyrrol-2-yl)-1-phenylprop-2-yn-1-one* (**1t**): 289 mg (87%), yellow crystals, m.p. 100–102 °C; ^1^H-NMR (400.13 MHz, CDCl_3_) δ: 8.19–8.17 (m, 2H, H-2,6, COPh), 7.64–7.61 (m, 1H, H-4, COPh), 7.54–7.50 (m, 2H, H-3,5, COPh), 7.41–7.40 (m, 4H, H-2,3,5,6, 4-Cl-C_6_H_4_), 7.02 (d, *J* = 3.9 Hz, 1H, H-3, pyrrole), 6.88 (dd, *J* = 8.8, 15.8 Hz, 1H, H_X_), 6.36 (d, *J* = 3.9 Hz, 1H, H-4, pyrrole), 5.69 (d, *J* = 15.8 Hz, 1H, H_B_), 5.32 (d, *J* = 8.8 Hz, 1H, H_A_); ^13^C-NMR (100.6 MHz, CDCl_3_) δ: 177.4, 137.8, 137.0, 134.4, 133.9, 130.7, 130.2 (2C), 129.9, 129.4 (2C), 128.9 (2C), 128.6 (2C), 123.4, 113.6, 111.7, 110.3, 95.4, 87.7; IR (film) ν: 3063, 2236, 2172, 1630, 1458, 1313, 1259, 1093, 993, 780, 698. Anal. Calcd. for C_21_H_14_NOCl: C, 76.02%; H, 4.25%; Cl, 10.68%; N, 4.22%. Found: C, 75.91%; H, 4.11%; Cl, 10.57%; N, 4.37%.

*3-(5-(2-Fluorophenyl)-1-vinyl-1H-pyrrol-2-yl)-1-(furan-2-yl)prop-2-yn-1-one* (**1u**): 226 mg (74%), yellow crystals, m.p. 55–57 °C; ^1^H-NMR (400.13 MHz, CDCl_3_) δ: 7.68–7.67 (m, 1H, H-5, furyl), 7.44–7.35 (m, 3H, H-4,5,6, 2-F-C_6_H_4_), 7.25–7.15 (m, 2H, H-3, 2-F-C_6_H_4_, H-3, furyl), 7.01 (d, *J* = 3.8 Hz, 1H, H-3, pyrrole), 6.89 (dd, *J* = 8.9, 15.8 Hz, 1H, H_X_), 6.60 (dd, *J* = 1.8, 3.7 Hz, 1H, H-4, furyl), 6.37 (d, *J* = 3.8 Hz, 1H, H-4, pyrrole), 5.57 (d, *J* = 15.8 Hz, 1H, H_B_), 5.14 (d, *J* = 8.9 Hz, 1H, H_A_); ^13^C-NMR (100.6 MHz, CDCl_3_) δ: 164.1, 159.3 (d, ^1^*J*_CF_ = 249.2 Hz, C-2, 2-F-C_6_H_4_), 152.8, 147.5, 132.5, 131.5, 130.5 (d, ^3^*J*_CF_ = 8.3 Hz, C-4, 2-F-C_6_H_4_), 130.2 (d, ^4^*J*_CF_ = 2.4 Hz, C-5, 2-F-C_6_H_4_), 124.1 (d, ^3^*J*_CF_ = 3.7 Hz, C-6, 2-F-C_6_H_4_), 123.0, 119.8, 119.1 (d, ^2^*J*_CF_ = 14.9 Hz, C-1, 2-F-C_6_H_4_), 115.9 (d, ^2^*J*_CF_ = 21.6 Hz, C-3, 2-F-C_6_H_4_), 112.8, 112.5 (2C), 108.0, 94.1, 86.3; ^19^F-NMR (376.5 MHz, CDCl_3_) δ: −112.8. IR (KBr) ν: 2925, 2361, 2175, 1626, 1458, 1394, 1280, 1037, 759. Anal. Calcd. for C_19_H_12_FNO_2_: C, 74.75%; H, 3.96%; F, 6.22%; N, 4.59%. Found: C, 74.68%; H, 3.77%; F, 6.18%; N, 4.63%.

*3-(5-(2-Fluorophenyl)-1-vinyl-1H-pyrrol-2-yl)-1-(thiophen-2-yl)prop-2-yn-1-one* (**1v**): 260 mg (81%), yellow crystals, m.p. 67–69 °C; ^1^H-NMR (400.13 MHz, CDCl_3_) δ: 7.94 (dd, *J* = 1.0, 3.8 Hz, 1H, H-3, thienyl), 7.72 (dd, *J* = 1.0, 4.9 Hz, 1H, H-5, thienyl), 7.44–7.38 (m, 2H, H-4,6, 2-F-C_6_H_4_), 7.25–7.15 (m, 3H, H-3,5, 2-F-C_6_H_4_, H-4, thienyl), 7.02 (d, *J* = 3.8 Hz, 1H, H-3, pyrrole), 6.90 (dd, *J* = 8.8, 15.8 Hz, 1H, H_X_), 6.39 (d, *J* = 3.8 Hz, 1H, H-4, pyrrole), 5.53 (d, *J* = 15.8 Hz, 1H, H_B_), 5.16 (d, *J* = 8.8 Hz, 1H, H_A_); ^13^C-NMR (100.6 MHz, CDCl_3_) δ: 168.9, 159.4 (d, ^1^*J*_CF_ = 249.2 Hz, C-2, 2-F-C_6_H_4_), 144.7, 134.6, 134.2, 132.4, 131.5 (d, ^4^*J*_CF_ = 2.9 Hz, C-5, 2-F-C_6_H_4_), 130.5 (d, ^3^*J*_CF_= 7.9 Hz, C-4, 2-F-C_6_H_4_), 130.3, 128.2, 124.2 (d, ^3^*J*_CF_ = 3.7 Hz, C-6, 2-F-C_6_H_4_), 123.0, 119.2 (d, ^2^*J*_CF_ = 14.5 Hz, C-1, 2-F-C_6_H_4_), 115.9 (d, ^2^*J*_CF_ = 22.0 Hz, C-3, 2-F-C_6_H_4_), 112.9, 112.7, 108.5, 94.2, 85.9; ^19^F-NMR (376.5 MHz, CDCl_3_) δ: −112.6. IR (KBr) ν: 2925, 2859, 2361, 2170, 1603, 1462, 1409, 1267, 1226, 1045, 966, 760, 716. Anal. Calcd. for C_19_H_12_FNOS: C, 71.01%; H, 3.76%; F, 5.91%; N, 4.36%; S, 9.98%. Found: C, 70.88%; H, 3.66%; F, 5.70%; N, 4.45%; S, 9.61%.

*General Procedure for the pyrrole–aminopyrimidine ensembles***3a–v***synthesis*: A mixture of guanidine nitrate (49 mg, 0.40 mmol) and KOH·0.5H_2_O (39 mg, 0.60 mmol) was stirred in DMSO (8 mL) at 110–115 °C for 30 min. Then the 2-acylethynylpyrrole **1** (0.40 mmol) was added, and the mixture was stirred for 4 h. After cooling to 20–25 °C, the reaction mixture was diluted with saturated solution of NaCl (40 mL). The precipitate was filtered off, washed with water (3 × 20 mL) and dried. The obtained pyrrole–aminopyrimidines were purified by column chromatography (SiO_2_, eluent–*n*-hexane/diethyl ether, gradient 1:0–0:1).

*4-Phenyl-6-(1H-pyrrol-2-yl)pyrimidin-2-amine* (**3a**): 24 mg (25%), beige crystals, m.p. 96 °C; ^1^H-NMR (400.13 MHz, CDCl_3_) δ: 9.75 (br s, 1H, NH), 8.04–8.02 (m, 2H, H-2,6, Ph), 7.50–7.49 (m, 3H, H-3,4,5, Ph), 7.28 (s, 1H, H-5, pyrimidine), 6.95–6.92 (m, 2H, H-3,5, pyrrole), 6.35–6.34 (m, 1H, H-4, pyrrole), 5.13 (br s, 2H, NH_2_); ^13^C-NMR (100.6 MHz, CDCl_3_) δ: 165.5 (C-6, pyrimidine), 163.3 (C-2, pyrimidine), 158.3 (C-4, pyrimidine), 137.8 (C-1, Ph), 130.4 (C-4, Ph), 129.9 (C-2, pyrrole), 128.8 (C-3,5, Ph), 127.1 (C-2,6, Ph), 121.5 (C-5, pyrrole), 110.8 (C-4, pyrrole), 110.5 (C-3, pyrrole), 101.9 (C-5, pyrimidine); ^15^N-NMR (40.5 MHz, CDCl_3_) δ: −304.9 (NH_2_), −233.8 (NH), −157.3 (N-1), −148.9 (N-3); IR (KBr) ν: 3464, 3418, 3350, 3175, 1634, 1582, 1555, 1534, 1468, 1454, 1423, 1359, 1257, 1227, 1112, 1035, 997, 911, 880, 773, 732, 701. Anal. Calcd. for C_14_H_12_N_4_: C, 71.17%; H, 5.12%; N, 23.71%. Found: C, 70.83%; H, 4.74%; N, 23.45%.

*4-(1-Methyl-1H-pyrrol-2-yl)-6-phenylpyrimidin-2-amine* (**3b**): 81 mg (81%), beige crystals, m.p. 129 °C; ^1^H-NMR (400.13 MHz, CDCl_3_) δ: 8.03–8.01 (m, 2H, H-2,6, Ph), 7.49–7.48 (m, 3H, H-3,4,5, Ph), 7.29 (s, 1H, H-5, pyrimidine), 6.85–6.84 (m, 1H, H-5, pyrrole), 6.79–6.78 (m, 1H, H-3, pyrrole), 6.21–6.20 (m, 1H, H-4, pyrrole), 5.07 (br s, 2H, NH_2_), 4.08 (s, 3H, NMe); ^13^C-NMR (100.6 MHz, CDCl_3_) δ: 165.1, 163.0, 160.6, 138.8, 130.4, 130.2, 128.7 (2C), 128.3, 127.0 (2C), 113.4, 108.2, 104.4, 37.7; IR (KBr) ν: 3478, 3319, 3199, 3059, 2956, 1628, 1570, 1528, 1487, 1455, 1433, 1381, 1345, 1216, 1117, 1090, 1057, 838, 802, 766, 736, 694, 644. Anal. Calcd. for C_15_H_14_N_4_: C, 71.98%; H, 5.64%; N, 22.38%. Found: C, 71.80%; H, 5.48%; N, 22.31%.

*4-(1-Benzyl-1H-pyrrol-2-yl)-6-phenylpyrimidin-2-amine* (**3c**): 107 mg (82%), light yellow crystals, m.p. 112–114 °C; ^1^H-NMR (400.13 MHz, CDCl_3_) δ: 7.99–7.97 (m, 2H, H-2,6, Ph), 7.47–7.46 (m, 3H, H-3,4,5, Ph), 7.31–7.26 (m, 3H, H-3,5, CH_2_Ph, H-5, pyrimidine), 7.24–7.20 (m, 1H, H-4, CH_2_Ph), 7.11–7.09 (m, 2H, H-2,6, CH_2_Ph), 6.92–6.91 (m, 1H, H-5, pyrrole), 6.89–6.88 (m, 1H, H-3, pyrrole), 6.29–6.28 (m, 1H, H-4, pyrrole), 5.82 (s, 2H, CH_2_), 4.97 (br s, 2H, NH_2_); ^13^C-NMR (100.6 MHz, CDCl_3_) δ: 165.1, 162.9, 160.4, 139.5, 138.0, 130.2, 130.1, 128.7 (2C), 128.6 (2C), 128.0, 127.2, 127.0 (2C), 126.7 (2C), 113.9, 108.9, 104.5, 52.6; IR (KBr) ν: 3493, 3319, 3197, 3113, 3023, 2926, 1625, 1569, 1537, 1479, 1453, 1430, 1407, 1359, 1229, 1114, 1087, 1025, 834, 802, 769, 739, 720, 694, 643. Anal. Calcd. for C_21_H_18_N_4_: C, 77.28%; H, 5.56%; N, 17.17%. Found: C, 77.20%; H, 5.49%; N, 17.23%.

*4-(4-Ethyl-5-propyl-1H-pyrrol-2-yl)-6-phenylpyrimidin-2-amine* (**3d**): 103 mg (84%), yellow crystals, m.p. 162 °C; ^1^H-NMR (400.13 MHz, CDCl_3_) δ: 9.19 (br s, 1H, NH), 8.02–8.01 (m, 2H, H-2,6, Ph), 7.48–7.47 (m, 3H, H-3,4,5, Ph), 7.20 (s, 1H, H-5, pyrimidine), 6.75 (d, *J* = 2.2 Hz, 1H, H-3, pyrrole), 4.95 (br s, 2H, NH_2_), 2.61–2.57 (m, 2H, CH_2_), 2.49–2.44 (m, 2H, CH_2_), 1.69–1.64 (m, 2H, CH_2_), 1.24–1.20 (m, 3H, CH_3_), 1.00–0.96 (m, 3H, CH_3_); ^13^C-NMR (100.6 MHz, CDCl_3_) δ: 165.0, 163.2, 158.1, 138.1, 133.2, 130.1, 128.7 (2C), 127.1, 127.0 (2C), 124.6, 110.7, 101.3, 28.0, 23.1, 19.0, 15.6, 13.9; IR (KBr) ν: 3483, 3307, 3184, 2959, 2927, 2867, 1631, 1589, 1566, 1536, 1501, 1458, 1420, 1370, 1324, 1237, 1188, 1072, 1006, 920, 824, 768, 696, 642. Anal. Calcd. for C_19_H_22_N_4_: C, 74.48%; H, 7.24%; N, 18.29%. Found: C, 74.31%; H, 7.11%; N, 17.98%.

*4-(5-Butyl-4-propyl-1H-pyrrol-2-yl)-6-phenylpyrimidin-2-amine* (**3e**): 122 mg (91%), yellow crystals, m.p. 98–99 °C; ^1^H-NMR (400.13 MHz, CDCl_3_) δ: 9.19 (br s, 1H, NH), 8.03–8.00 (m, 2H, H-2,6, Ph), 7.48–7.46 (m, 3H, H-3,4,5, Ph), 7.19 (s, 1H, H-5, pyrimidine), 6.72 (d, *J* = 2.2 Hz, 1H, H-3, pyrrole), 4.98 (br s, 2H, NH_2_), 2.62–2.58 (m, 2H, CH_2_), 2.43–2.39 (m, 2H, CH_2_), 1.67–1.57 (m, 4H, 2CH_2_), 1.44–1.35 (m, 2H, CH_2_), 1.00–0.93 (m, 6H, 2CH_3_); ^13^C-NMR (100.6 MHz, CDCl_3_) δ: 164.9, 163.2, 158.1, 138.1, 133.9, 130.1, 128.7 (2C), 127.0, 126.9 (2C), 122.8, 111.4, 101.3, 32.0, 28.0, 25.7, 24.3, 22.6, 14.1, 13.9; IR (KBr) ν: 3484, 3450, 3311, 3191, 2954, 2926, 2863, 1635, 1588, 1565, 1535, 1500, 1459, 1421, 1370, 1333, 1231, 1188, 1073, 1008, 919, 812, 766, 696, 644. Anal. Calcd. for C_21_H_26_N_4_: C, 75.41%; H, 7.84%; N, 16.75%. Found: C, 75.34%; H, 7.63%; N, 16.51%.

*4-Phenyl-6-(4,5,6,7-tetrahydro-1H-indol-2-yl)pyrimidin-2-amine* (**3f**): 95 mg (82%), dark orange crystals, m.p. 100–102 °C; ^1^H-NMR (400.13 MHz, CDCl_3_) δ: 9.32 (br s, 1H, NH), 8.03–8.01 (m, 2H, H-2,6, Ph), 7.48–7.47 (m, 3H, H-3,4,5, Ph), 7.20 (s, 1H, H-5, pyrimidine), 6.68 (d, *J* = 2.0 Hz, 1H, H-3, pyrrole), 5.03 (br s, 2H, NH_2_), 2.62–2.55 (m, 4H, CH_2_-4,7), 1.84–1.79 (m, 4H, CH_2_-5,6); ^13^C-NMR (100.6 MHz, CDCl_3_) δ: 165.0, 163.1, 158.3, 138.0, 132.2, 130.2, 128.7 (2C), 127.8, 127.0 (2C), 120.2, 110.0, 101.4, 23.7, 23.1, 22.9, 22.8; IR (KBr) ν: 3404, 3311, 3195, 3060, 2925, 2848, 1593, 1568, 1535, 1503, 1423, 1346, 1224, 1143, 1005, 830, 808, 770, 695. Anal. Calcd. for C_18_H_18_N_4_: C, 74.46%; H, 6.25%; N, 19.30%. Found: C, 74.14%; H, 6.05%; N, 19.18%.

*4-(1-Methyl-4,5,6,7-tetrahydro-1H-indol-2-yl)-6-phenylpyrimidin-2-amine* (**3g**): 99 mg (81%), dark yellow crystals, m.p. 182 °C; ^1^H-NMR (400.13 MHz, CDCl_3_) δ: 8.01–7.99 (m, 2H, H-2,6, Ph), 7.48–7.47 (m, 3H, H-3,4,5, Ph), 7.23 (s, 1H, H-5, pyrimidine), 6.64 (s, 1H, H-3, pyrrole), 5.04 (br s, 2H, NH_2_), 3.91 (s, 3H, NMe), 2.63–2.60 (m, 2H, CH_2_-7), 2.57–2.54 (m, 2H, CH_2_-4), 1.91–1.86 (m, 2H, CH_2_-6), 1.79–1.75 (m, 2H, CH_2_-5); ^13^C-NMR (100.6 MHz, DMSO-d_6_) δ: 163.1, 162.9, 160.3, 137.7, 134.3, 129.9, 128.4 (2C), 128.1, 126.6 (2C), 117.2, 112.0, 101.5, 32.6, 23.1, 22.7, 22.6, 21.7; IR (KBr) ν: 3489, 3376, 2941, 2915, 2363, 1604, 1585, 1561, 1530, 1495, 1449, 1401, 1369, 1223, 1183, 1147, 826, 803, 772, 695. Anal. Calcd. for C_19_H_20_N_4_: C, 74.97%; H, 6.62%; N, 18.41%. Found: C, 74.58%; H, 6.41%; N, 18.32%.

*4-(2-Furyl)-6-(1-methyl-4,5,6,7-tetrahydro-1H-indol-2-yl)pyrimidin-2-amine* (**3h**): 64 mg (54%), light brown crystals, m.p. 218 °C; ^1^H-NMR (400.13 MHz, CDCl_3_) δ: 7.56–7.55 (m, 1H, H-5, furyl), 7.17 (s, 1H, H-5, pyrimidine), 7.10–7.09 (m, 1H, H-3, furyl), 6.65 (s, 1H, H-3, pyrrole), 6.54 (dd, *J* = 1.3, 2.0 Hz, 1H, H-4, furyl), 4.99 (br s, 2H, NH_2_), 3.90 (s, 3H, NMe), 2.61–2.58 (m, 2H, CH_2_-7), 2.56–2.53 (m, 2H, CH_2_-4), 1.90–1.84 (m, 2H, CH_2_-6), 1.78–1.74 (m, 2H, CH_2_-5); ^13^C-NMR (100.6 MHz, CDCl_3_) δ: 162.7, 160.7, 155.4, 152.6, 144.1, 135.4, 128.7, 118.6, 112.3, 112.1, 110.7, 102.0, 33.0, 23.5, 23.2, 23.0, 22.5; IR (KBr) ν: 3474, 3298, 3182, 2931, 2839, 1606, 1544, 1533, 1489, 1449, 1398, 1369, 1223, 1183, 1146, 1010, 953, 800, 753, 743. Anal. Calcd. for C_17_H_18_N_4_O: C, 69.37%; H, 6.16%; N, 19.03%. Found: C, 69.14%; H, 6.05%; N, 19.04%.

*4-(1-Benzyl-4,5,6,7-tetrahydro-1H-indol-2-yl)-6-phenylpyrimidin-2-amine* (**3i**): 73 mg (48%), yellow crystals, m.p. 128–129 °C; ^1^H-NMR (400.13 MHz, CDCl_3_) δ: 8.00–7.98 (m, 2H, H-2,6, Ph), 7.49–7.48 (m, 3H, H-3,4,5, Ph), 7.33–7.22 (m, 4H, H-5, pyrimidine, H-3,4,5, CH_2_Ph), 7.06–7.04 (m, 2H, H-2,6, CH_2_Ph), 6.79 (s, 1H, H-3, pyrrole), 5.82 (s, 2H, CH_2_Ph), 4.90 (br s, 2H, NH_2_), 2.63–2.61 (m, 2H, CH_2_-7), 2.53–2.51 (m, 2H, CH_2_-4), 1.85–1.78 (m, 4H, CH_2_-5,6); ^13^C-NMR (100.6 MHz, CDCl_3_) δ: 164.6, 162.8, 160.5, 139.7, 138.2, 135.5, 130.0, 128.8, 128.7 (2C), 128.5 (2C), 127.0 (2C), 126.7, 126.2 (2C), 119.1, 112.7, 104.1, 48.6, 23.5, 23.2, 23.1, 22.4; IR (KBr) ν: 3426, 3299, 3188, 3031, 2926, 2847, 2361, 1965, 1622, 1560, 1497, 1442, 1409, 1340, 1232, 1177, 1104, 830, 768, 699. Anal. Calcd. for C_25_H_24_N_4_: C, 78.92%; H, 6.36%; N, 14.73%. Found: C, 78.83%; H, 6.30%; N, 14.80%.

*4-(1-Benzyl-4,5,6,7-tetrahydro-1H-indol-2-yl)-6-(2-furyl)pyrimidin-2-amine* (**3j**): 87 mg (59%), dark yellow crystals, m.p. 182 °C; ^1^H-NMR (400.13 MHz, CDCl_3_) δ: 7.54–7.53 (m, 1H, H-5, furyl), 7.28–7.25 (m, 2H, H-2,6, Ph), 7.21–7.18 (m, 2H, H-5, pyrimidine, H-4, Ph), 7.06–7.05 (m, 1H, H-3, furyl), 7.01–6.99 (m, 2H, H-3,5, Ph), 6.75 (s, 1H, H-3, pyrrole), 6.52 (dd, *J* = 1.8, 3.2 Hz, 1H, H-4, furyl), 5.78 (s, 2H, CH_2_Ph), 4.80 (br s, 2H, NH_2_), 2.59–2.56 (m, 2H, CH_2_-7), 2.50–2.47 (m, 2H, CH_2_-4), 1.82–1.72 (m, 4H, CH_2_-5,6); ^13^C-NMR (100.6 MHz, CDCl_3_) δ: 162.6, 160.5, 155.4, 152.6, 144.1, 139.6, 135.7, 128.5 (2C), 128.4, 126.7, 126.2 (2C), 119.1, 113.0, 112.1, 110.6, 101.8, 48.5, 23.5, 23.1, 23.0, 22.4; IR (KBr) ν: 3315, 3178, 2997, 2935, 2843, 1643, 1603, 1549, 1495, 1458, 1406, 1377, 1242, 1180, 1146, 1103, 1019, 951, 810, 764, 734, 694. Anal. Calcd. for C_23_H_22_N_4_O: C, 74.57%; H, 5.99%; N, 15.12%. Found: C, 74.28%; H, 5.78%; N, 15.04%.

*4-Phenyl-6-(1-vinyl-4,5,6,7-tetrahydro-1H-indol-2-yl)pyrimidin-2-amine* (**3k**): 82 mg (65%), yellow crystals, m.p. 118 °C; ^1^H-NMR (400.13 MHz, CDCl_3_) δ: 8.01–7.99 (m, 2H, H-2,6 Ph), 7.51–7.44 (m, 4H, H-3,4,5, Ph, H_X_), 7.23 (s, 1H, H-5, pyrimidine), 6.68 (s, 1H, H-3, pyrrole), 5.10 (br s, 2H, NH_2_), 5.04 (d, *J* = 16.0 Hz, 1H, H_B_), 5.01 (d, *J* = 8.9Hz, 1H, H_A_), 2.75–2.72 (m, 2H, CH_2_-7), 2.59–2.56 (m, 2H, CH_2_-4), 1.86–1.76 (m, 4H, CH_2_-6, CH_2_-5); ^13^C-NMR (100.6 MHz, CDCl_3_) δ: 165.0, 163.0, 160.2, 138.1, 134.1, 133.1, 130.2, 129.8, 128.8 (2C), 127.0 (2C), 120.4, 113.7, 105.4, 105.0, 24.8, 23.6, 23.2, 23.1; IR (KBr) ν: 3485, 3312, 3199, 2927, 2847, 1636, 1564, 1537, 1501, 1459, 1397, 1368, 1227, 1184, 1145, 980, 861, 827, 766, 698, 642. Anal. Calcd. for C_20_H_20_N_4_: C, 75.92%; H, 6.37%; N, 17.71%. Found: C, 75.87%; H, 6.33%; N, 17.81%. 

*4-Phenyl-6-(5-phenyl-1H-pyrrol-2-yl)pyrimidin-2-amine* (**3l**): 111 mg (89%), brown crystals, m.p. 194–196 °C; ^1^H-NMR (400.13 MHz, DMSO-d_6_) δ: 11.55 (br s, 1H, NH), 8.17–8.15 (m, 2H, Ph), 7.88–7.85 (m, 2H, Ph), 7.68 (s, 1H, H-5, pyrimidine), 7.53–7.52 (m, 4H, Ph), 7.28–7.24 (m, 2H, Ph), 7.06–7.05 (m, 1H, H-3, pyrrole), 6.66–6.65 (m, 1H, H-4, pyrrole), 6.47 (br s, 2H, NH_2_); ^13^C-NMR (100.6 MHz, DMSO-d_6_) δ: 163.9, 163.7, 158.1, 137.5, 134.4, 131.8, 130.2, 128.6 (2C), 128.5 (2C), 126.7 (2C), 126.6 (2C), 115.6, 115.4, 112.5, 108.2, 99.9; IR (KBr) ν: 3442, 3369, 3274, 3161, 3058, 1602, 1570, 1537, 1457, 1432, 1366, 1301, 1235, 1071, 1046, 1002, 912, 836, 755, 691, 614. Anal. Calcd. for C_20_H_16_N_4_: C, 76.90%; H, 5.16%; N, 17.94%. Found: C, 76.75%; H, 5.04%; N, 17.88%.

*4-(2-Furyl)-6-(5-phenyl-1H-pyrrol-2-yl)pyrimidin-2-amine* (**3m**): 93 mg (77%), brown crystals, m.p. 208–210 °C; ^1^H-NMR (400.13 MHz, DMSO-d_6_) δ: 11.59 (br s, 1H, NH), 7.92–7.91 (m, 1H, H-5, furyl), 7.85–7.83 (m, 2H, H-2,6, Ph), 7.50 (s, 1H, H-5, pyrimidine), 7.43–7.39 (m, 2H, H-3,5, Ph), 7.27–7.23 (m, 1H, H-4, Ph), 7.16–7.15 (m, 1H, H-3, furyl), 7.01–7.00 (m, 1H, H-4, furyl), 6.71–6.69 (m, 2H, H-3,4, pyrrole), 6.50 (br s, 2H, NH_2_); ^13^C-NMR (100.6 MHz, DMSO-d_6_) δ: 163.5, 158.1, 155.7, 152.5, 145.0, 135.5, 131.9, 131.7 128.7 (2C), 126.7, 124.7 (2C), 112.6, 112.5, 110.9, 108.3, 98.1; IR (KBr) ν: 3482, 3454, 3298, 3181, 2924, 1631, 1588, 1566, 1541, 1445, 1386, 1351, 1299, 1237, 1214, 1160, 1044, 1011, 879, 812, 750, 681, 595, 554, 424. Anal. Calcd. for C_18_H_14_N_4_O: C, 71.51%; H, 4.67%; N, 18.53%. Found: C, 71.39%; H, 4.59%; N, 18.65%.

*4-[5-(4-Fluorophenyl)-1H-pyrrol-2-yl]-6-phenylpyrimidin-2-amine* (**3n**): 116 mg (88%), light yellow crystals, m.p. 221–222 °C; ^1^H-NMR (400.13 MHz, CDCl_3_) δ: 9.75 (br s, 1H, NH), 8.05–8.03 (m, 2H, H-2,6, Ph), 7.59–7.56 (m, 2H, H-2,6, 4-F-C_6_H_4_), 7.51–7.49 (m, 3H, H-3,4,5, Ph), 7.29 (s, 1H, H-5, pyrimidine), 7.14–7.09 (m, 2H, H-3,5, 4-F-C_6_H_4_), 6.96 (dd, *J* = 0.8, 2.6 Hz, 1H, H-3, pyrrole), 6.57 (dd, *J* = 0.8, 2.9 Hz, 1H, H-4, pyrrole), 5.07 (br s, 2H, NH_2_); ^13^C NMR (100.6 MHz, DMSO-d_6_) δ: 163.5 (C-2, pyrimidine), 163.3 (C-6, pyrimidine), 160.7 (d, ^1^*J*_CF_ = 243.9 Hz, C-4, 4-F-C_6_H_4_), 157.7 (C-4, pyrimidine), 137.1 (C-1, Ph), 134.0 (C-5, pyrrole), 131.5 (C-2, pyrrole), 129.8 (C-4, Ph), 128.2 (d, ^4^*J*_CF_ = 2.9 Hz, C-1, 4-F-C_6_H_4_), 128.1 (C-3,5, Ph), 126.3 (C-2,6, Ph), 126.2 (d, ^3^*J*_CF_ = 8.7 Hz, C-2,6, 4-F-C_6_H_4_), 115.1 (d, ^2^*J*_CF_ = 21.5 Hz, C-3,5, 4-F-C_6_H_4_), 112.0 (C-3, pyrrole), 107.8 (C-4, pyrrole), 99.5 (C-5, pyrimidine); ^15^N-NMR (40.5 MHz, DMSO-d_6_) δ: −298.9 (NH_2_), −233.8 (NH, pyrrole), −151.2 (N-3), −147.4 (N-1); IR (KBr) ν: 3445, 3388, 3270, 3159, 1602, 1575, 1524, 1478, 1451, 1433, 1366, 1301, 1233, 1157, 1047, 832, 765, 697. Anal. Calcd. for C_20_H_15_FN_4_: C, 72.71%; H, 4.58%; F, 5.75%; N, 16.96%. Found: C, 72.62%; H, 4.48%; F, 5.60%; N, 17.07%.

*4-[5-(4-Chlorophenyl)-1H-pyrrol-2-yl]-6-phenylpyrimidin-2-amine* (**3o**): 117 mg (84%), brown crystals, m.p. 226 °C; ^1^H-NMR (400.13 MHz, CDCl_3_) δ: 9.80 (br s, 1H, NH), 8.05–8.03 (m, 2H, H-2,6, Ph), 7.54–7.49 (m, 5H, H-3,4,5, Ph, H-2,6, 4-Cl-C_6_H_4_), 7.39–7.37 (m, 2H, H-3,5, 4-Cl-C_6_H_4_), 7.29 (s, 1H, H-5, pyrimidine), 6.96–6.95 (m, 1H, H-3, pyrrole), 6.62–6.61 (m, 1H, H-4, pyrrole), 5.11 (br s, 2H, NH_2_); ^13^C-NMR (100.6 MHz, DMSO-d_6_) δ: 164.0, 163.7, 158.1, 137.5, 134.1, 132.3, 131.0, 130.9, 130.3, 128.7 (2C), 128.6 (2C), 126.8 (2C), 126.3 (2C), 112.6, 108.9, 100.1; IR (KBr) ν: 3434, 2925, 2858, 1622, 1583, 1531, 1470, 1432, 1360, 1290, 1231, 1113, 1095, 1048, 829, 770, 692; Anal. Calcd. for C_20_H_15_ClN_4_: C, 69.26%; H, 4.36%; Cl, 10.22%; N, 16.15%. Found: C, 69.13%; H, 4.28%; Cl, 10.14%; N, 16.09%.

*4-[5-(4-Methoxyphenyl)-1H-pyrrol-2-yl]-6-phenylpyrimidin-2-amine* (**3p**): 108 mg (79%), yellow crystals, m.p. 217 °C; ^1^H-NMR (400.13 MHz, CDCl_3_) δ: 9.72 (br s, 1H, NH), 8.05–8.02 (m, 2H, H-2,6, Ph), 7.56–7.54 (m, 2H, H-2,6, 4-MeO-C_6_H_4_), 7.50–7.49 (m, 3H, H-3,4,5, Ph), 7.28 (s, 1H, H-5, pyrimidine), 6.97–6.95 (m, 3H, H-3,5, 4-MeO-C_6_H_4_, H-3, pyrrole), 6.54 (dd, *J* = 0.6, 2.9 Hz, 1H, H-4, pyrrole), 5.06 (br s, 2H, NH_2_), 3.85 (s, 3H, MeO); ^13^C-NMR (100.6 MHz, DMSO-d_6_) δ: 163.9, 163.8, 158.4, 158.3, 137.7, 135.7, 131.1, 130.4, 128.7 (2C), 126.8 (2C), 126.2 (2C), 124.9, 114.3 (2C), 112.8, 107.4, 99.9, 55.3; IR (KBr) ν: 3484, 3430, 3298, 3144, 2953, 2928, 1634, 1569, 1530, 1479, 1455, 1433, 1359, 1251, 1178, 1048, 1030, 1005, 833, 774, 706, 645. Anal. Calcd. for C_21_H_18_N_4_O: C, 73.67%; H, 5.30%; N, 16.36%. Found: C, 73.59%; H, 5.24%; N, 16.27%.

*4-Phenyl-6-(1-methyl-5-phenyl-1H-pyrrol-2-yl)pyrimidin-2-amine* (**3q**): 91 mg (70%), yellow crystals, m.p. 60–62 ^o^C; ^1^H-NMR (400.13 MHz, CDCl_3_) δ: 8.05–8.03 (m, 2H, H-2,6, Ph), 7.49–7.44 (m, 7H, Ph), 7.40–7.36 (m, 1H, H-4, Ph), 7.33 (s, 1H, H-5, pyrimidine), 6.90 (d, *J* = 3.8 Hz, 1H, H-3, pyrrole), 6.32 (d, *J* = 3.8 Hz, 1H, H-4, pyrrole), 5.11 (br s, 2H, NH_2_), 3.98 (s, 3H, NMe); ^13^C-NMR (100.6 MHz, CDCl_3_) δ: 165.2, 163.0, 160.7, 140.8, 138.1, 132.9, 132.6, 130.3, 129.3 (2C), 128.8 (2C), 128.6 (2C), 127.6, 127.1 (2C), 113.5, 109.6, 105.1, 35.7; IR (KBr) ν: 3476, 3397, 3303, 3182, 3059, 1565, 1528, 1455, 1293, 1347, 1312, 1221, 1113, 1087, 1027, 989, 919, 836, 757, 695, 642. Anal. Calcd. for C_21_H_18_N_4_: C, 77.28%; H, 5.56%; N, 17.17%. Found: C, 77.03%; H, 5.32%; N, 17.09%.

*4-Phenyl-6-(5-phenyl-1-vinyl-1H-pyrrol-2-yl)pyrimidin-2-amine* (**3r**): 123 mg (91%), light yellow crystals, m.p. 124–125 °C; ^1^H-NMR (400.13 MHz, CDCl_3_) δ: 8.03–8.01 (m, 2H, Ph), 7.62 (dd, *J* = 8.5, 15.8 Hz, 1H, H_X_), 7.50–7.46 (m, 5H, Ph), 7.42–7.38 (m, 2H, Ph), 7.34–7.31 (m, 1H, Ph), 7.30 (s, 1H, H-5, pyrimidine), 6.89 (d, *J* = 3.7 Hz, 1H, H-3, pyrrole), 6.38 (d, *J* = 3.7 Hz, 1H, H-4, pyrrole), 5.10 (br s, 2H, NH_2_), 4.96 (d, *J* = 8.5 Hz, 1H, H_A_), 4.68 (d, *J* = 15.8 Hz, 1H, H_B_); ^13^C-NMR (100.6 MHz, CDCl_3_) δ: 165.3, 163.1, 160.2, 138.9, 137.8, 133.0, 132.8, 132.7, 130.3, 129.3 (2C), 128.8 (2C), 128.2 (2C), 127.3, 127.0 (2C), 114.6, 111.8, 111.2, 105.8; IR (KBr) ν: 3463, 3407, 3312, 3187, 3060, 2953, 2922, 2854, 1622, 1566, 1531, 1451, 1429, 1389, 1376, 1343, 1291, 1220, 1076, 1026, 963, 900, 837, 761, 695, 640. Anal. Calcd. for C_22_H_18_N_4_: C, 78.08%; H, 5.36%; N, 16.56%. Found: C, 78.14%; H, 5.31%; N, 16.32%.

*4-(4,5-diphenyl-1-vinyl-1H-pyrrol-2-yl)-6-phenylpyrimidin-2-amine* (**3s**): 146 mg (88%), yellow crystals, m.p. 202 °C; ^1^H-NMR (400.13 MHz, CDCl_3_) δ: 8.06–8.03 (m, 2H, H, Ph), 7.51–7.49 (m, 3H, Ph, H_X_), 7.40–7.32 (m, 7H, Ph), 7.21–7.13 (m, 5H, Ph, H-5, pyrimidine), 7.06 (s, 1H, H-3, pyrrole), 5.10 (br s, 2H, NH_2_), 4.89 (d, *J* = 8.2 Hz, 1H, H_A_), 4.60 (d, *J* = 16.4 Hz, 1H, H_B_); ^13^C-NMR (100.6 MHz, CDCl_3_) δ: 165.4, 163.1, 160.2, 137.8, 135.3, 137.8, 132.4, 132.3, 131.8, 131.4 (2C), 130.4, 128.8 (2C), 128.5 (2C), 128.2 (2C), 128.1 (2C), 128.0, 127.1 (2C), 125.9, 125.0, 114.9, 110.8, 106.1. IR (KBr) ν: 3418, 3059, 2363, 1637, 1588, 1567, 1535, 1498, 1449, 1408, 1291, 1206, 1075, 955, 910, 832, 770, 696, 638. Anal. Calcd. for C_28_H_22_N_4_: C, 81.13%; H, 5.35%; N, 13.52%. Found: C, 78.99%; H, 5.16%; N, 13.37%.

*4-[5-(4-Chlorophenyl)-1-vinyl-1H-pyrrol-2-yl]-6-phenylpyrimidin-2-amine* (**3t**): 78 mg (52%), light brown crystals, m.p. 156 °C; ^1^H-NMR (400.13 MHz, CDCl_3_) δ: 8.04–8.01 (m, 2H, H-2,6, Ph), 7.63 (dd, *J* = 8.5, 15.8 Hz, 1H, H_X_), 7.51–7.49 (m, 3H, H-3,4,5, Ph), 7.41–7.35 (m, 4H, 4-Cl-C_6_H_4_), 7.29 (s, 1H, H-5, pyrimidine), 6.88 (d, *J* = 3.8 Hz, 1H, H-3, pyrrole), 6.36 (d, *J* = 3.8 Hz, 1H, H-4, pyrrole), 5.16 (br s, 2H, NH_2_), 5.99 (d, *J* = 8.5 Hz, 1H, H_A_), 4.67 (d, *J* = 15.8 Hz, 1H, H_B_); ^13^C-NMR (100.6 MHz, CDCl_3_) δ: 165.5, 163.0, 160.1, 137.8, 137.5, 133.3, 133.2, 132.7, 131.5, 130.6 (2C), 130.5, 128.8 (2C), 128.5 (2C), 127.1 (2C), 114.5, 112.2, 111.7, 105.8; IR (KBr) ν: 3499, 3441, 3313, 3189, 2362, 1617, 1565, 1532, 1458, 1434, 1384, 1224, 1083, 1010, 957, 896, 831, 766, 700, 642, 580, 521. Anal. Calcd. for C_22_H_17_ClN_4_: C, 70.87%; H, 4.60%; Cl, 9.51%; N, 15.03%. Found: C, 70.77%; H, 4.38%; Cl, 9.51%; N, 15.02%.

*4-[5-(2-Fluorophenyl)-1-vinyl-1H-pyrrol-2-yl]-6-(2-furyl)pyrimidin-2-amine* (**3u**): 104 mg (75%), orange crystals, m.p. 169 °C; ^1^H-NMR (400.13 MHz, CDCl_3_) δ: 7.63–7.56 (m, 2H, H_X_, H-5, furyl), 7.44–7.40 (m, 1H, H-6, 2-F-C_6_H_4_), 7.37–7.32 (m, 1H, H-4, 2-F-C_6_H_4_), 7.25 (s, 1H, H-5, pyrimidine), 7.21–7.09 (m, 3H, H-3,5, 2-F-C_6_H_4_, H-3, furyl), 6.94 (d, *J* = 3.8 Hz, 1H, H-3, pyrrole), 6.56 (dd, *J* = 1.8, 3.4 Hz, 1H, H-4, furyl), 6.39 (d, *J* = 3.8 Hz, 1H, H-4, pyrrole), 5.19 (br s, 2H, NH_2_), 4.87 (d, *J* = 8.5 Hz, 1H, H_A_), 4.62 (d, *J* = 15.8 Hz, 1H, H_B_); ^13^C-NMR (100.6 MHz, CDCl_3_) δ: 162.9, 160.0, 159.4 (d, ^1^*J*_CF_ = 248.3 Hz, C-2, 2-F-C_6_H_4_), 156.2, 152.3, 144,4, 133.2, 132.5 (d, ^3^*J*_CF_ = 5.9 Hz, C-4, 2-F-C_6_H_4_), 131.9, 131.8, 129.7 (d, ^3^*J*_CF_ = 8.1 Hz, C-6, 2-F-C_6_H_4_), 124.1 (d, ^4^*J*_CF_ = 3.4 Hz, C-5, 2-F-C_6_H_4_), 121.4 (d, ^2^*J*_CF_ = 14.9 Hz, C-1, 2-F-C_6_H_4_), 115.9 (d, ^2^*J*_CF_ = 22.0 Hz, C-3, 2-F-C_6_H_4_), 114.3, 112.7, 112.2, 111.3, 109.7, 103.3; IR (KBr) ν: 3433, 3363, 3195, 1631, 1600, 1550, 1459, 1408, 1222, 1160, 1108, 1079, 1017, 945, 817, 771, 750. Anal. Calcd. for C_20_H_15_FN_4_O: C, 69.35%; H, 4.37%; F, 5.49%; N, 16.18%. Found: C, 69.27%; H, 4.15%; F, 5.36%; N, 16.11%.

*4-[5-(2-Fluorophenyl)-1-vinyl-1H-pyrrol-2-yl]-6-(2-thienyl)pyrimidin-2-amine (***3v**): 99 mg (68%), brown crystals, m.p. 144 °C; ^1^H-NMR (400.13 MHz, CDCl_3_) δ: 7.73–7.72 (m, 1H, H-6, 2-F-C_6_H_4_), 7.57 (dd, *J* = 8.5, 15.7 Hz, 1H, Hx), 7.48–7.47 (m, 1H, H-3, thienyl), 7.43–7.40 (m, 1H, H-5, thienyl), 7.38–7.33 (m, 1H, H-4, 2-F-C_6_H_4_),7.22 (s, 1H, H-5, pyrimidine), 7.19–7.09 (m, 3H, H-3,5, 2-F-C_6_H_4_, H-4, thienyl), 6.91 (d, *J* = 3.7 Hz, 1H, H-3, pyrrole), 6.39 (d, *J* = 3.7 Hz, 1H, H-4, pyrrole), 5.08 (br s, 2H, NH_2_), 4.89 (d, *J* = 8.5 Hz, 1H, H_A_), 4.65 (d, *J* = 15.7 Hz, 1H, H_B_); ^13^C-NMR (100.6 MHz, CDCl_3_) δ: 162.9, 159.9, 159.8, 159.5 (d, ^1^*J*_CF_ = 248.4 Hz, C-2, 2-F-C_6_H_4_), 143.2, 133.2, 132.5 (d, ^2^*J*_CF_ = 21.5 Hz, C-1, 2-F-C_6_H_4_), 132.0, 131.9, 129.8 (d, ^3^*J*_CF_ = 8.0 Hz, C-4, 2-F-C_6_H_4_), 129.0, 128.1, 126.7, 124.1 (d, ^4^*J*_CF_ = 2.8 Hz, C-5, 2-F-C_6_H_4_), 121.4 (d, ^3^*J*_CF_ = 15.0 Hz, C-6, 2-F-C_6_H_4_), 115.9 (d, ^2^*J*_CF_ = 22.0 Hz, C-3, 2-F-C_6_H_4_), 114.1, 112.7, 109.7, 103.8; IR (KBr) ν: 3404, 3340, 3223, 1638, 1567, 1531, 1448, 1431, 1376, 1347, 1223, 1105, 1073, 1045, 947, 898, 816, 766, 710. Anal. Calcd. for C_20_H_15_FN_4_S: C, 66.28%; H, 4.17%; F, 5.24%; N, 15.46%; S, 8.85%. Found: C, 65.93%; H, 4.02%; F, 5.48%; N, 15.11%; S, 8.79%.

*3-[2-(2-Amino-6-phenylpyrimidin-4-yl)-1H-pyrrol-1-yl]-1-phenyl-3-(1H-pyrrol-2-yl)prop-2-en-1-one* (**4a**): 11 mg (13%), yellow crystals, m.p. 137–139 °C; ^1^H-NMR (400.13 MHz, CDCl_3_) δ: 13.64 (br s, 1H, NH), 7.90–7.85 (m, 4H, H-2,6, COPh, H-2,6, Ph), 7.55–7.51 (m, 1H, H-4, COPh), 7.45–7.42 (m, 5H, H-3,5, COPh, H-3,4,5, Ph), 7.19 (s, 1H, H-5, pyrimidine), 7.16–7.15 (m, 2H, H-5, H-5′, pyrrole), 7.06 (dd, *J* = 1.7, 3.6 Hz, 1H, H-3, pyrrole), 6.76 (s, 1H, HC=), 6.42 (dd, *J* = 2.8, 3.6 Hz, 1H, H-4, pyrrole), 6.28 (dd, *J* = 2.2, 3.8 Hz, 1H, H-4′, pyrrole), 6.24–6.23 (m, 1H, H-3′, pyrrole), 4.88 (br s, 2H, NH_2_); ^13^C-NMR (100.6 MHz, CDCl_3_) δ: 189.9, 165.2, 162.9, 158.8, 146.3, 139.4, 137.7, 133.1, 132.8, 130.3, 129.9, 129.3, 128.7 (2C), 128.6 (2C), 128.3 (2C), 127.0 (2C), 124.5, 118.6, 114.3, 112.7, 111.9, 109.7, 104.3; ^15^N-NMR (40.5 MHz, CDCl_3_) δ: −304.9 (NH_2_), −220.9 (NH), −208.8 (N-pyrrole), −148.2 (N-1), −145.2 (N-3); IR (KBr) ν: 3480, 3401, 3356, 3120, 3106, 1623, 1577, 1526, 1438, 1353, 1320, 1297, 1220, 1184, 1128, 1088, 1036, 998, 931, 909, 881, 833, 768, 696, 641. Anal. Calcd. for C_27_H_21_N_5_O: C, 75.16%; H, 4.91%; N, 16.23%. Found: C, 75.04%; H, 4.77%; N, 16.14%.

*3-[2-Amino-4-phenyl-6-(1H-pyrrol-2-yl)pyrimidin-5-yl]-1-phenyl-3-(1H-pyrrol-2-yl)prop-2-en-1-one* (**5a**): 7 mg (8%), dark yellow crystals, m.p. 182 °C; ^1^H-NMR (400.13 MHz, CDCl_3_) δ: 9.96 (br s, 1H, NH’), 8.95 (br s, 1H, NH), 7.59–7.56 (m, 2H, H-2,6, COPh), 7.45–7.42 (m, 1H, H-4, COPh), 7.31–7.30 (m, 1H, H-4, Ph), 7.29 (s, 1H, HC=), 7.25–7.23 (m, 2H, H-3,5,COPh), 7.21–7.19 (m, 2H, H-2,6, Ph), 7.14–7.10 (m, 2H, H-3,5, Ph), 6.83–6.82 (m, 1H, H-5′, pyrrole), 6.78–6.77 (m, 1H, H-5, pyrrole), 6.72–6.71 (m, 1H, H-3, pyrrole), 6.66–6.65 (m, 1H, H-3′, pyrrole), 6.31–6.30 (m, 1H, H-4′, pyrrole), 6.09–6.06 (m, 1H, H-4, pyrrole), 5.12 (br s, 2H, NH_2_); ^13^C-NMR (100.6 MHz, DMSO-d_6_) δ: 187.7 (C=O), 164.9 (C-4, pyrimidine), 161.4 (C-2, pyrimidine), 154.6 (C-6, pyrimidine), 144.4 (C=), 139.6 (C-1, Ph), 139.1 (C-1, COPh), 132.3 (C-2′, pyrrole), 132.0 (C-4, COPh), 128.4 (C-2, pyrrole), 128.3 (C-3,5, COPh), 127.8 (C-2,4,6, Ph), 127.5 (C-2,6, COPh), 127.0 (C-3,5, Ph), 123.8 (C-5′, pyrrole), 120.7 (C-5, pyrrole), 114.9 (C-3′, pyrrole), 114.8 (C-5, pyrimidine, HC=), 112.5 (C-3, pyrrole), 110.3 (C-4′, pyrrole), 109.4 (C-4, pyrrole); ^15^N-NMR (40.5 MHz, CDCl_3_) δ: −225.5 (NH’), −223.7 (NH); IR (KBr) ν: 3471, 3426, 3173, 2967, 2863, 1635, 1597, 1536, 1444, 1418, 1227, 1120, 1034, 1020, 910, 738, 697. Anal. Calcd. for C_27_H_21_N_5_O: C, 75.16%; H, 4.91%; N, 16.23%. Found: C, 74.98%; H, 4.64%; N, 16.14%.

## 4. Conclusions

In summary, we developed an effective access to novel families of pyrrole–pyrimidine ensembles, decorated with alkyl, cycloalkyl, aryl and vinyl groups, attractive objects for drug design, by using acylethynylpyrroles as the synthetic platform. This method has several synthetic advantages, such as the one-pot procedure, the use of readily available starting materials and good to high yields of the target ensembles and therefore can activate the interest of both synthetic and pharmaceutical communities.

## Figures and Tables

**Table 1:** Effect of reaction conditions on the synthesis of pyrrole–aminopyrimidine ensemble **3l** from 2-benzoylethynyl-5-phenylpyrrole (**1l**) and guanidine nitrate **2**.

Entry	Base	Solvent	Ratio 1l/2/Base, mol	T, °C	Time, h	Content of 3l in the Reaction Mixture, % ^a^
1	Na_2_CO_3_	MeCN	1/1/1.5	82	4	11 ^b^
2	KOH	MeCN	1/1/1.5	82	4	68 ^c^
3	Et_3_N	*t*-BuOH	1/1/1.5	82	4	0
4	DBU	*t-*BuOH	1/1/1.5	82	4	0 ^d^
5	Cs_2_CO_3_	THF	1/2/1.5	66	4	0
6	Cs_2_CO_3_	THF	1/2/2	66	14	0
7	Cs_2_CO_3_	THF	1/2/4	66	18	15
8	Cs_2_CO_3_	THF	1/6/12	66	4	62
9	Cs_2_CO_3_	THF	1/10/20	66	4	68
10	Cs_2_CO_3_	THF	1/10/20	66	7	74
11	Cs_2_CO_3_	THF	1/10/20	66	12	89
12	Cs_2_CO_3_	THF	1/10/20	66	18	91
13	K_3_PO_4_	DMSO	1/5/6	20	16	0
14	K_3_PO_4_	DMSO	1/5/6	65–70	4	traces
15	K_3_PO_4_	DMSO	1/5/6	85–90	4	59 ^e^
16	K_3_PO_4_	DMSO	1/5/6	85–90	6	80 ^e^
17	KOH	DMSO	1/1/2	20	1	0
18	KOH	DMSO	1/2/4	70–75	13	93
19	KOH	DMSO	1/1/2	110–115	4	100 (77)
20	KOH	DMSO	1/1/1	110–115	4	95 (82)
21	KOH	DMSO	1/1.5/2	110–115	4	100 (88)
22	KOH	DMSO	1/1/1.5	110–115	4	100 (89)

^a^ The isolated yield of the product is indicated in parentheses; ^b^ conversion of pyrrole **1l** is 89%; ^c^ content of unidentified products is ~20%; ^d^ mixture of unidentified compounds; ^e^ strong tarring of the reaction mixture. THF: tetrahydrofuran; DMSO: dimethyl sulfoxide.

## Data Availability

Data set presented in this study is available in this article.

## Supplementary Materials

The following are available online, Figures S1–S68: ^1^H, ^13^C and ^19^F NMR spectra.

## Sample Availability

Samples of the compounds are available on request from the corresponding authors.
