# Supplementary material for: Pyrrole–Aminopyrimidine Ensembles: Cycloaddition of Guanidine to Acylethynylpyrroles
Source: Molecules. 2021 Mar 17;26(6):1692. doi: 10.3390/molecules26061692 (PMC8002744; doi:10.3390/molecules26061692)

# Pyrrole-Aminopyrimidine Ensembles: Cycloaddition of Guanidine to Acylethynylpyrroles

Olga V. Petrova, Arsalan B. Budaev, Elena F. Sagitova, Igor. A. Ushakov, Lyubov N. Sobenina, Andrey V. Ivanov and Boris A. Trofimov

A.E. Favorsky Irkutsk Institute of Chemistry, Siberian Branch, Russian Academy of Sciences, 1 Favorsky Str., 664033 Irkutsk, Russia

***** Correspondence: boris_trofimov@irioch.irk.ru; Tel.: +7-(3952)-41-93-46

Figure S1: $^{1}$H NMR spectrum ($CDCl_3$) of 3-(1-methyl-1*H*-pyrrol-2-yl)-1-phenylprop-2-yn-1-one **(1b)**

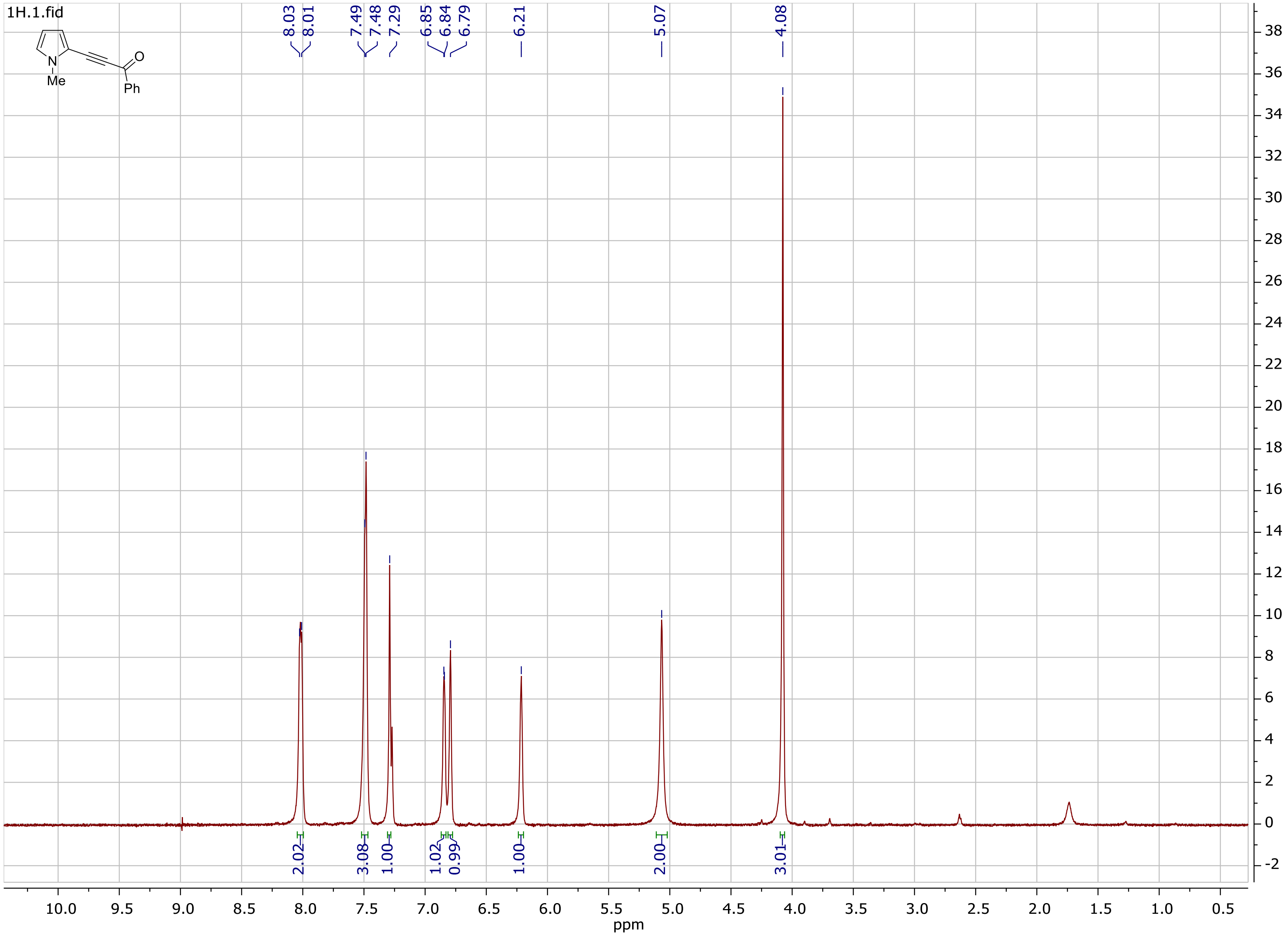

Figure S2: $^{13}$C NMR spectrum ($CDCl_3$) of 3-(1-methyl-1*H*-pyrrol-2-yl)-1-phenylprop-2-yn-1-one **(1b)**

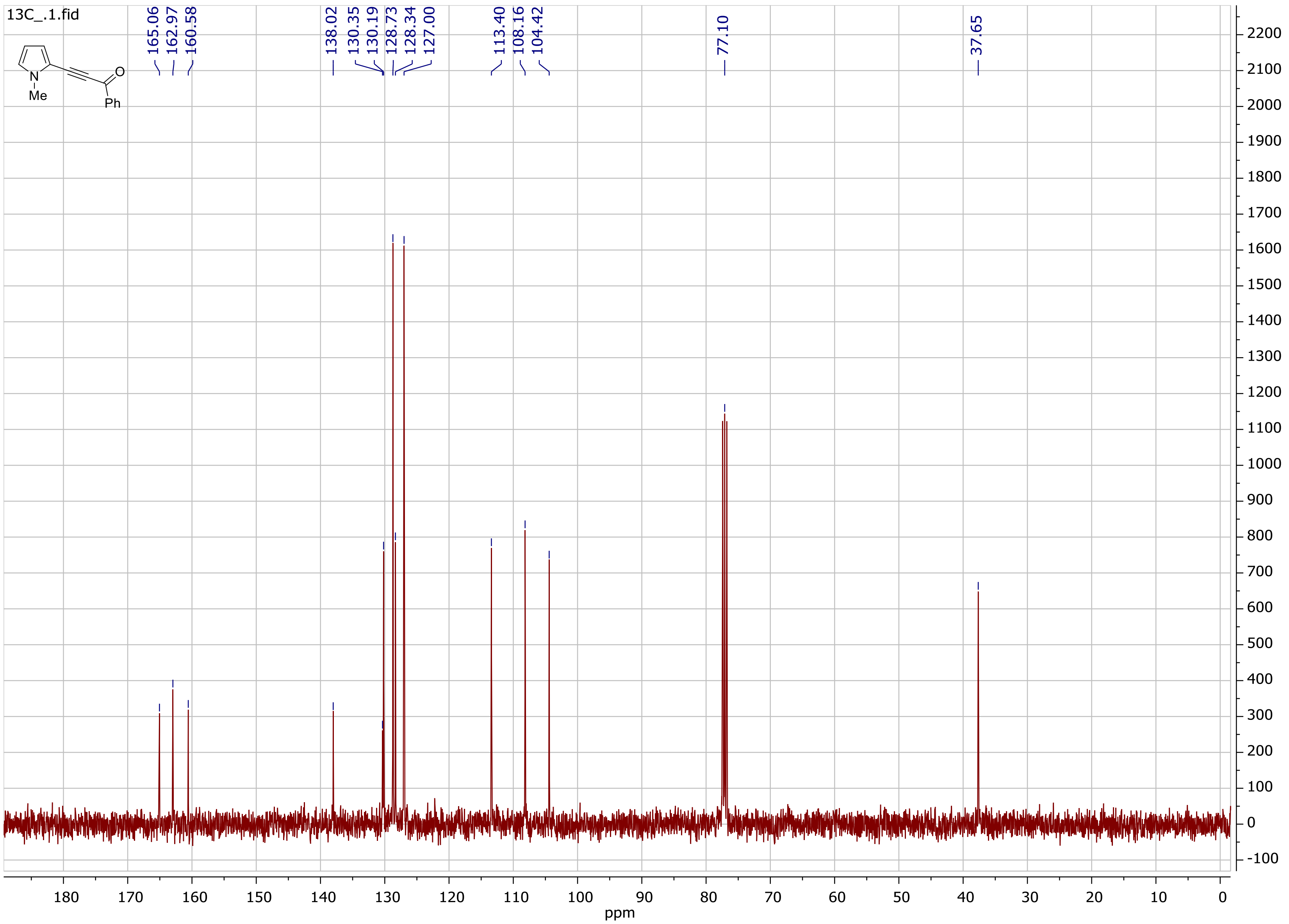

Figure S3: $^{1}$H NMR spectrum ($CDCl_3$) 3-(1-benzyl-1*H*-pyrrol-2-yl)-1-phenylprop-2-yn-1-one **(1c)**

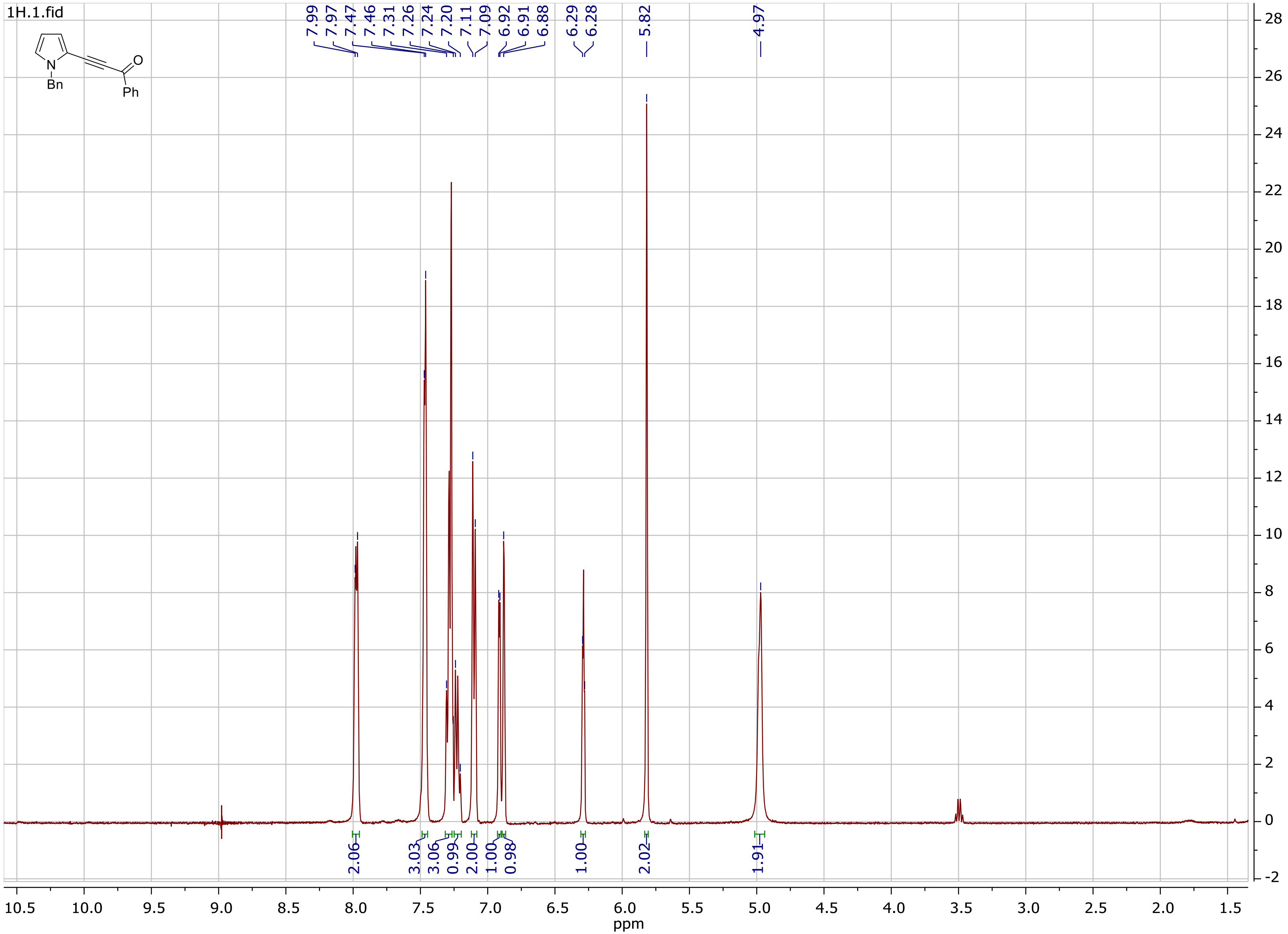

Figure S4: $^{13}$C NMR spectrum ($CDCl_3$) 3-(1-benzyl-1*H*-pyrrol-2-yl)-1-phenylprop-2-yn-1-one **(1c)**

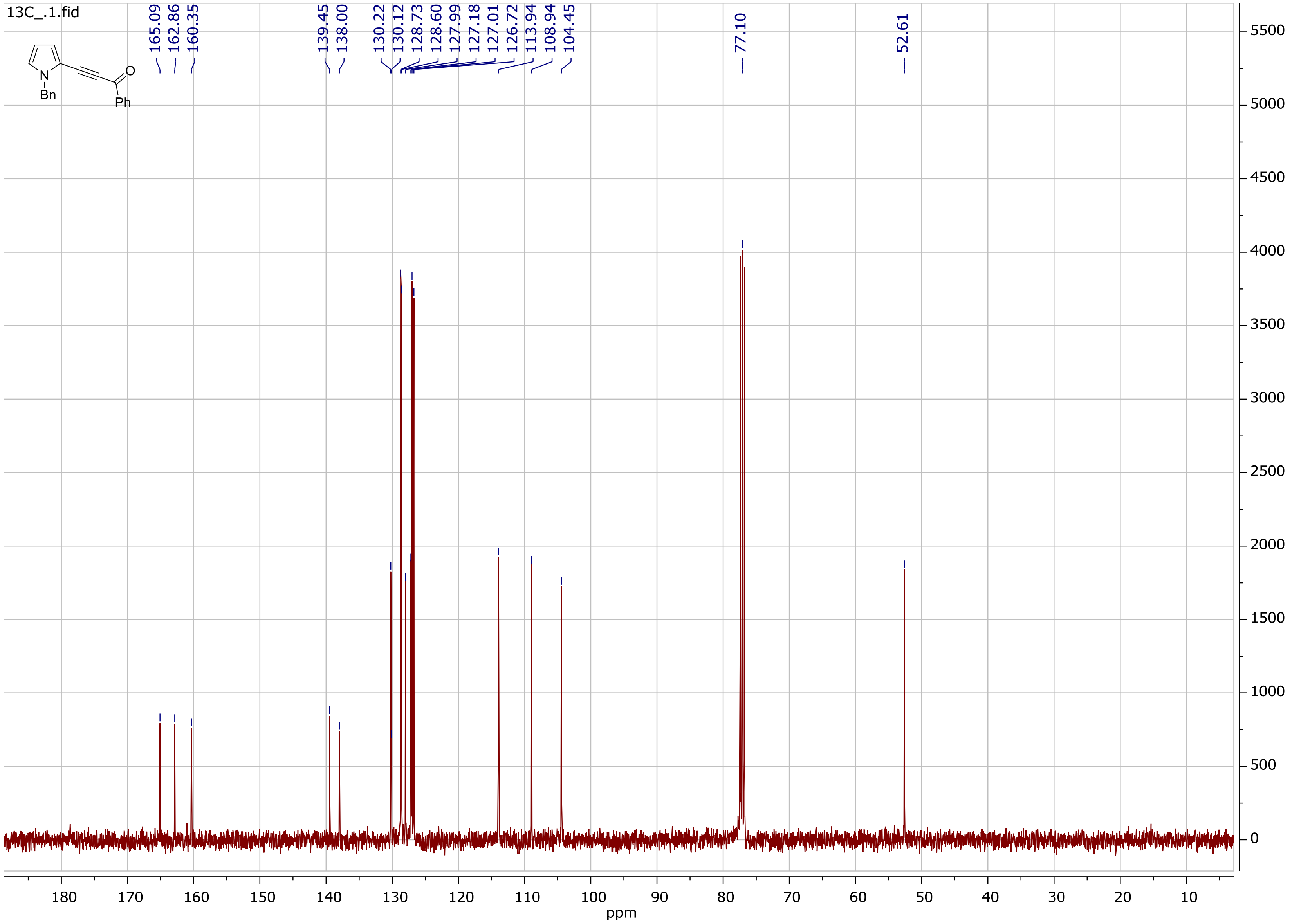

Figure S5: $^{1}$H NMR spectrum ($CDCl_3$) 3-(4-ethyl-5-propyl-1*H*-pyrrol-2-yl)-1-phenylprop-2-yn-1-one **(1d)**

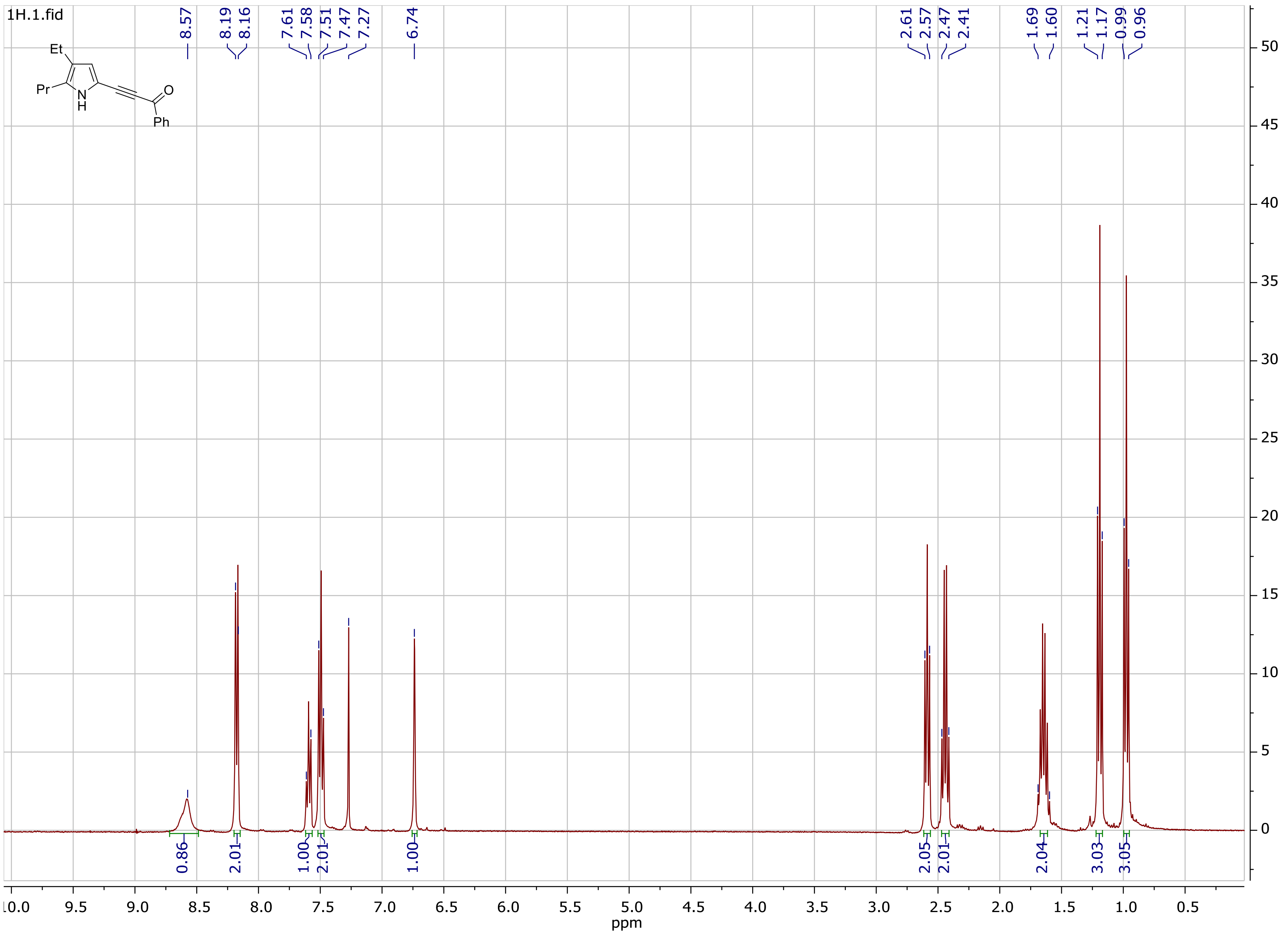

Figure S6: $^{1}$H NMR spectrum ($CDCl_3$) 3-(4-ethyl-5-propyl-1*H*-pyrrol-2-yl)-1-phenylprop-2-yn-1-one **(1d)**

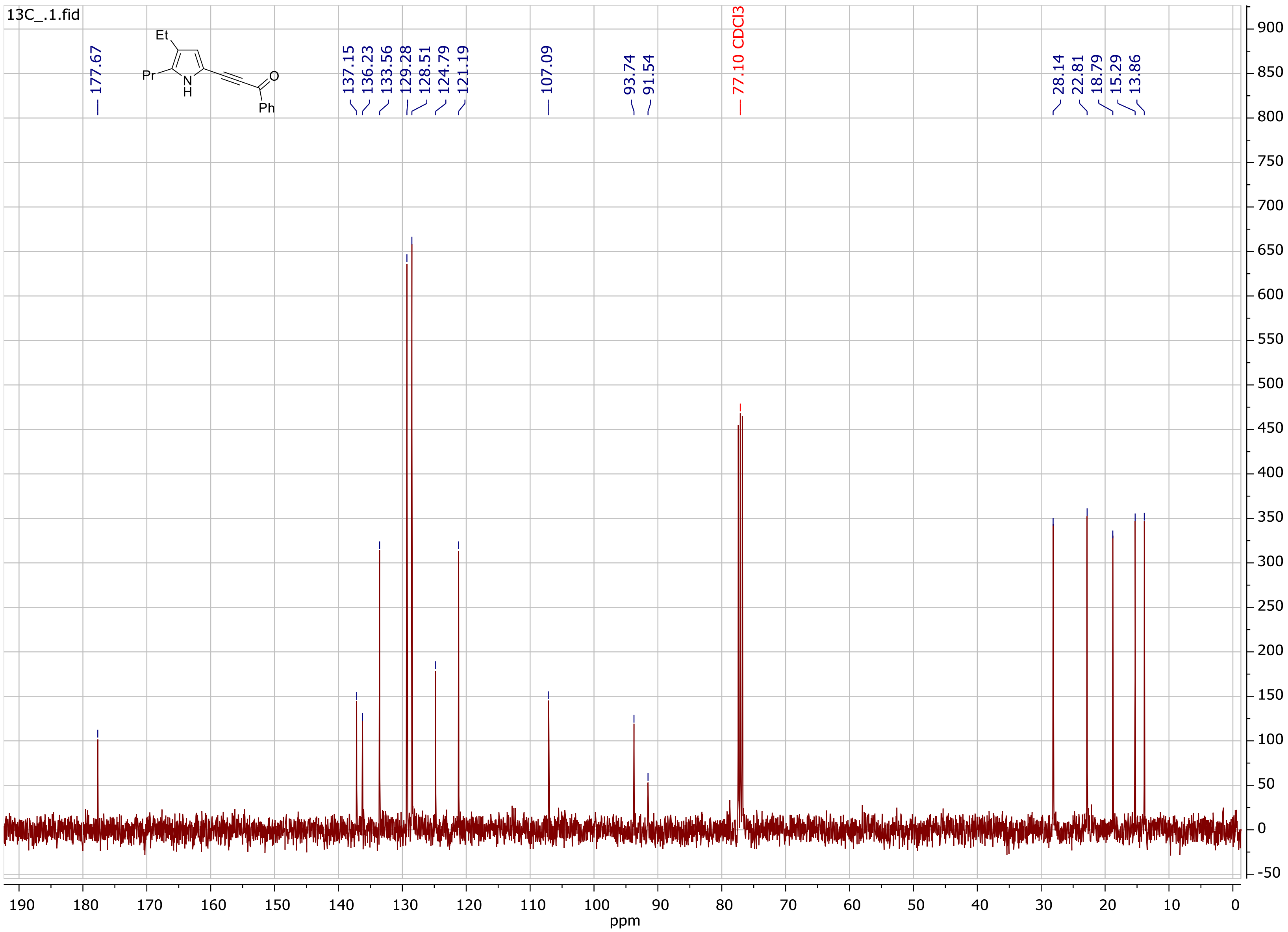

Figure S7: $^{1}$H NMR spectrum ($CDCl_3$) 3-(5-butyl-4-propyl-1*H*-pyrrol-2-yl)-1-phenylprop-2-yn-1-one **(1e)**

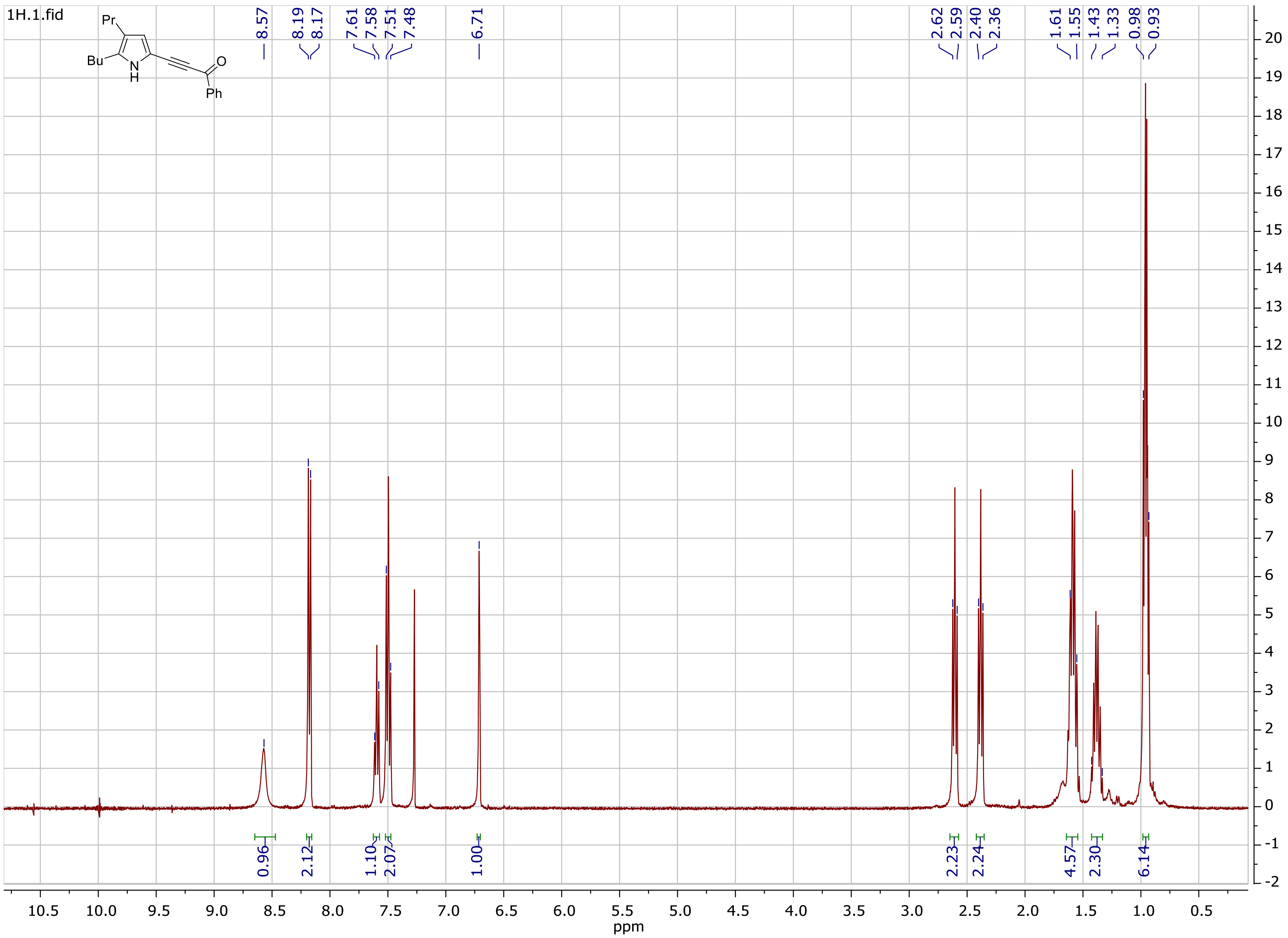

Figure S8: $^{13}C$ NMR spectrum ($CDCl_3$) 3-(5-butyl-4-propyl-1*H*-pyrrol-2-yl)-1-phenylprop-2-yn-1-one **(1e)**

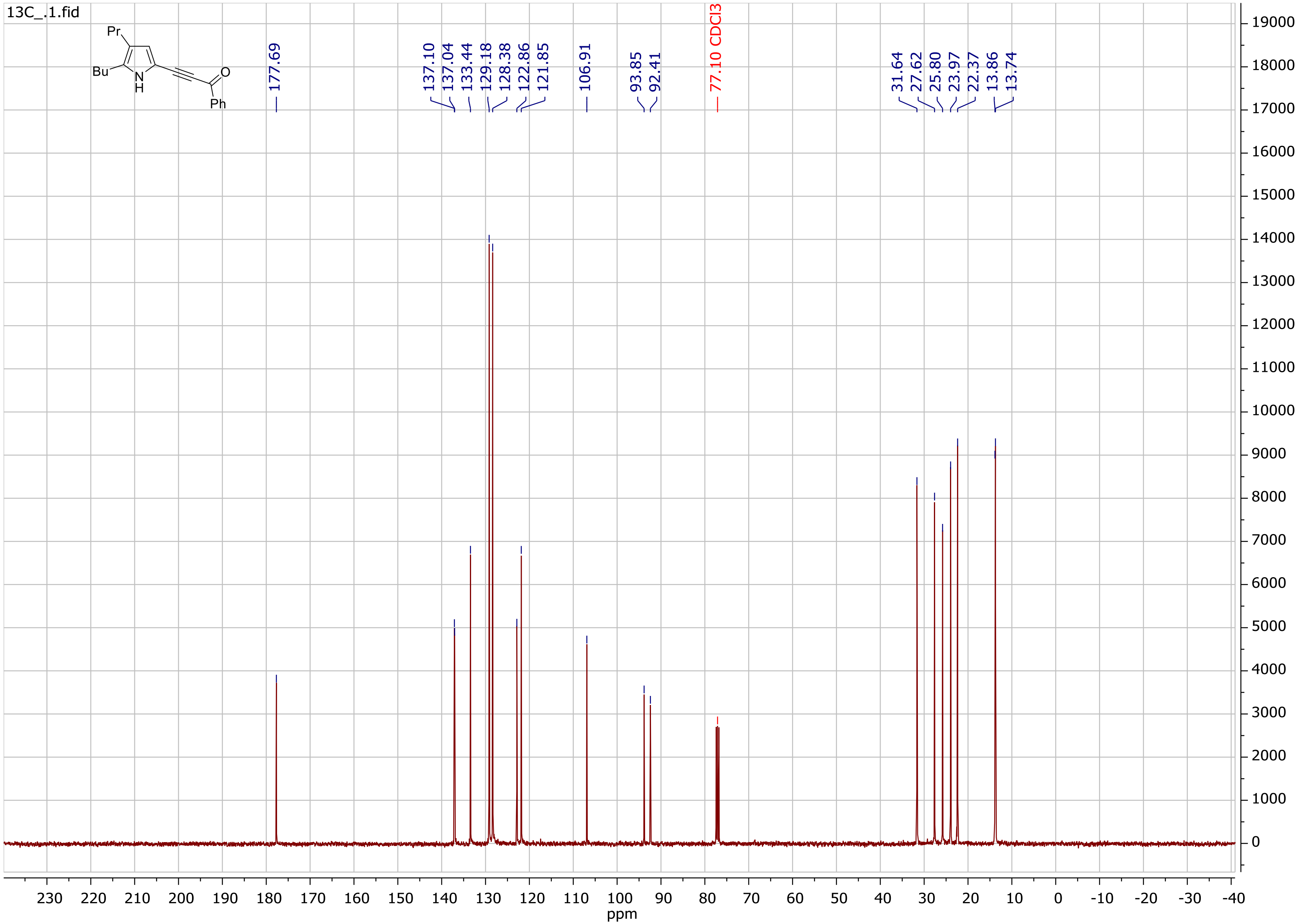

Figure S9: $^{1}$H NMR spectrum ($CDCl_3$) 1-(furan-2-yl)-3-(5-phenyl-1*H*-pyrrol-2-yl)prop-2-yn-1-one **(1m)**

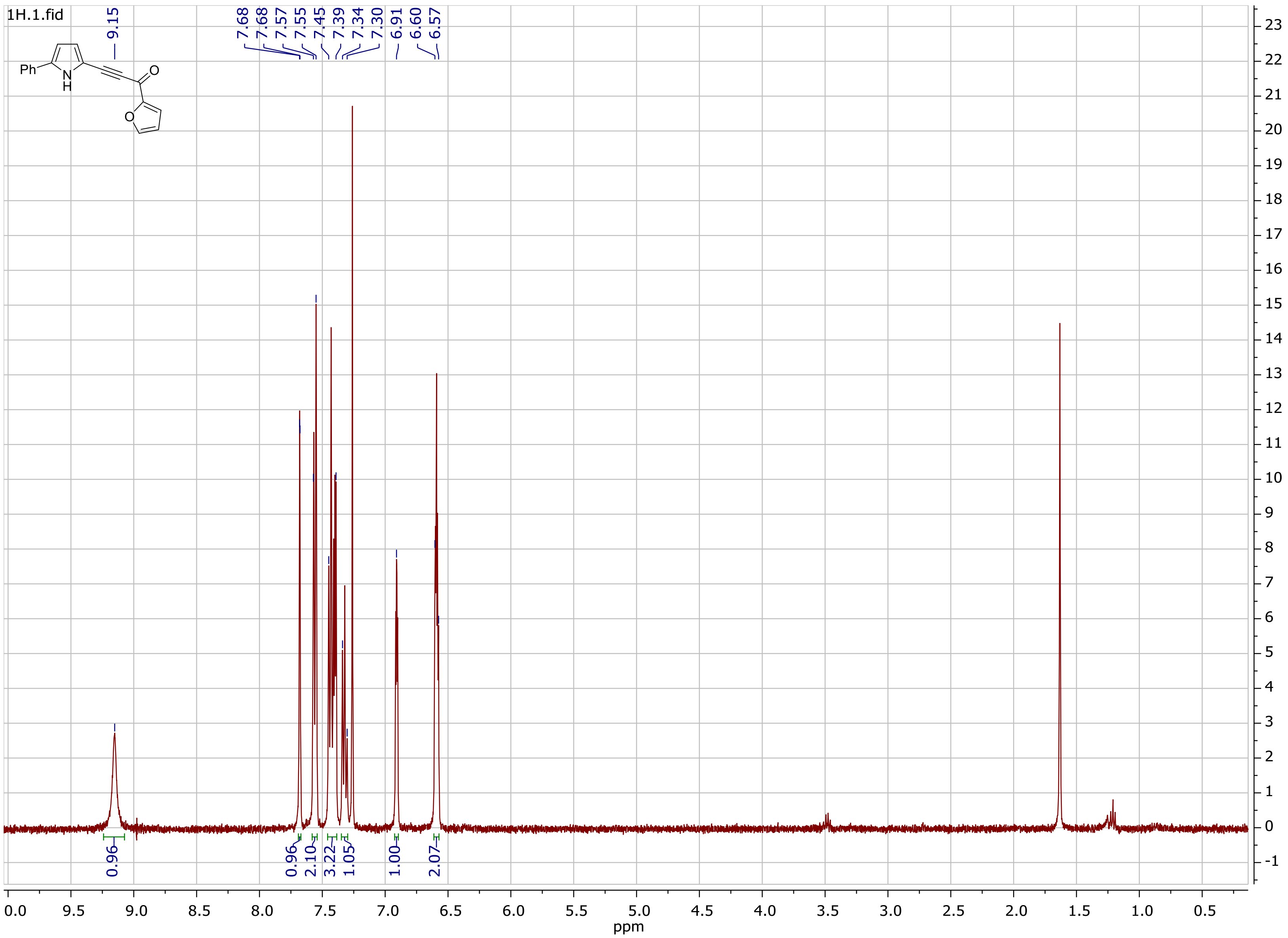

Figure S10: $^{13}$C NMR spectrum ($CDCl_3$) 1-(furan-2-yl)-3-(5-phenyl-1*H*-pyrrol-2-yl)prop-2-yn-1-one **(1m)**

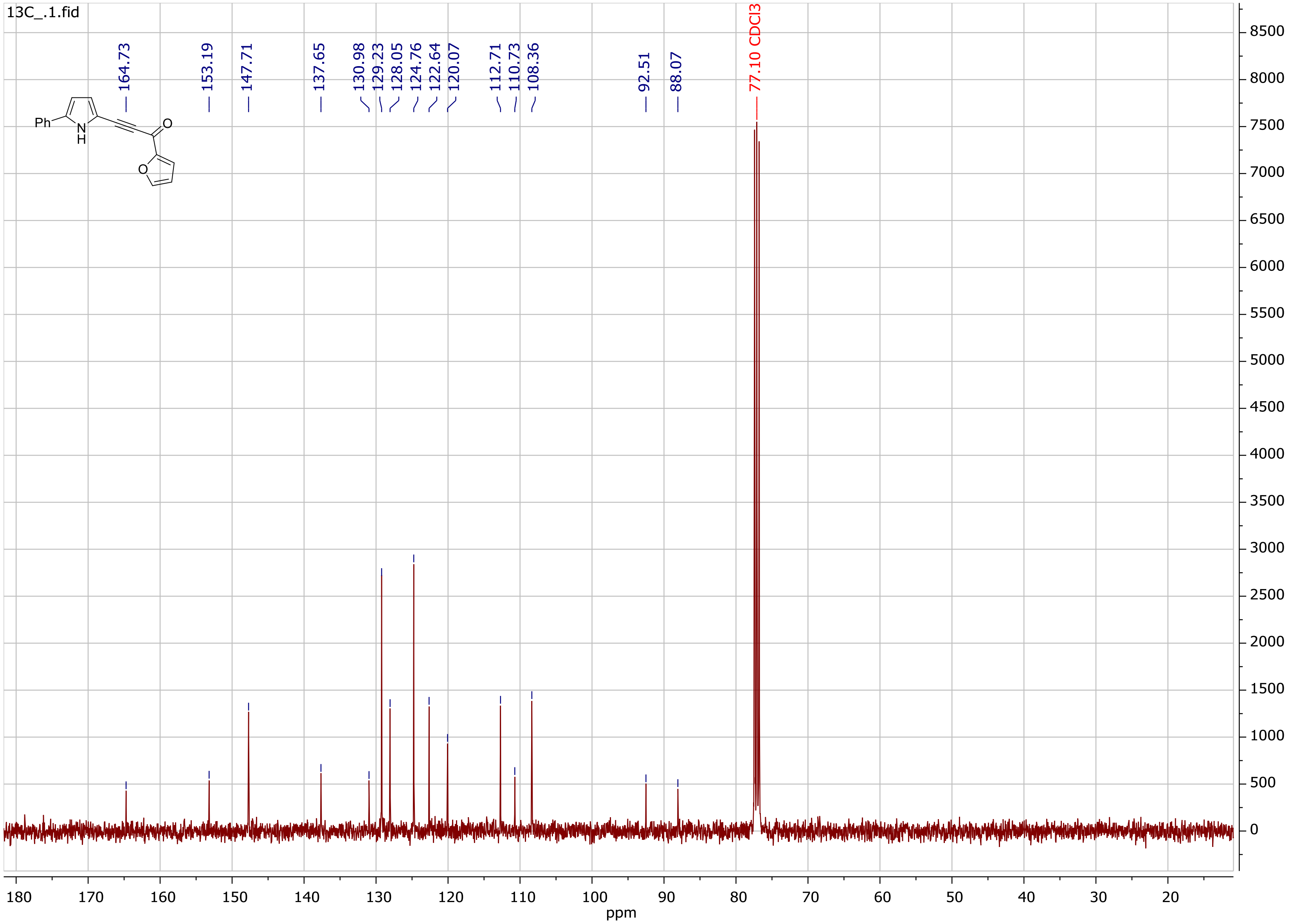

Figure S11: $^{1}$H NMR spectrum ($CDCl_3$) 3-(4,5-diphenyl-1-vinyl-1*H*-pyrrol-2-yl)-1-phenylprop-2-yn-1-one **(1s)**

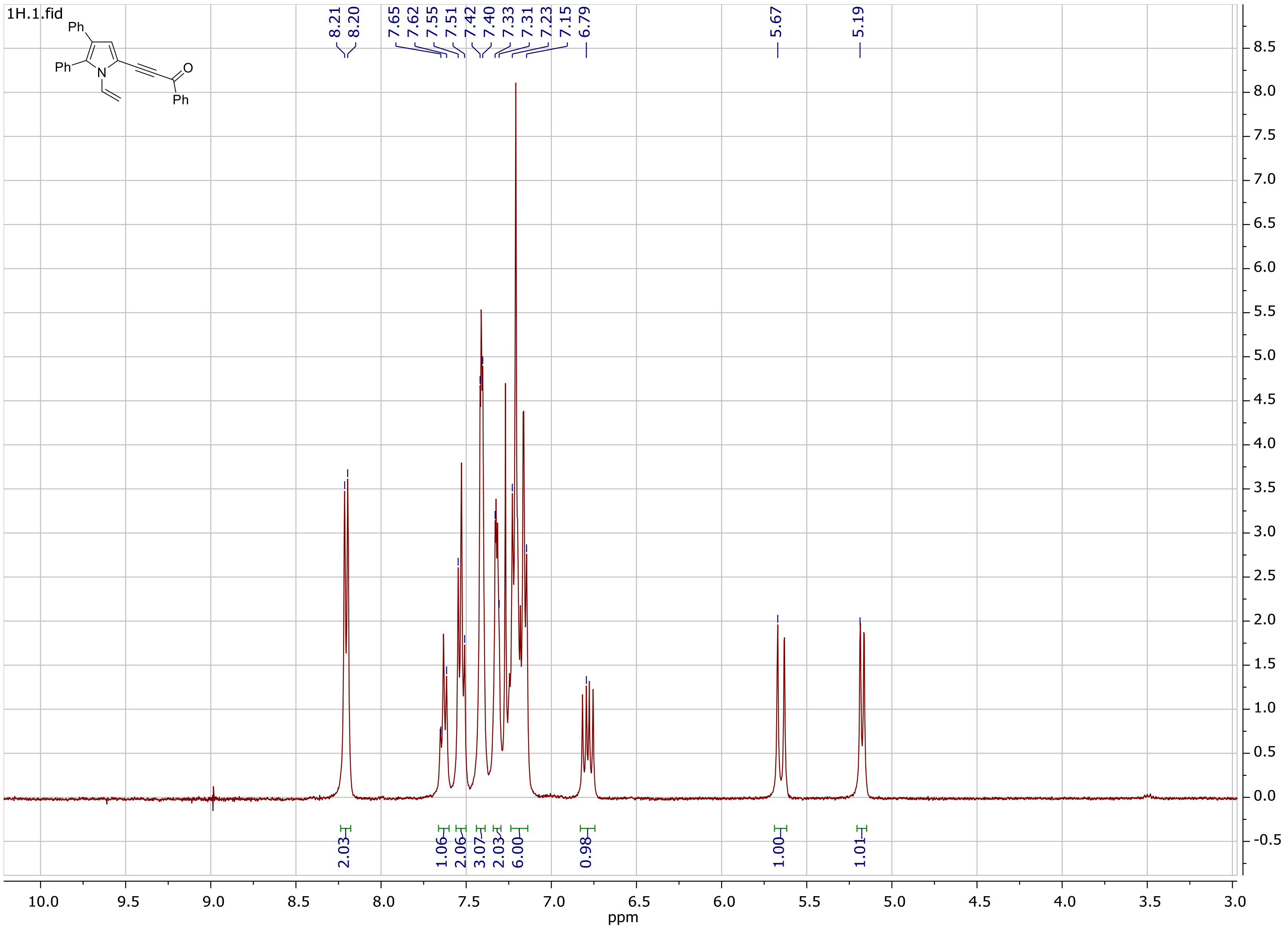

Figure S12: $^{13}$C NMR spectrum ($CDCl_3$) 3-(4,5-diphenyl-1-vinyl-1*H*-pyrrol-2-yl)-1-phenylprop-2-yn-1-one **(1s)**

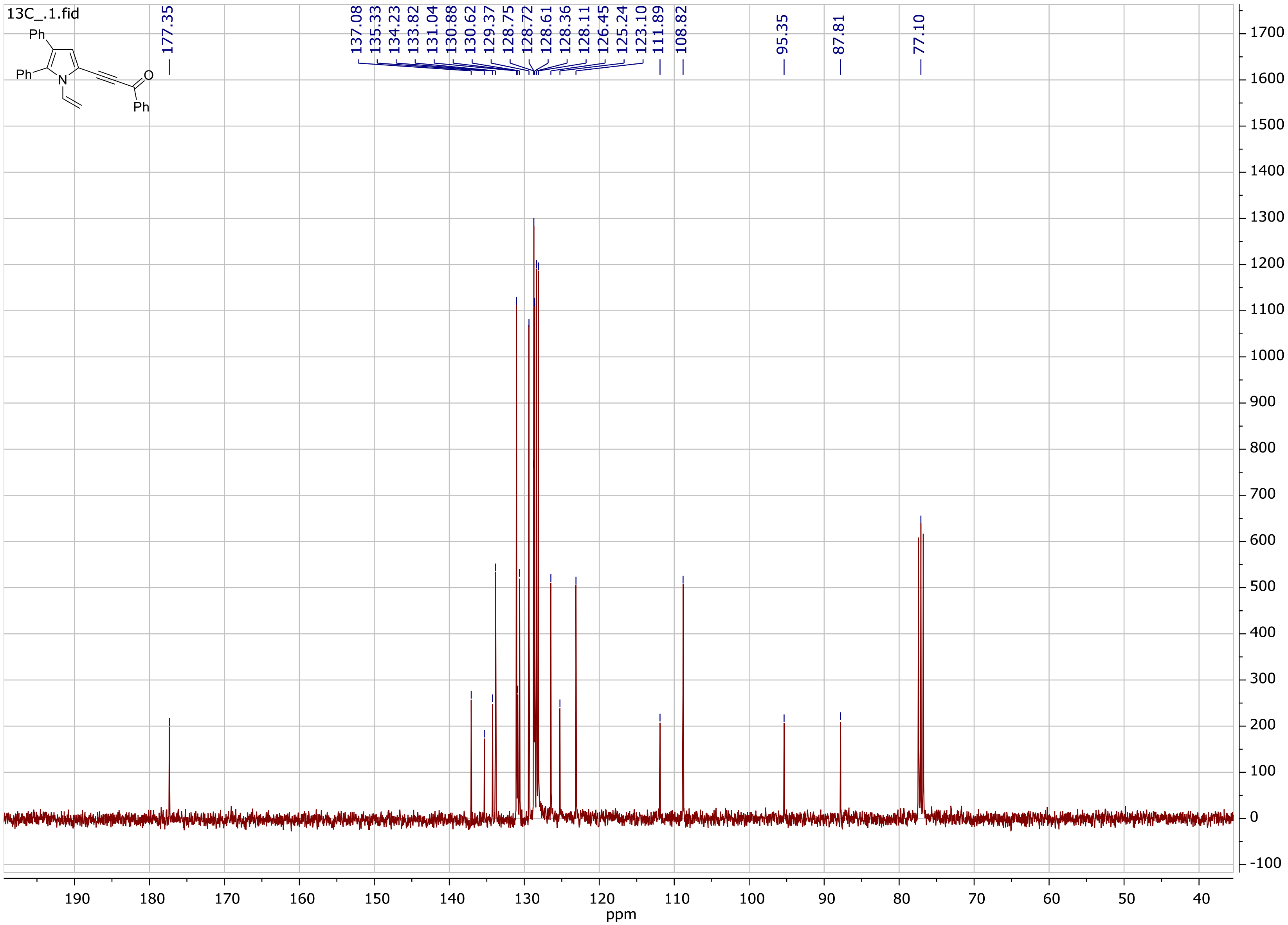

Figure S13: $^1$H NMR spectrum ($CDCl_3$) 3-(5-(4-chlorophenyl)-1-vinyl-1*H*-pyrrol-2-yl)-1-phenylprop-2-yn-1-one **(1t)**

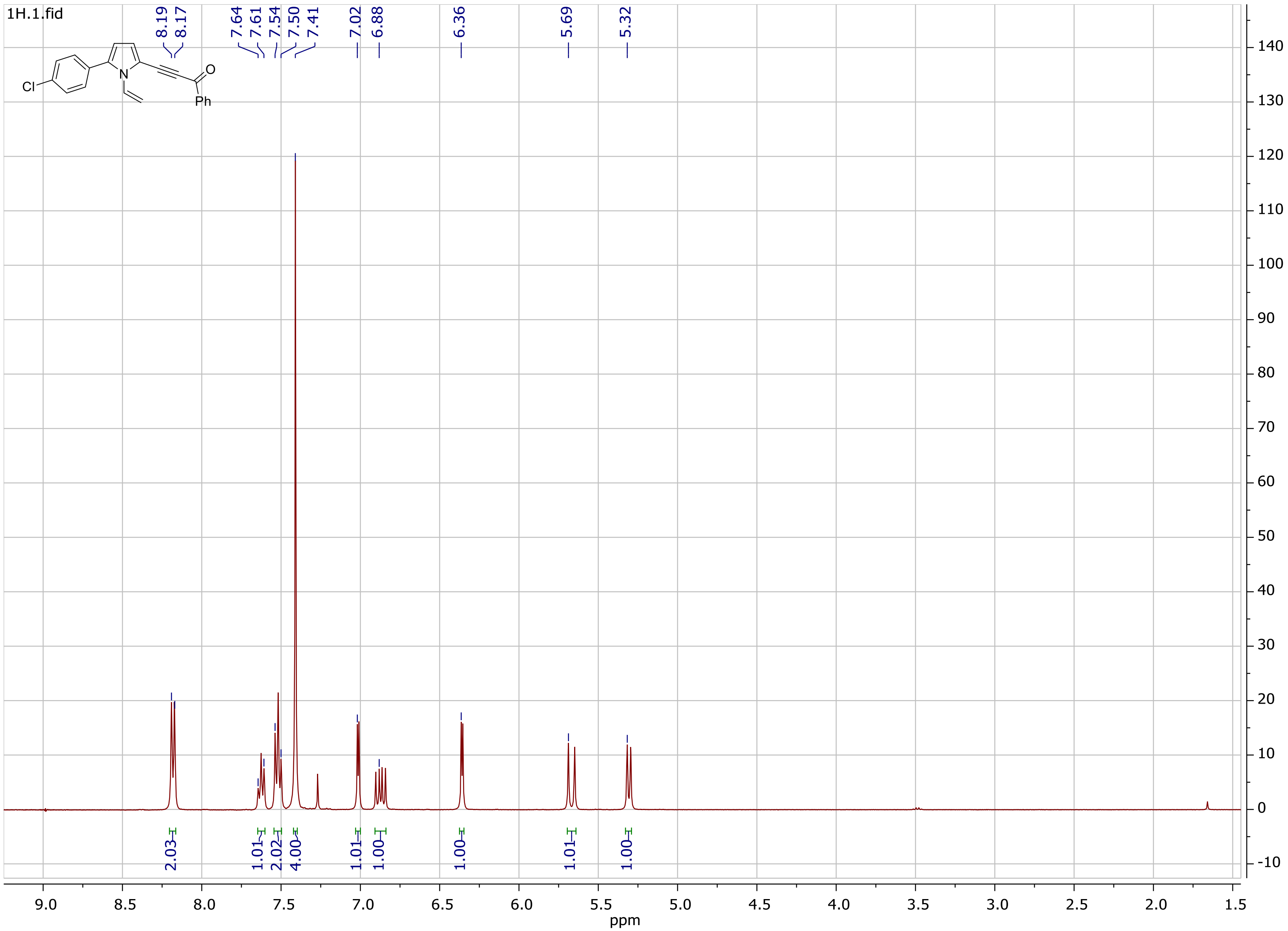

Figure S14: $^{13}$C NMR spectrum ($CDCl_3$) 3-(5-(4-chlorophenyl)-1-vinyl-1*H*-pyrrol-2-yl)-1-phenylprop-2-yn-1-one **(1t)**

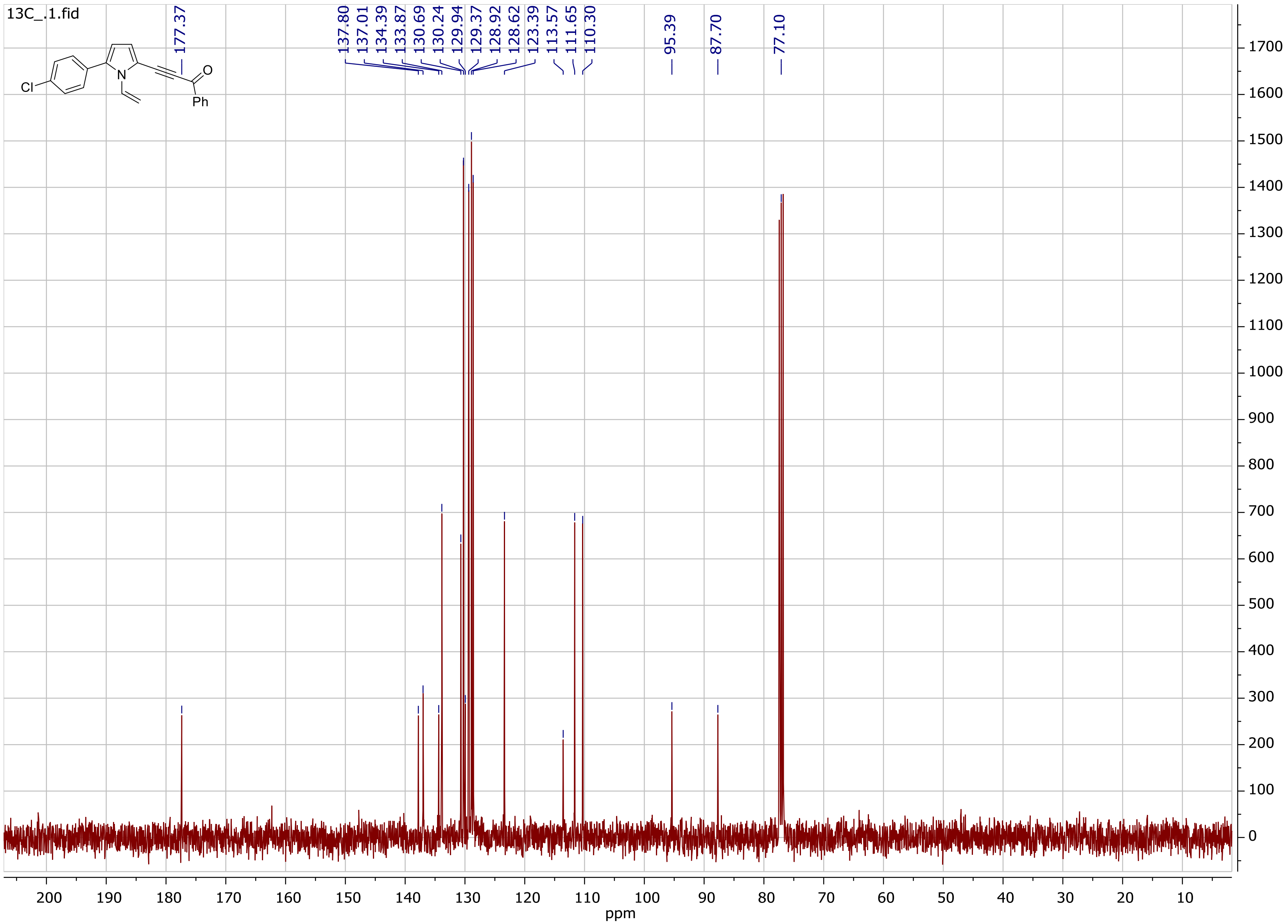

Figure S15: $^1$H NMR spectrum ($CDCl_3$) 3-(5-(2-fluorophenyl)-1-vinyl-1*H*-pyrrol-2-yl)-1-(furan-2-yl)prop-2-yn-1-one **(1u)**

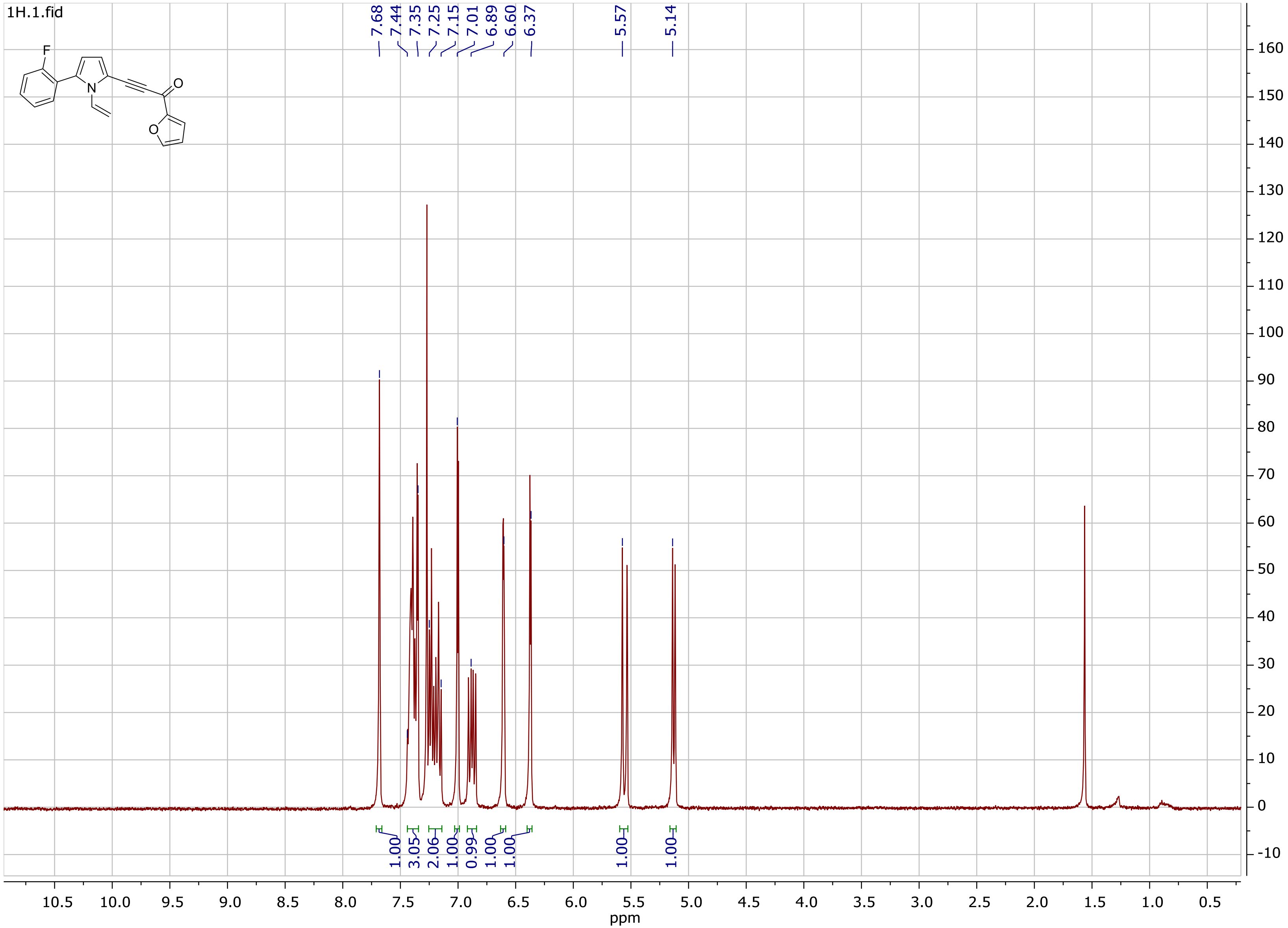

Figure S16: $^{13}$C NMR spectrum ($CDCl_3$) 3-(5-(2-fluorophenyl)-1-vinyl-1*H*-pyrrol-2-yl)-1-(furan-2-yl)prop-2-yn-1-one **(1u)**

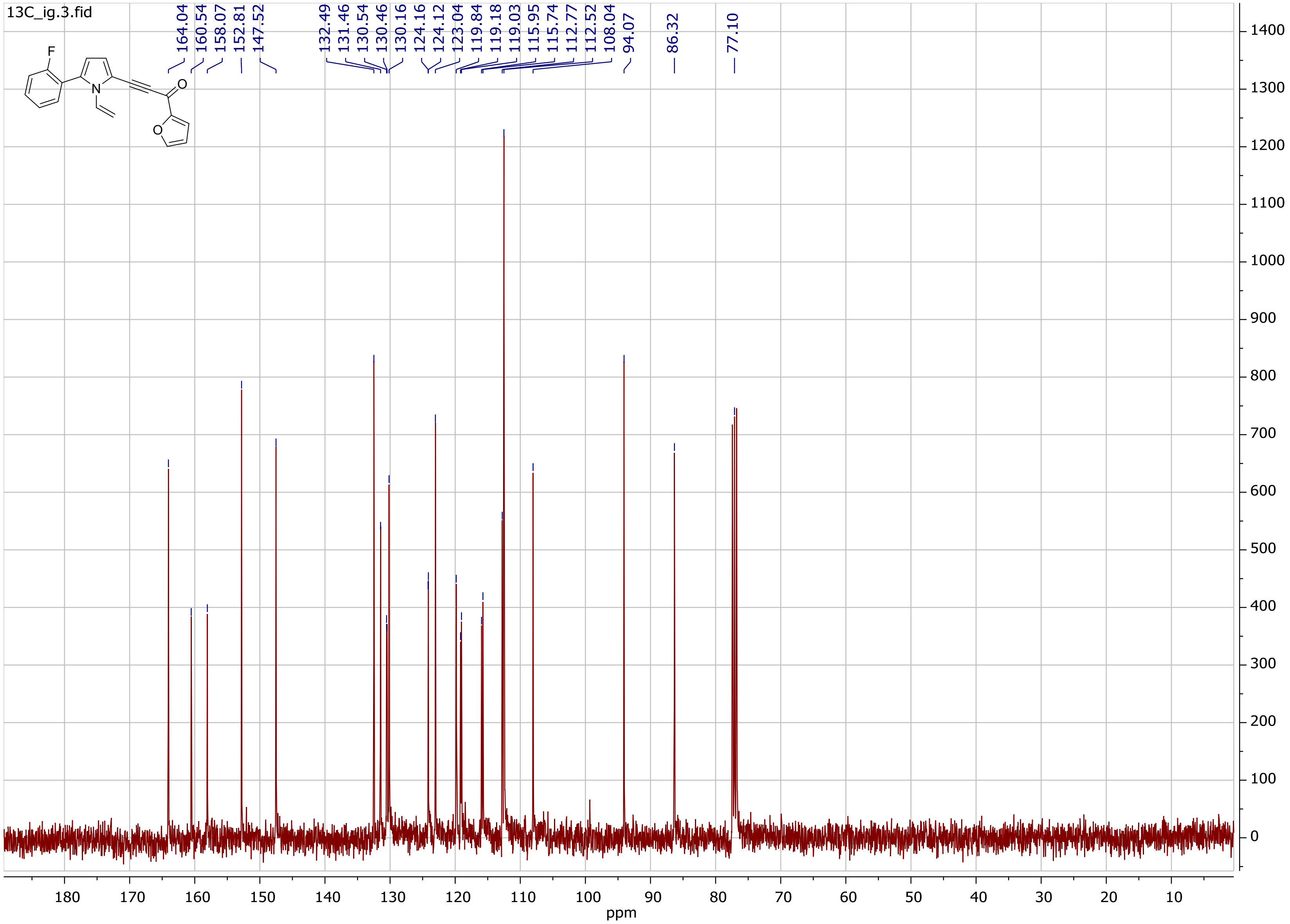

Figure S17: $^{19}$F NMR spectrum ($CDCl_3$) 3-(5-(2-fluorophenyl)-1-vinyl-1*H*-pyrrol-2-yl)-1-(furan-2-yl)prop-2-yn-1-one **(1u)**

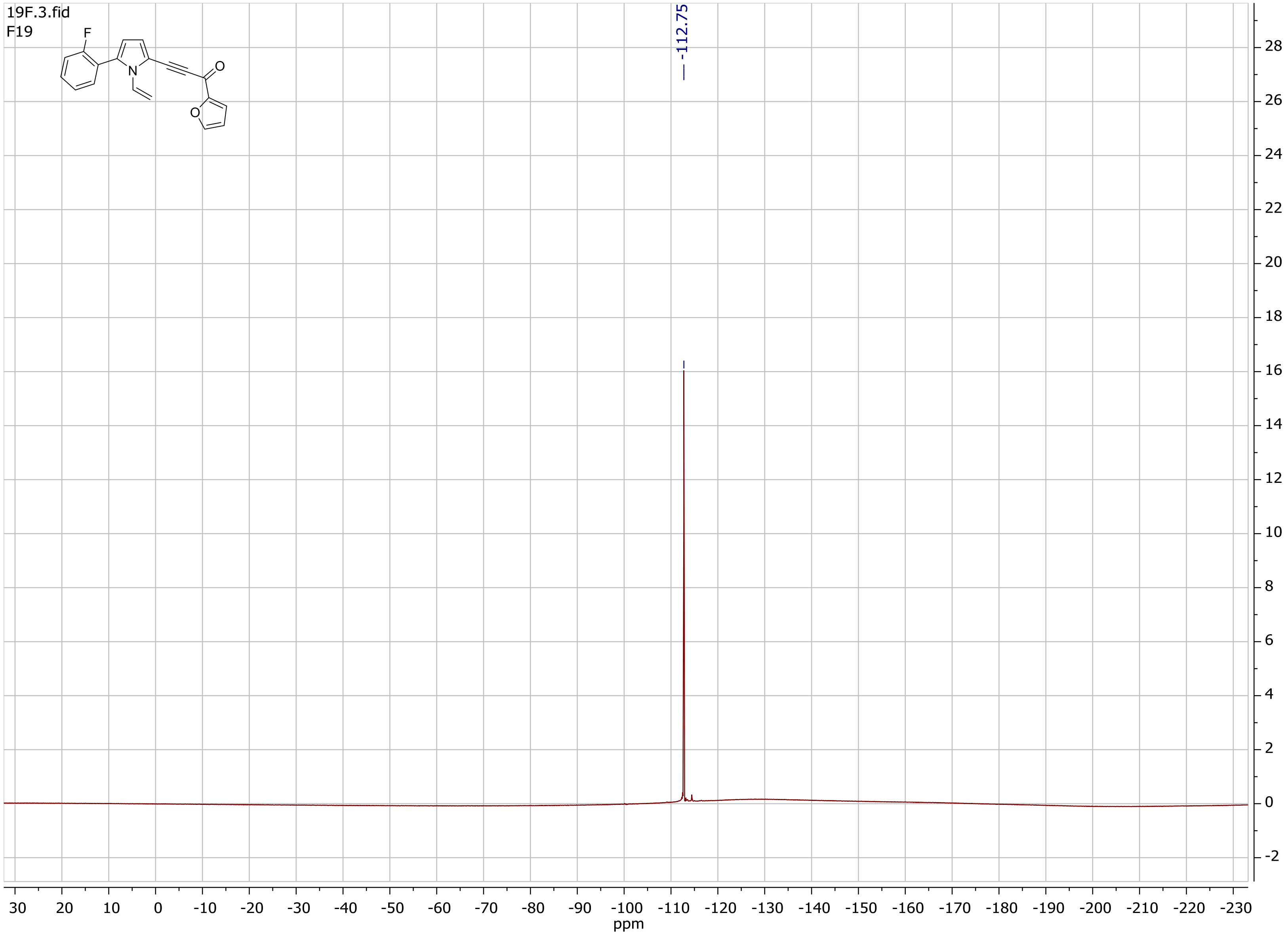

Figure S18: $^{1}$H NMR spectrum ($CDCl_3$) 3-(5-(2-fluorophenyl)-1-vinyl-1*H*-pyrrol-2-yl)-1-(thiophen-2-yl)prop-2-yn-1-one **(1v)**

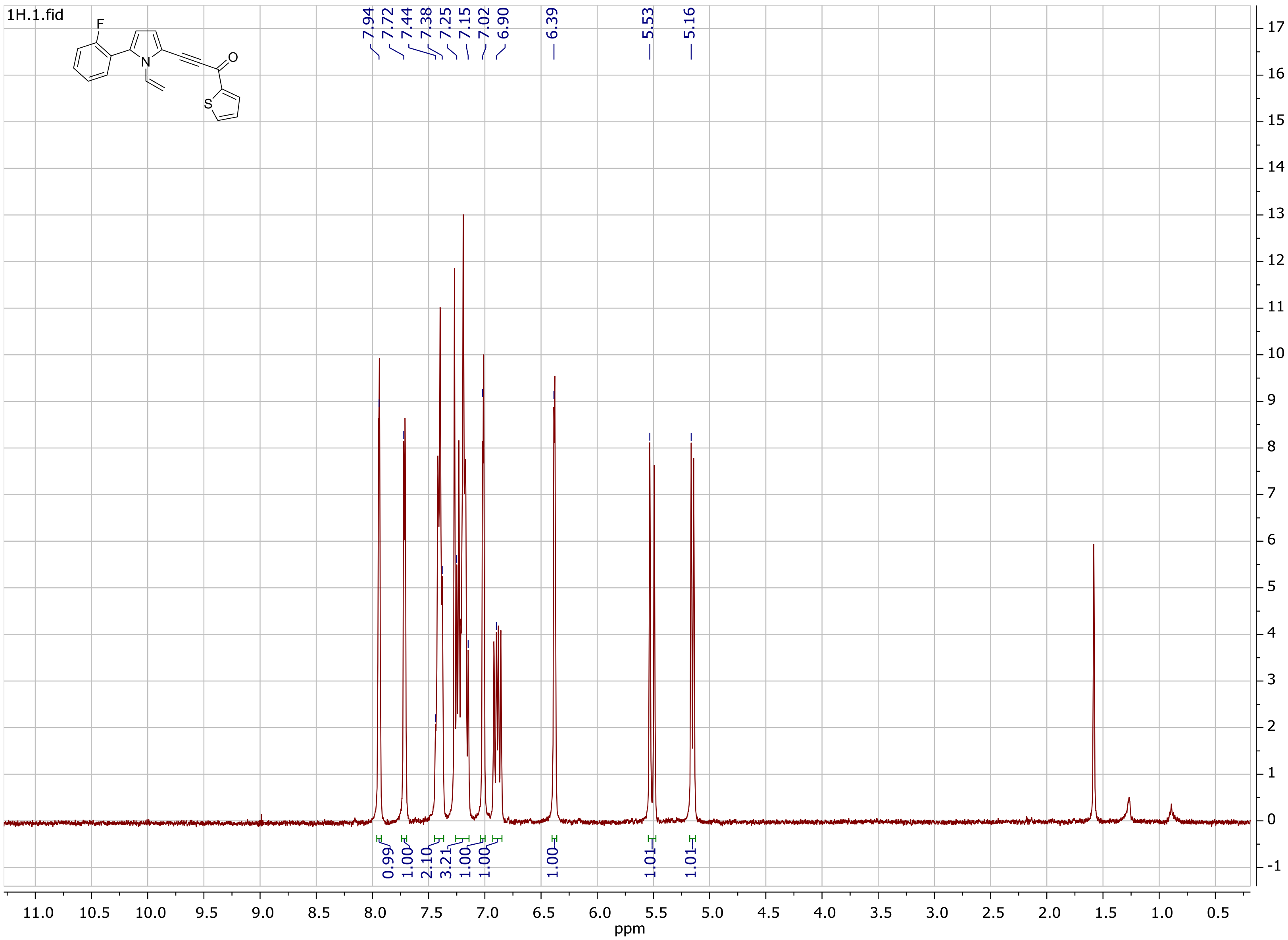

Figure S19: $^{13}$C NMR spectrum ($CDCl_3$) 3-(5-(2-fluorophenyl)-1-vinyl-1*H*-pyrrol-2-yl)-1-(thiophen-2-yl)prop-2-yn-1-one **(1v)**

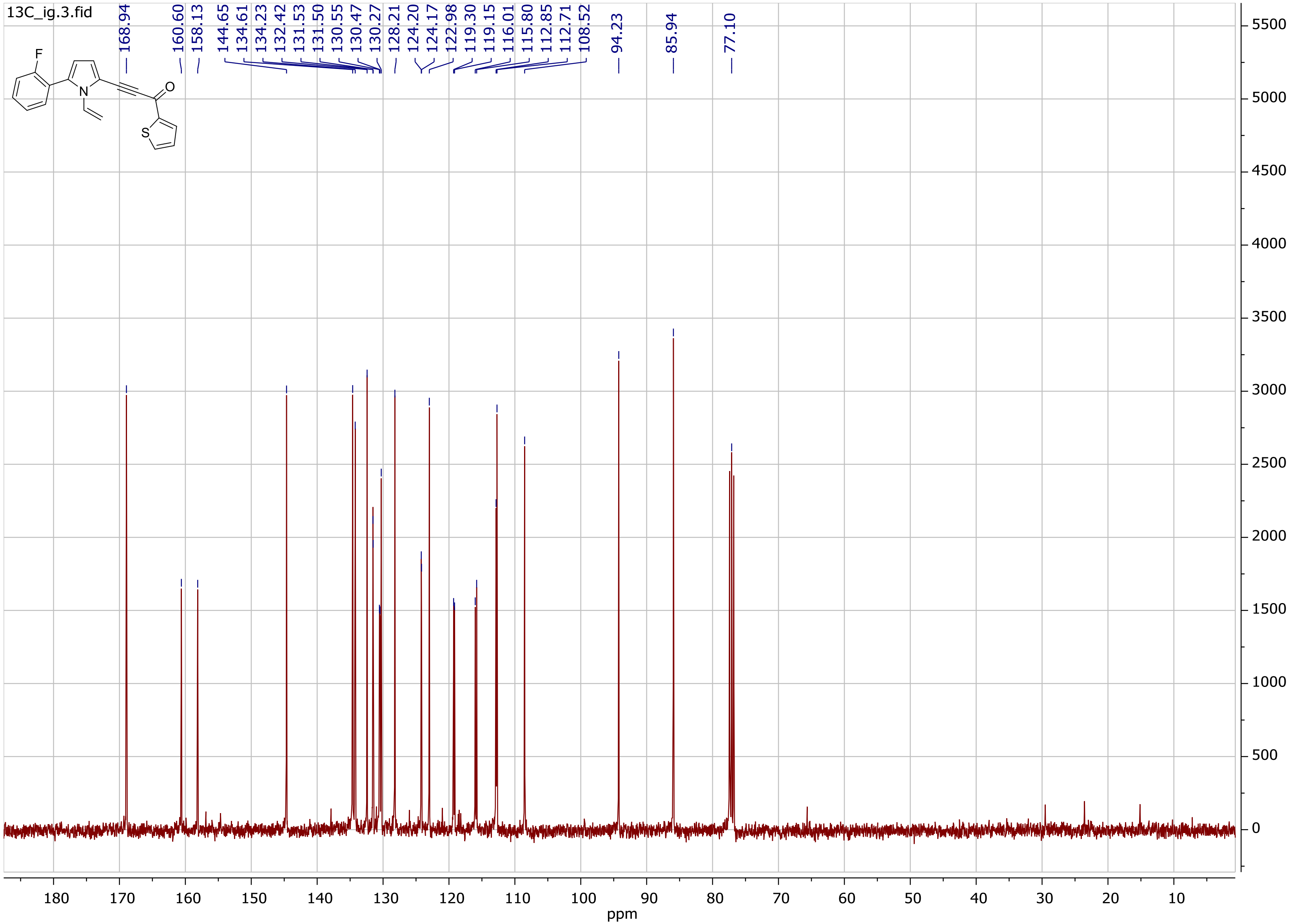

Figure S20: $^{19}$F NMR spectrum ($CDCl_3$) 3-(5-(2-fluorophenyl)-1-vinyl-1*H*-pyrrol-2-yl)-1-(thiophen-2-yl)prop-2-yn-1-one **(1v)**

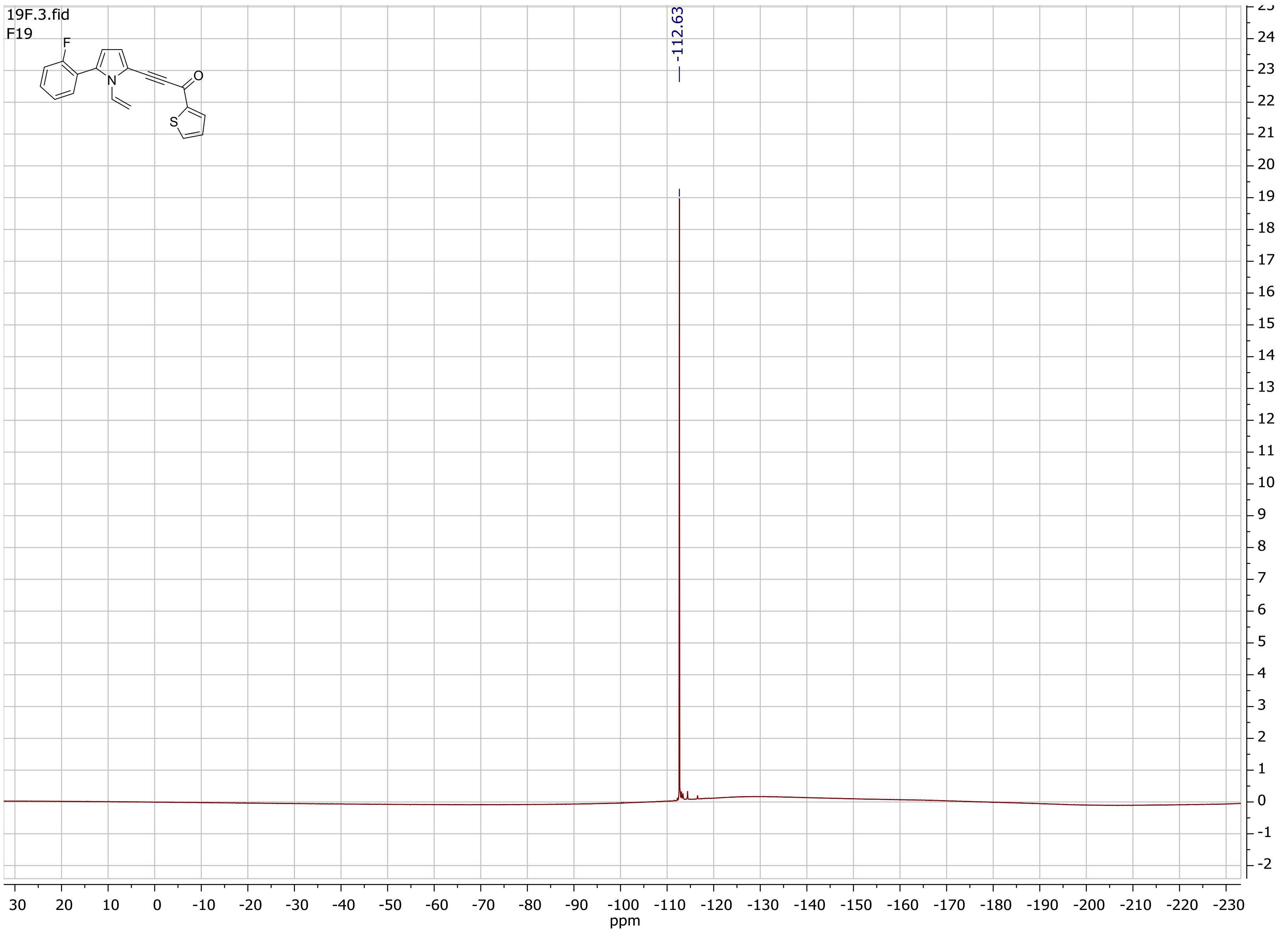

Figure S21: $^{1}$H NMR spectrum ($CDCl_3$) of 4-phenyl-6-(1*H*-pyrrol-2-yl)pyrimidin-2-amine **(3a)**

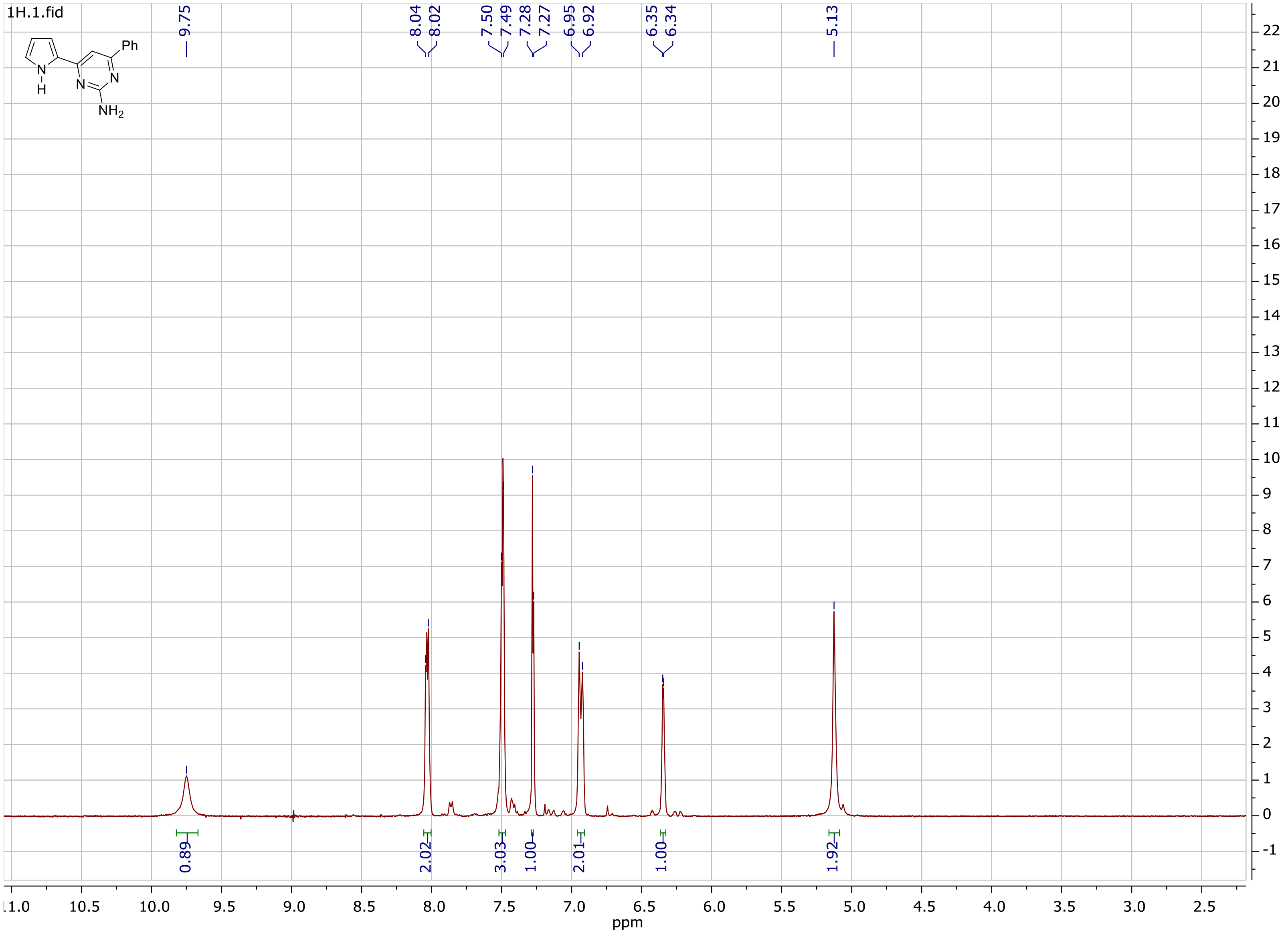

Figure S22: $^{13}$C NMR spectrum ($CDCl_3$) of 4-phenyl-6-(1*H*-pyrrol-2-yl)pyrimidin-2-amine **(3a)**

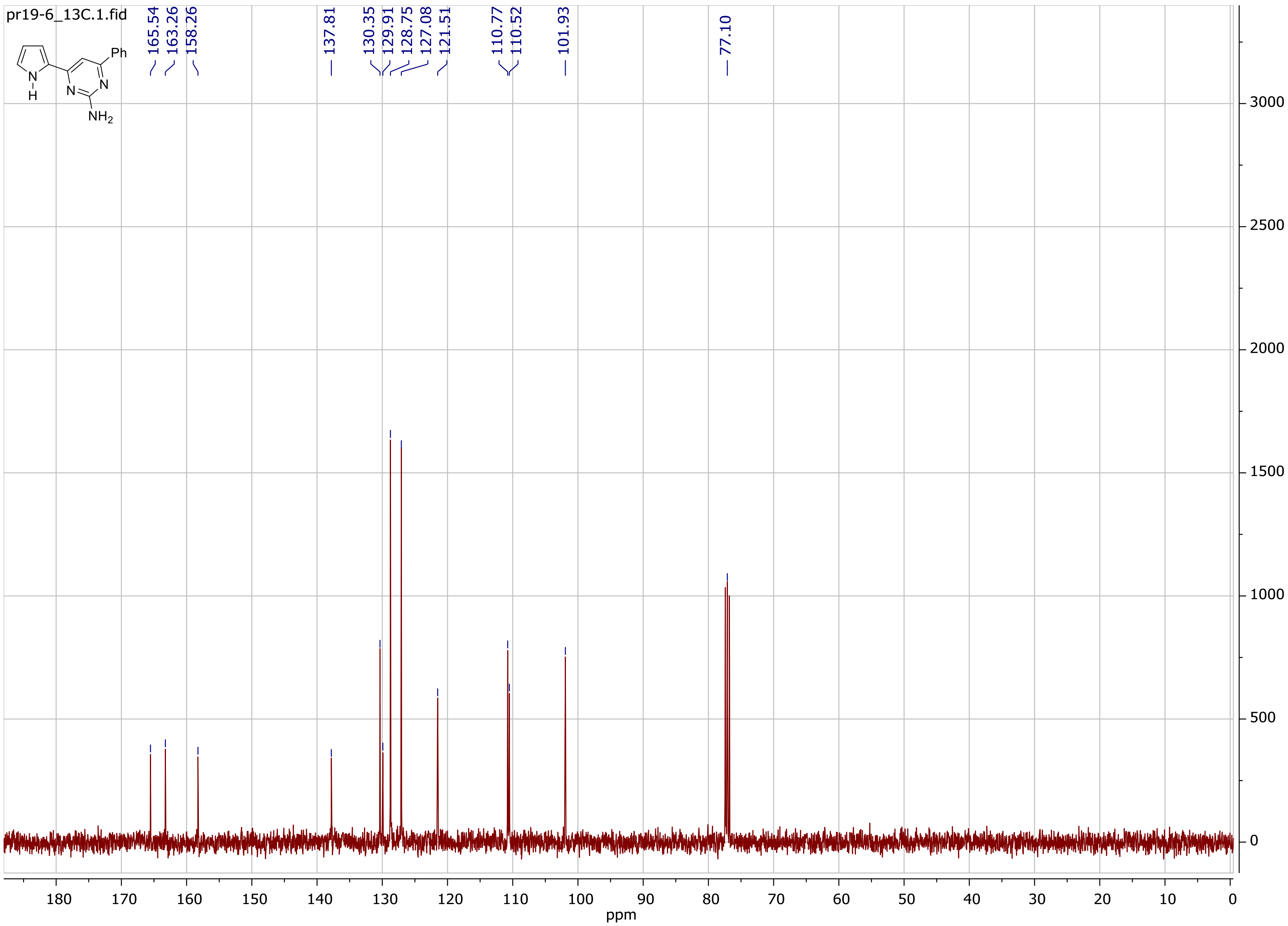

Figure S23: $^{1}$H NMR spectrum ($CDCl_3$) 4-(1-methyl-1*H*-pyrrol-2-yl)-6-phenylpyrimidin-2-amine **(3b)**

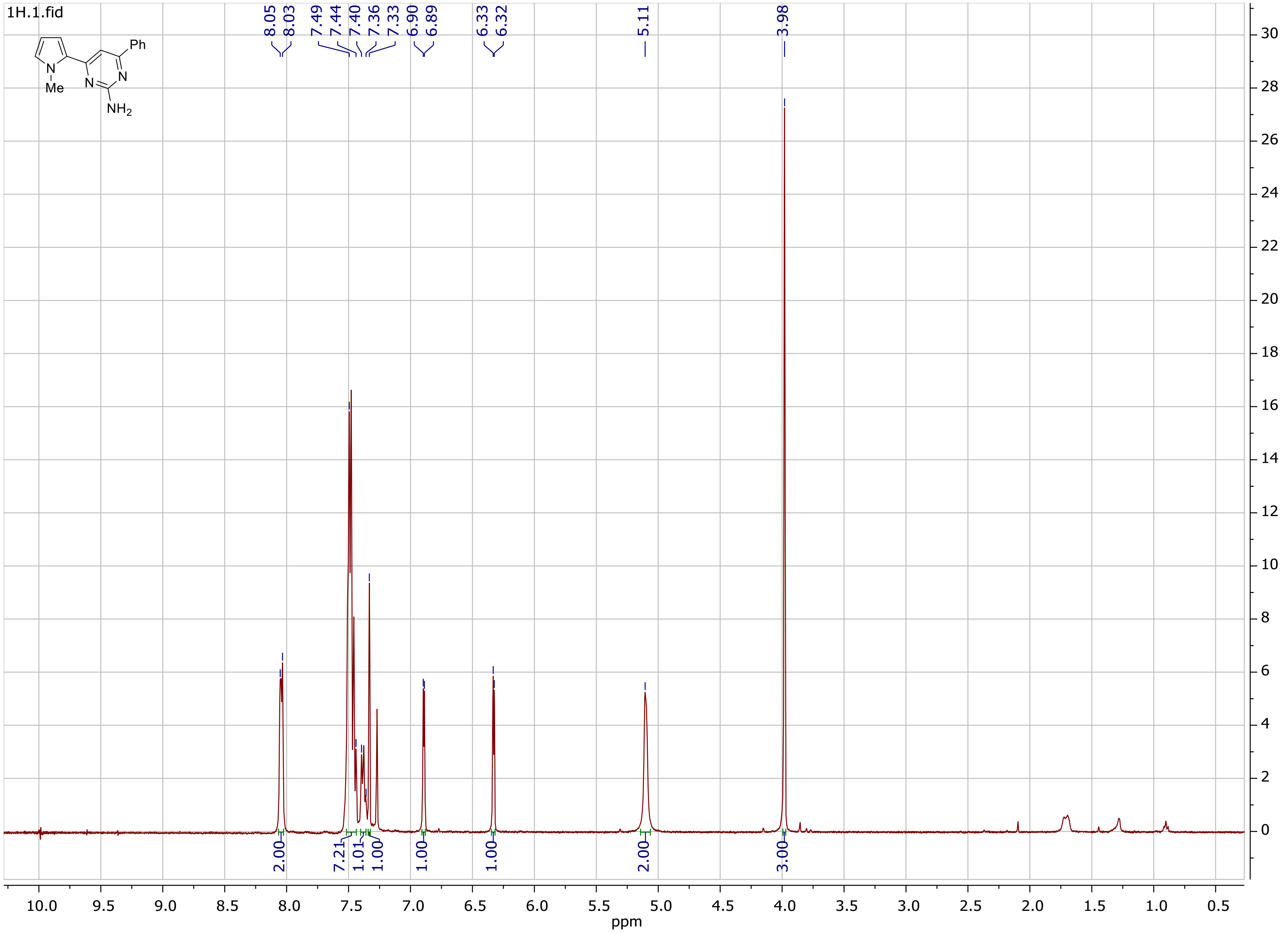

Figure S24: $^{13}$C NMR spectrum ($CDCl_3$) 4-(1-methyl-1*H*-pyrrol-2-yl)-6-phenylpyrimidin-2-amine **(3b)**

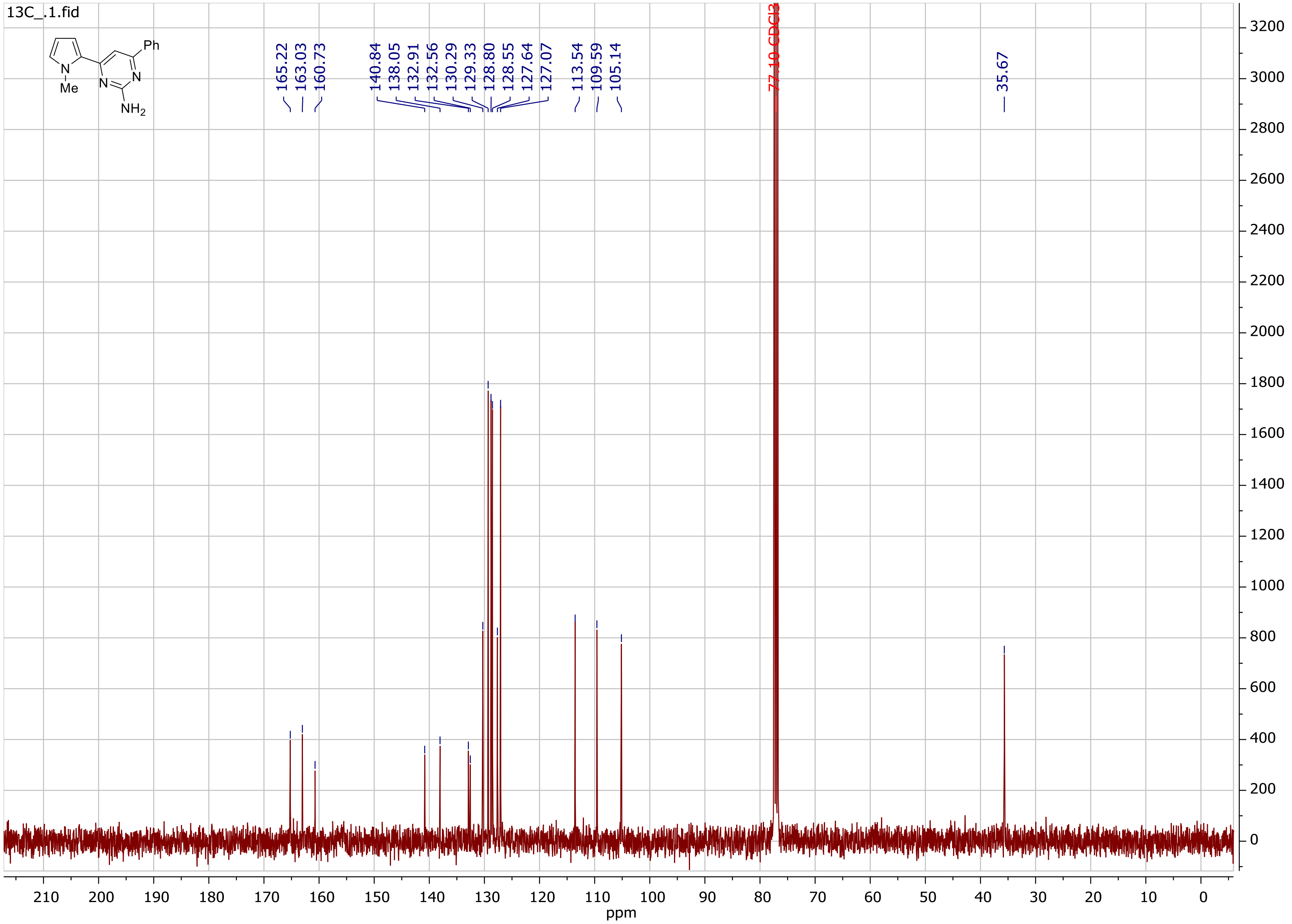

Figure S25: $^{1}$H NMR spectrum ($CDCl_3$) 4-(1-benzyl-1*H*-pyrrol-2-yl)-6-phenylpyrimidin-2-amine **(3c)**

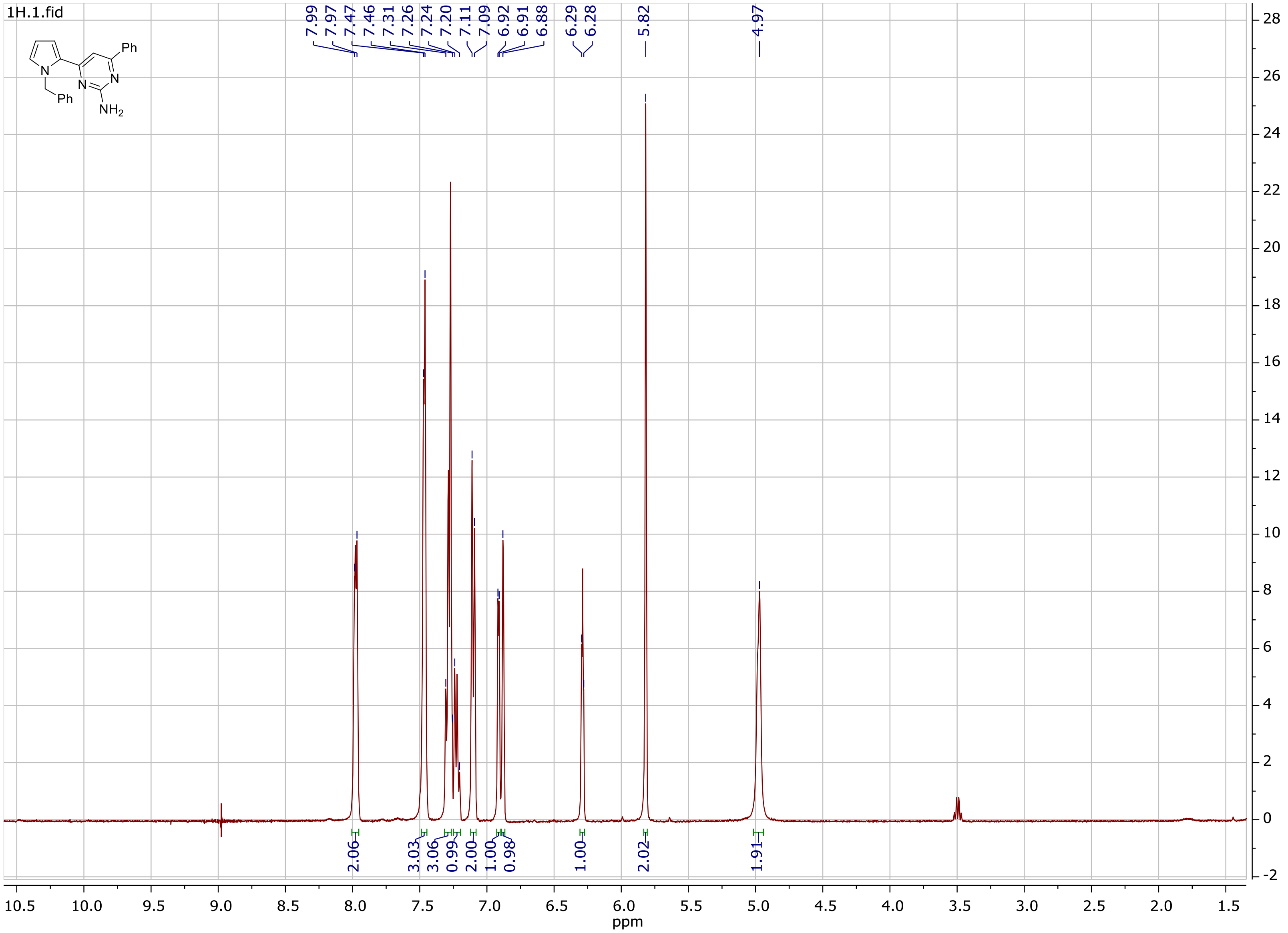

Figure S26: $^{13}$C NMR spectrum ($CDCl_3$) 4-(1-benzyl-1*H*-pyrrol-2-yl)-6-phenylpyrimidin-2-amine **(3c)**

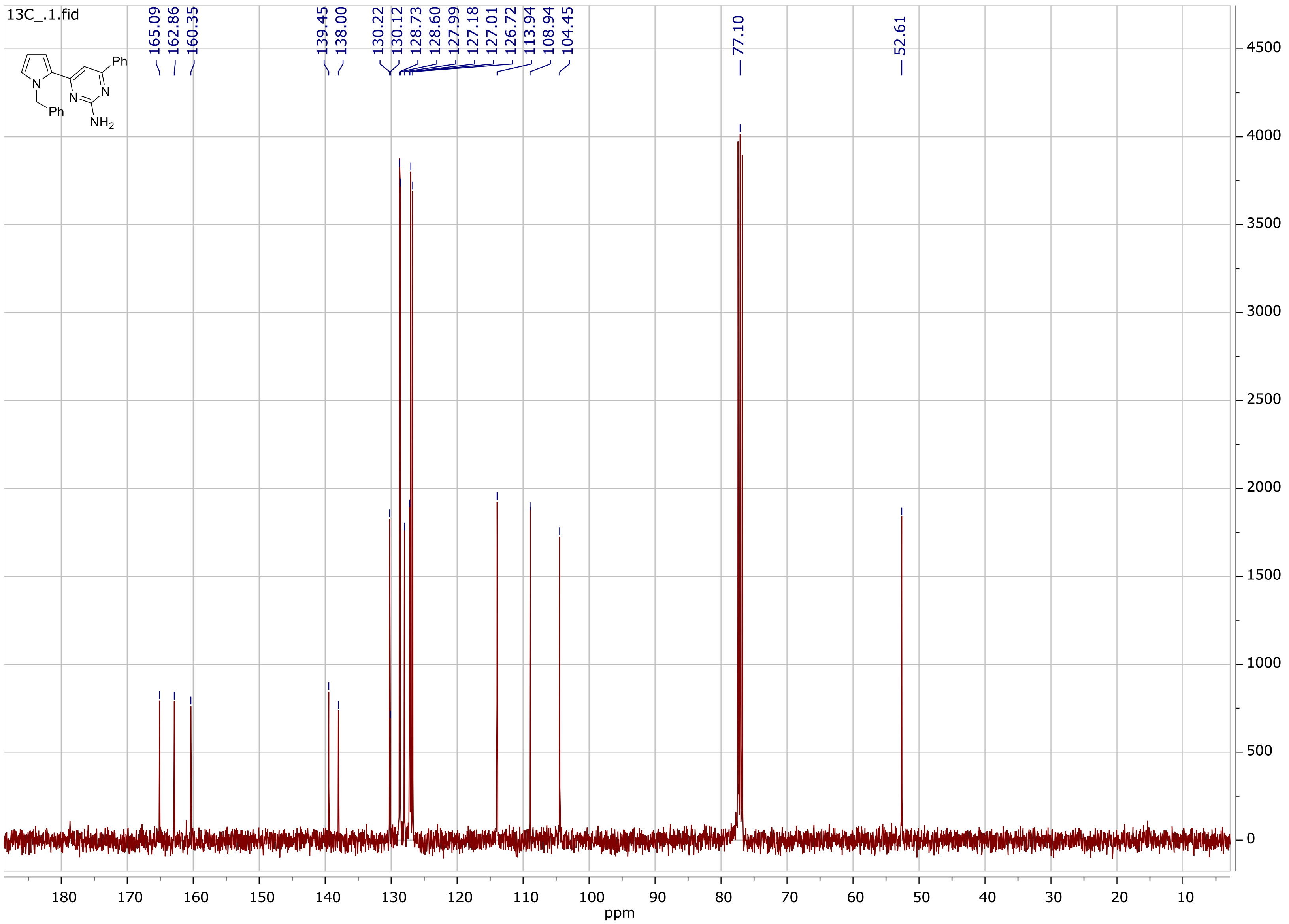

Figure S27: $^{1}$H NMR spectrum ($CDCl_3$) 4-(4-ethyl-5-propyl-1*H*-pyrrol-2-yl)-6-phenylpyrimidin-2-amine **(3d)**

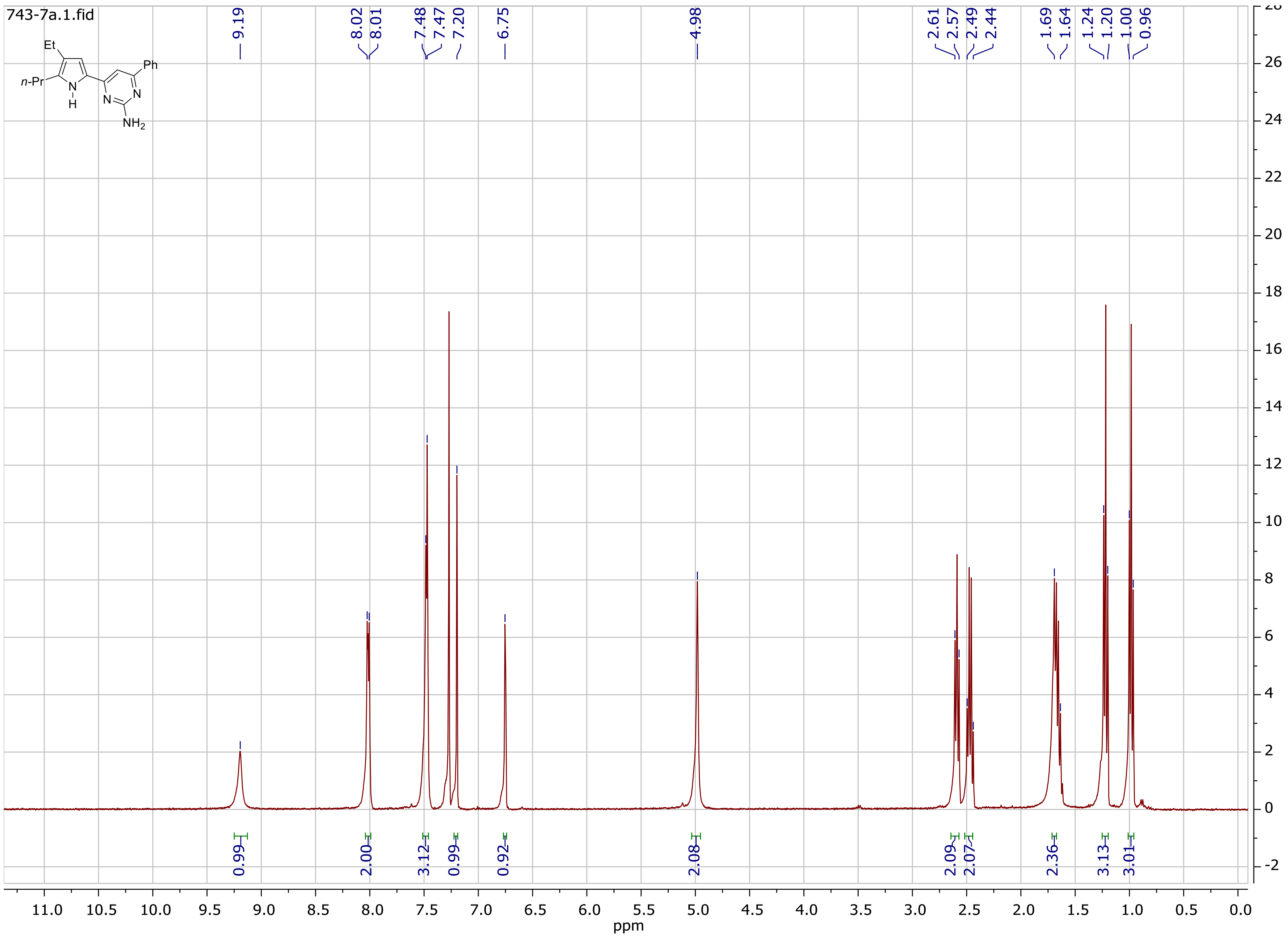

Figure S28: $^{13}$C NMR spectrum ($CDCl_3$) 4-(4-ethyl-5-propyl-1*H*-pyrrol-2-yl)-6-phenylpyrimidin-2-amine **(3d)**

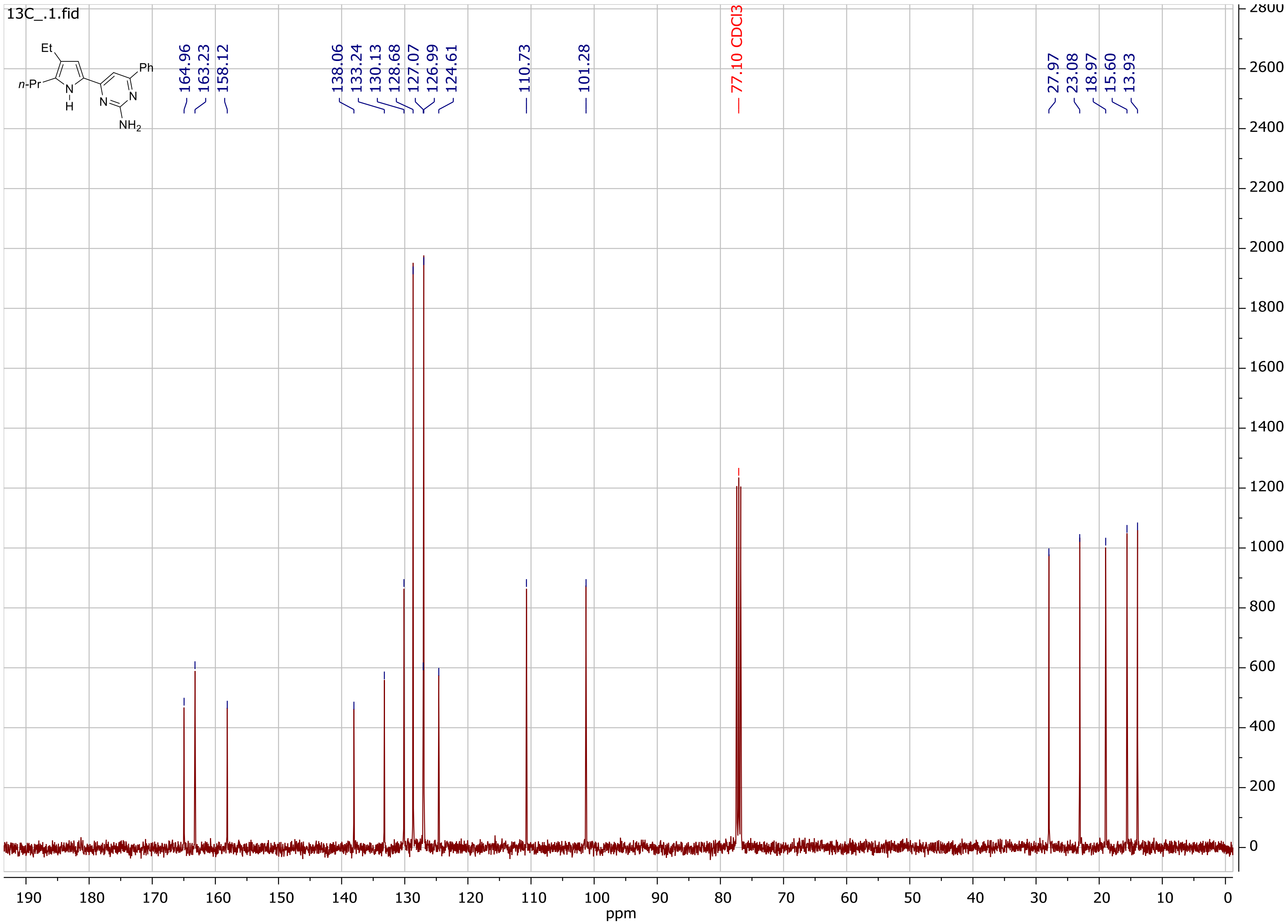

Figure S29: $^{1}$H NMR spectrum ($CDCl_3$) 4-(5-butyl-4-propyl-1*H*-pyrrol-2-yl)-6-phenylpyrimidin-2-amine **(3e)**

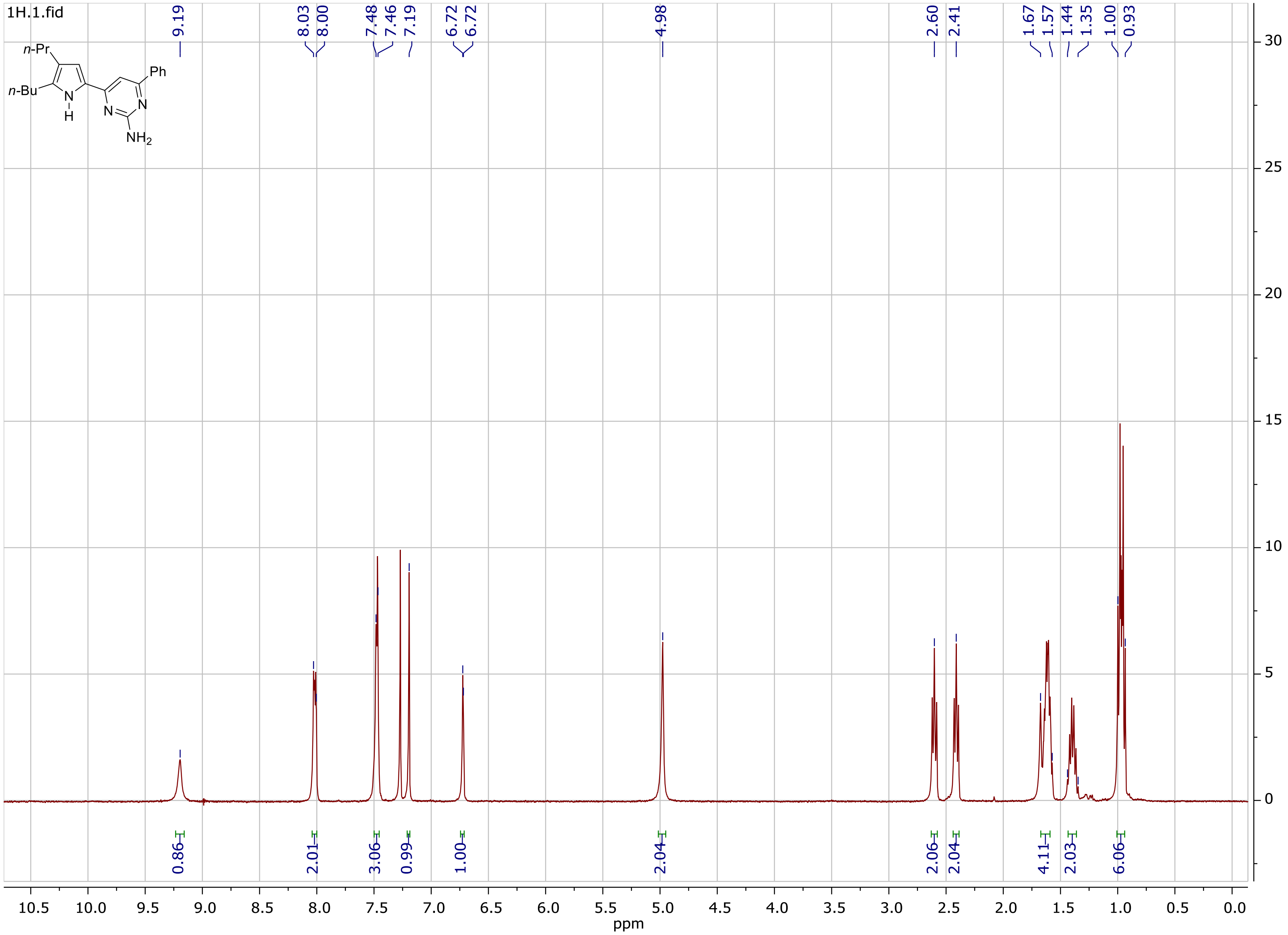

Figure S30: $^{13}$C NMR spectrum ($CDCl_3$) 4-(5-butyl-4-propyl-1*H*-pyrrol-2-yl)-6-phenylpyrimidin-2-amine **(3e)**

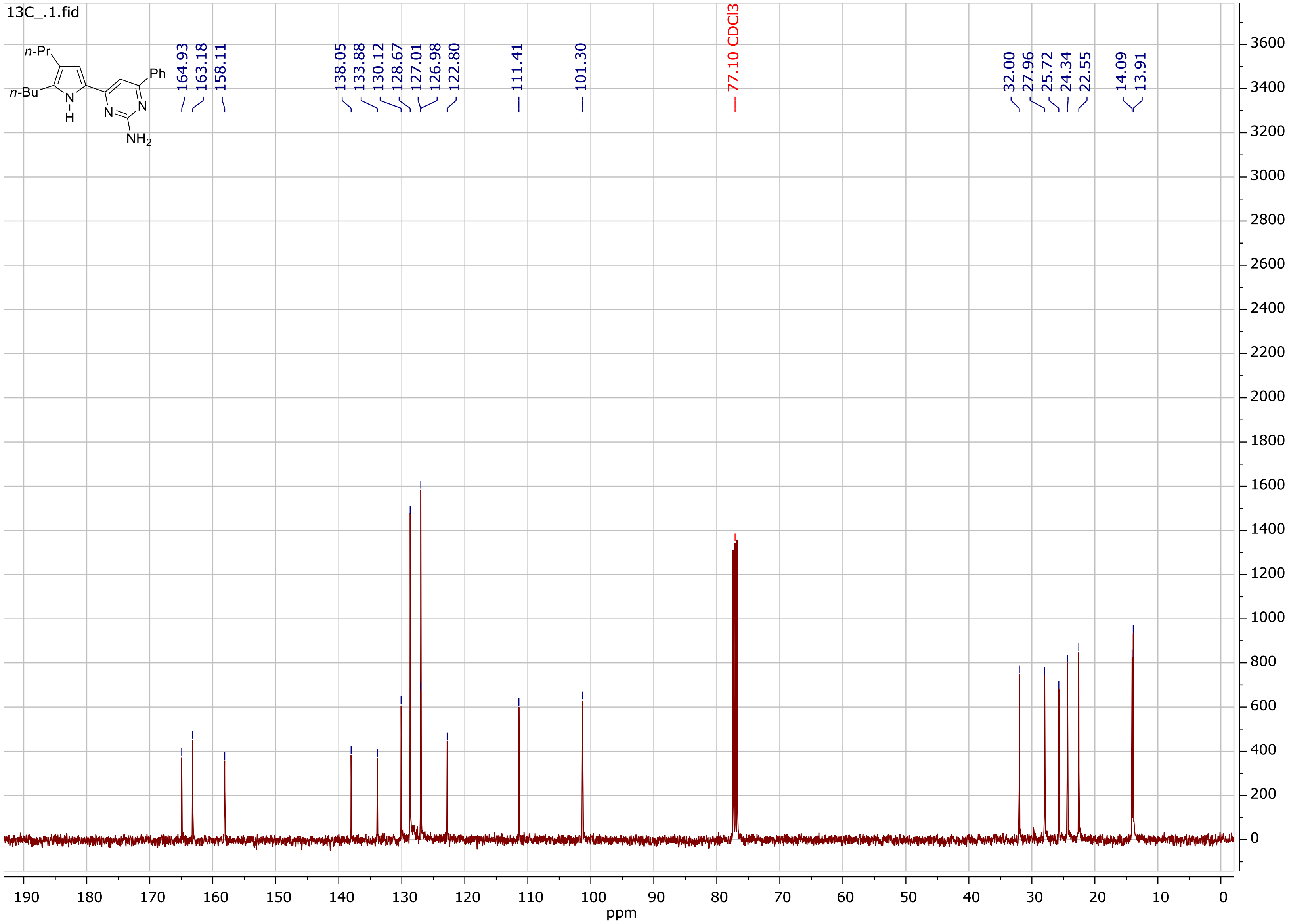

Figure S31: $^1$H NMR spectrum ($CDCl_3$) 4-phenyl-6-(4,5,6,7-tetrahydro-1*H*-indol-2-yl)pyrimidin-2-amine **(3f)**

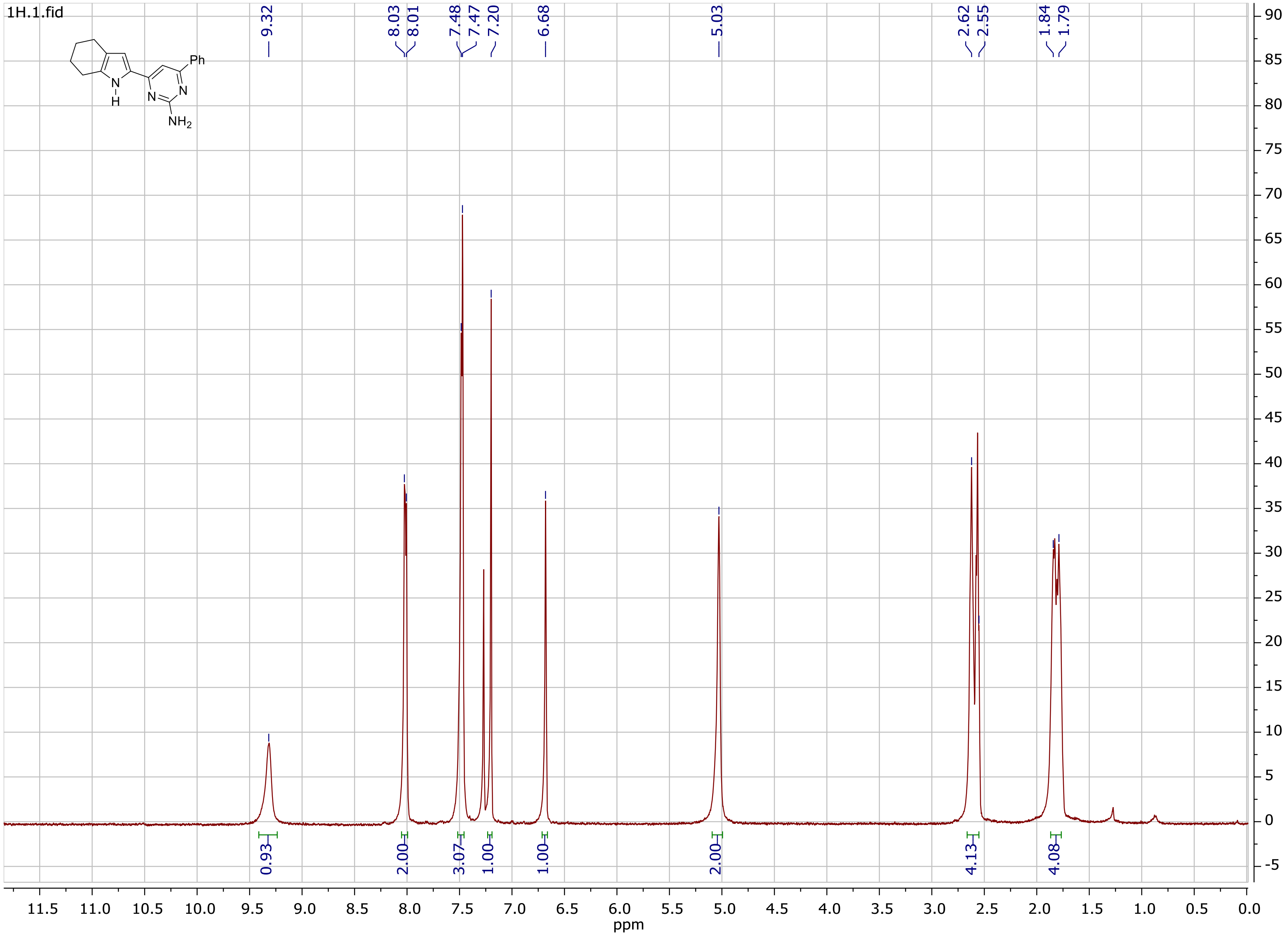

Figure S32: $^{13}$C NMR spectrum ($CDCl_3$) 4-phenyl-6-(4,5,6,7-tetrahydro-1*H*-indol-2-yl)pyrimidin-2-amine **(3f)**

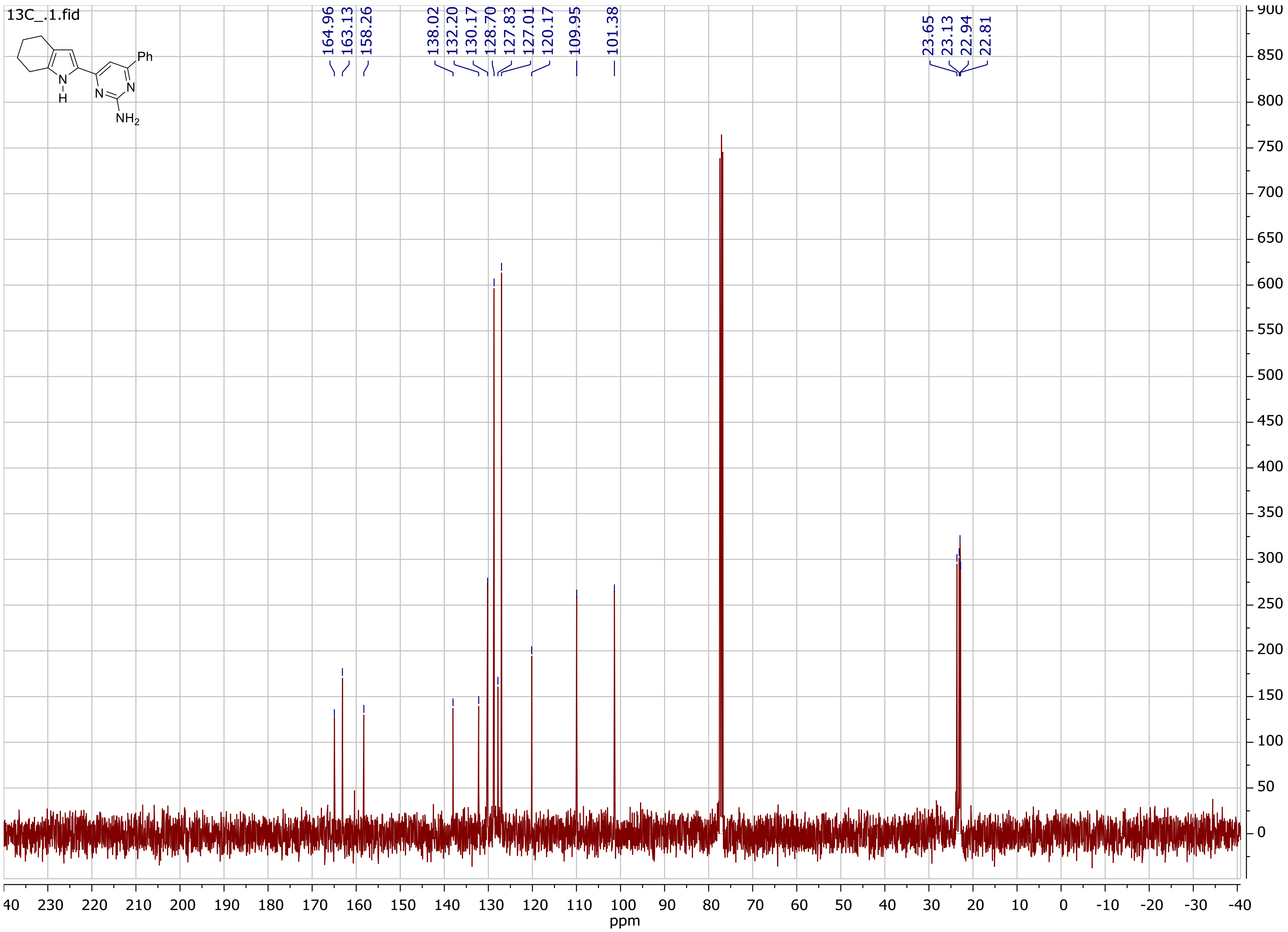

Figure S33: $^{1}$H NMR spectrum ($CDCl_3$) 4-(1-methyl-4,5,6,7-tetrahydro-1*H*-indol-2-yl)-6-phenylpyrimidin-2-amine **(3g)**

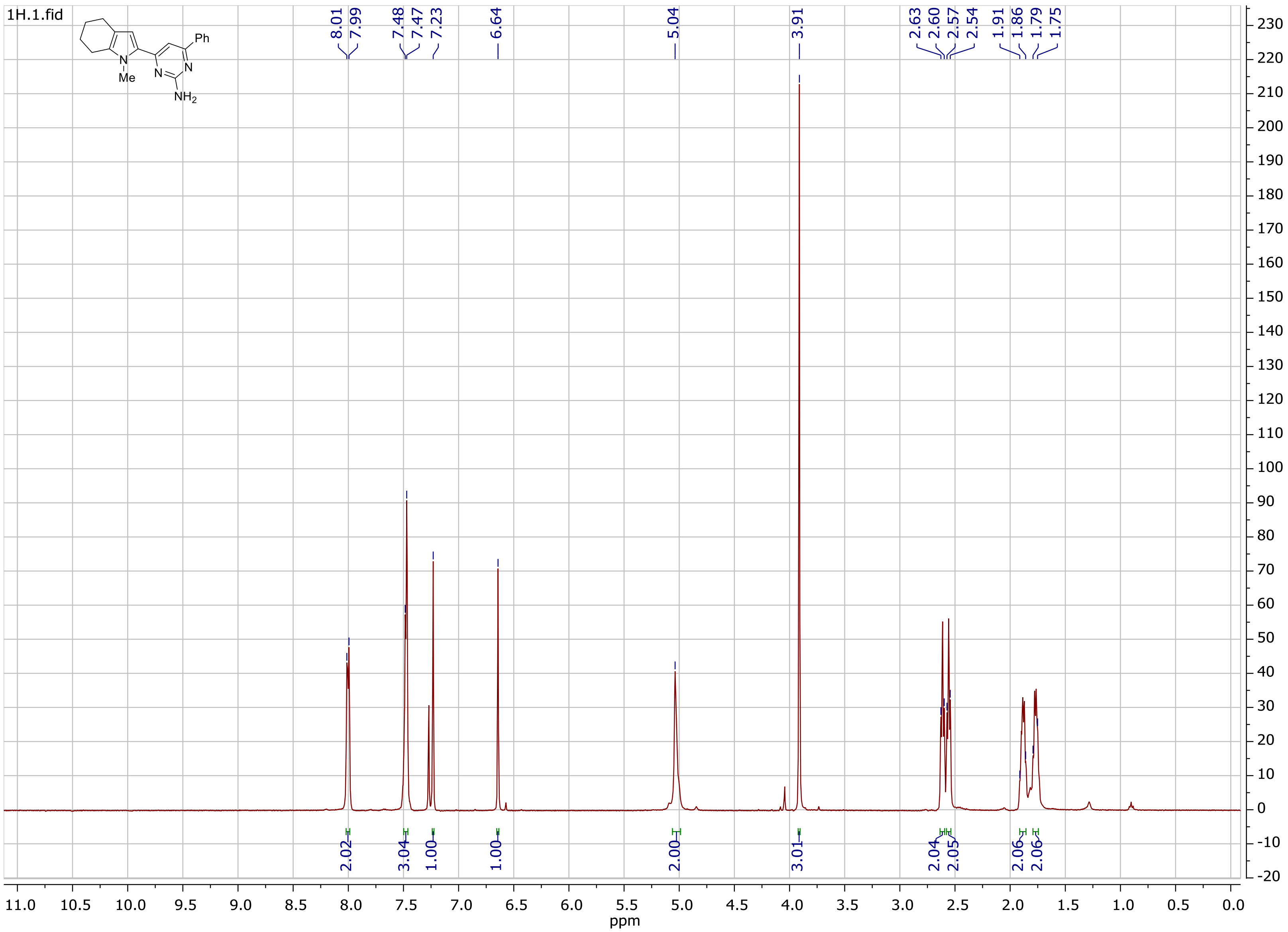

Figure S34: $^{13}$C NMR spectrum (DMSO-d$_6$) 4-(1-methyl-4,5,6,7-tetrahydro-1*H*-indol-2-yl)-6-phenylpyrimidin-2-amine **(3g)**

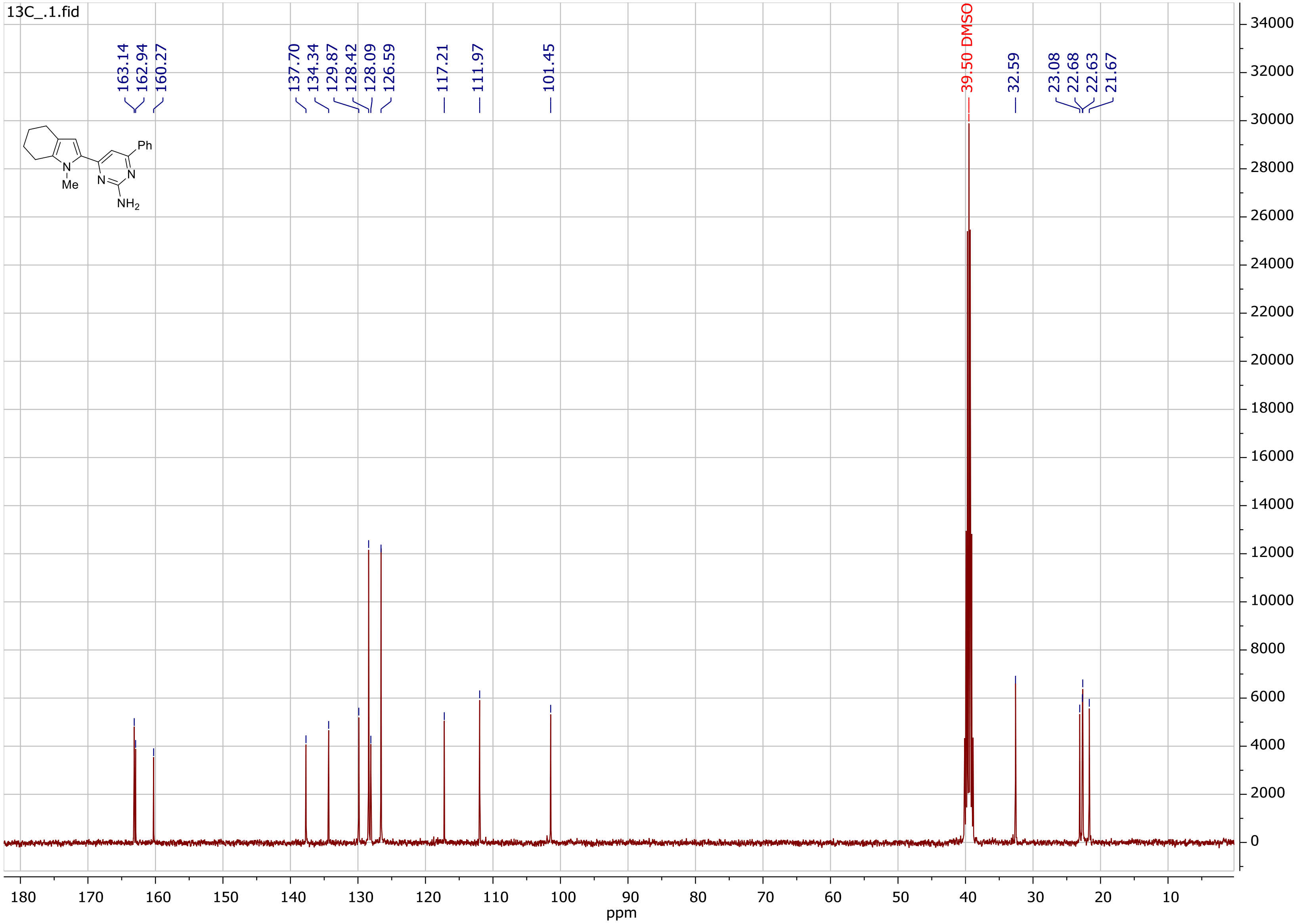

Figure S35: $^{1}$H NMR spectrum ($CDCl_3$) 4-(2-furyl)-6-(1-methyl-4,5,6,7-tetrahydro-1*H*-indol-2-yl)pyrimidin-2-amine **(3h)**

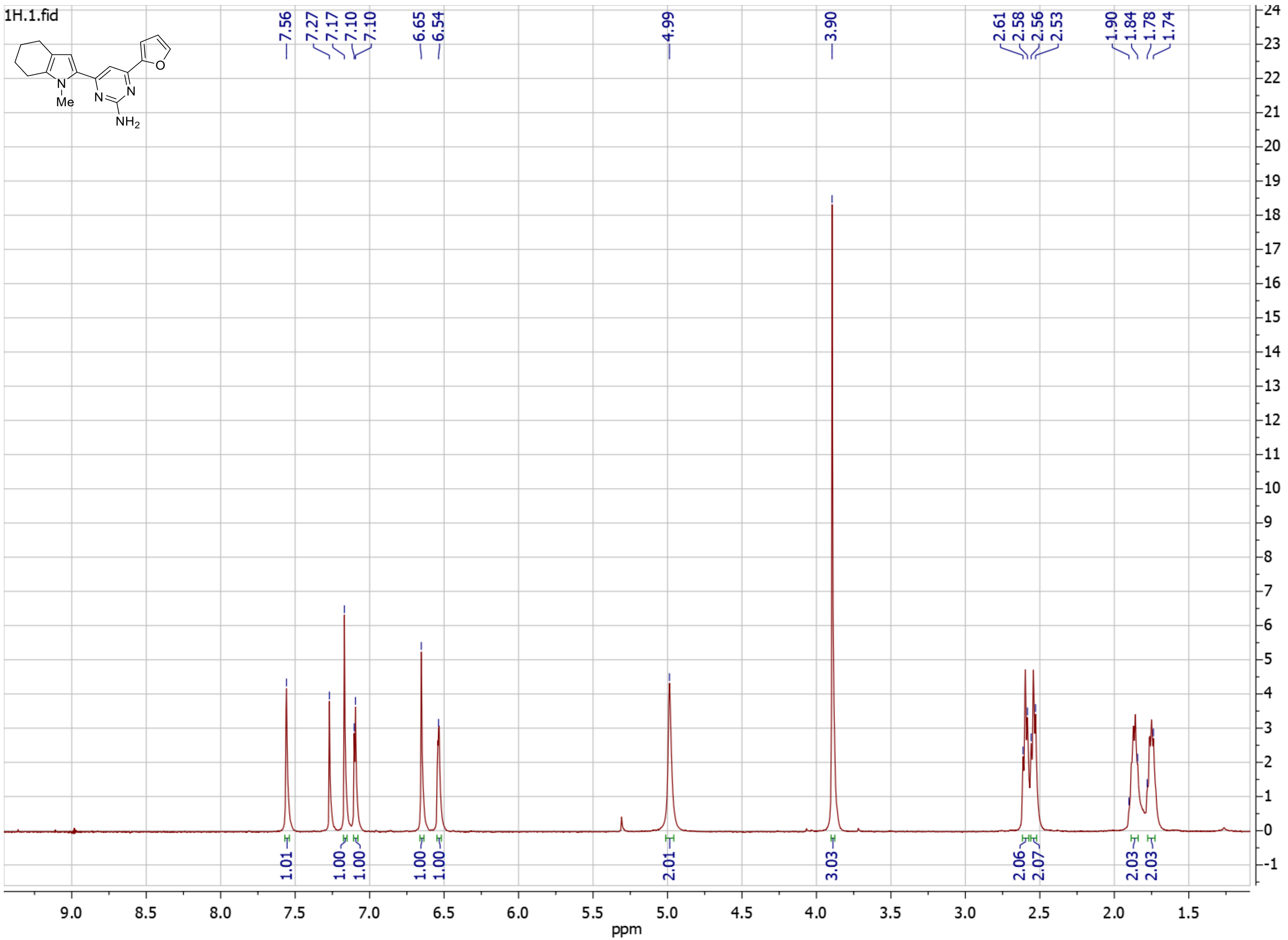

Figure S36: $^{13}$C NMR spectrum ($CDCl_3$) 4-(2-furyl)-6-(1-methyl-4,5,6,7-tetrahydro-1*H*-indol-2-yl)pyrimidin-2-amine **(3h)**

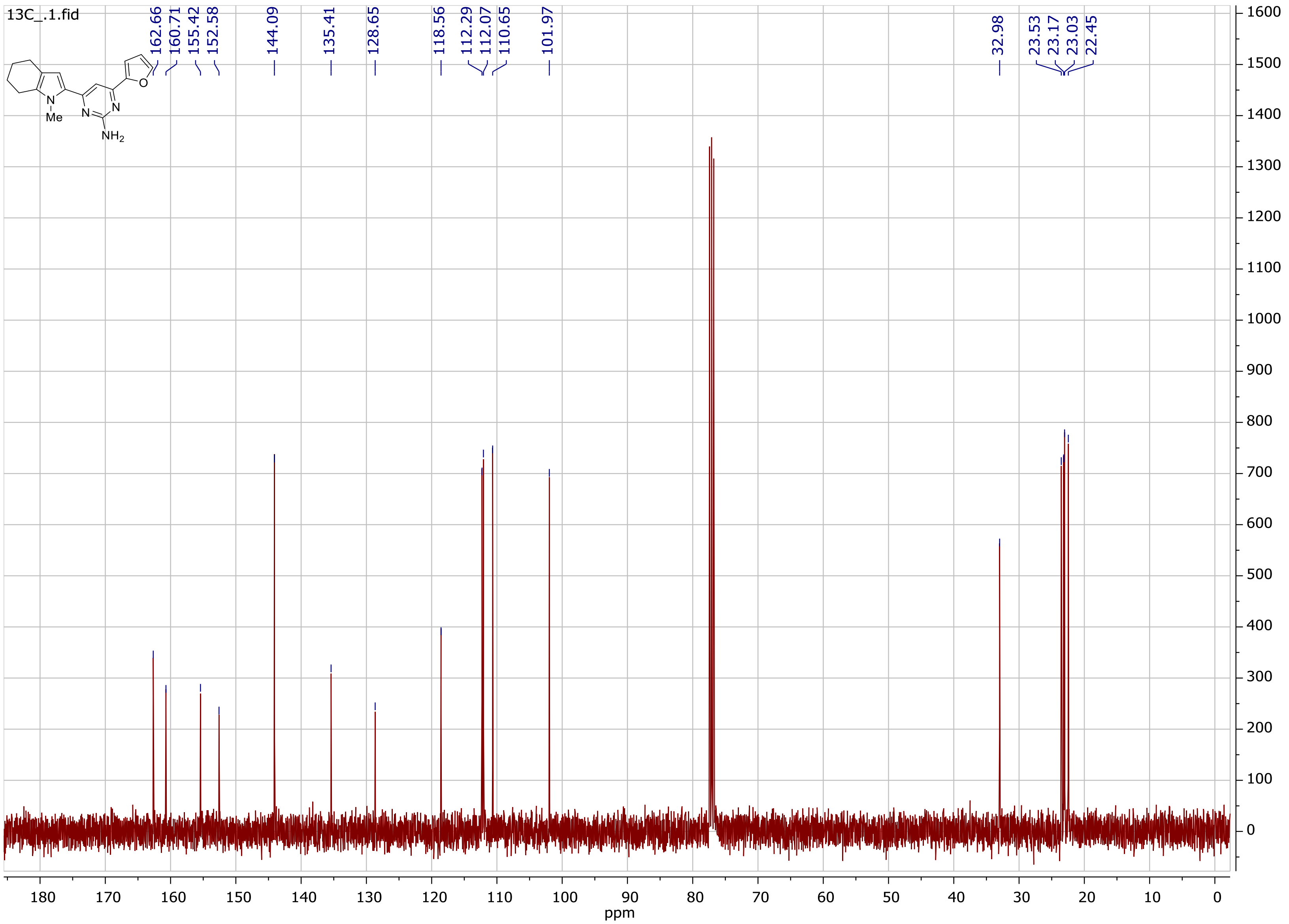

Figure S37: $^{1}$H NMR spectrum ($CDCl_3$) 4-(1-benzyl-4,5,6,7-tetrahydro-1*H*-indol-2-yl)-6-phenylpyrimidin-2-amine **(3i)**

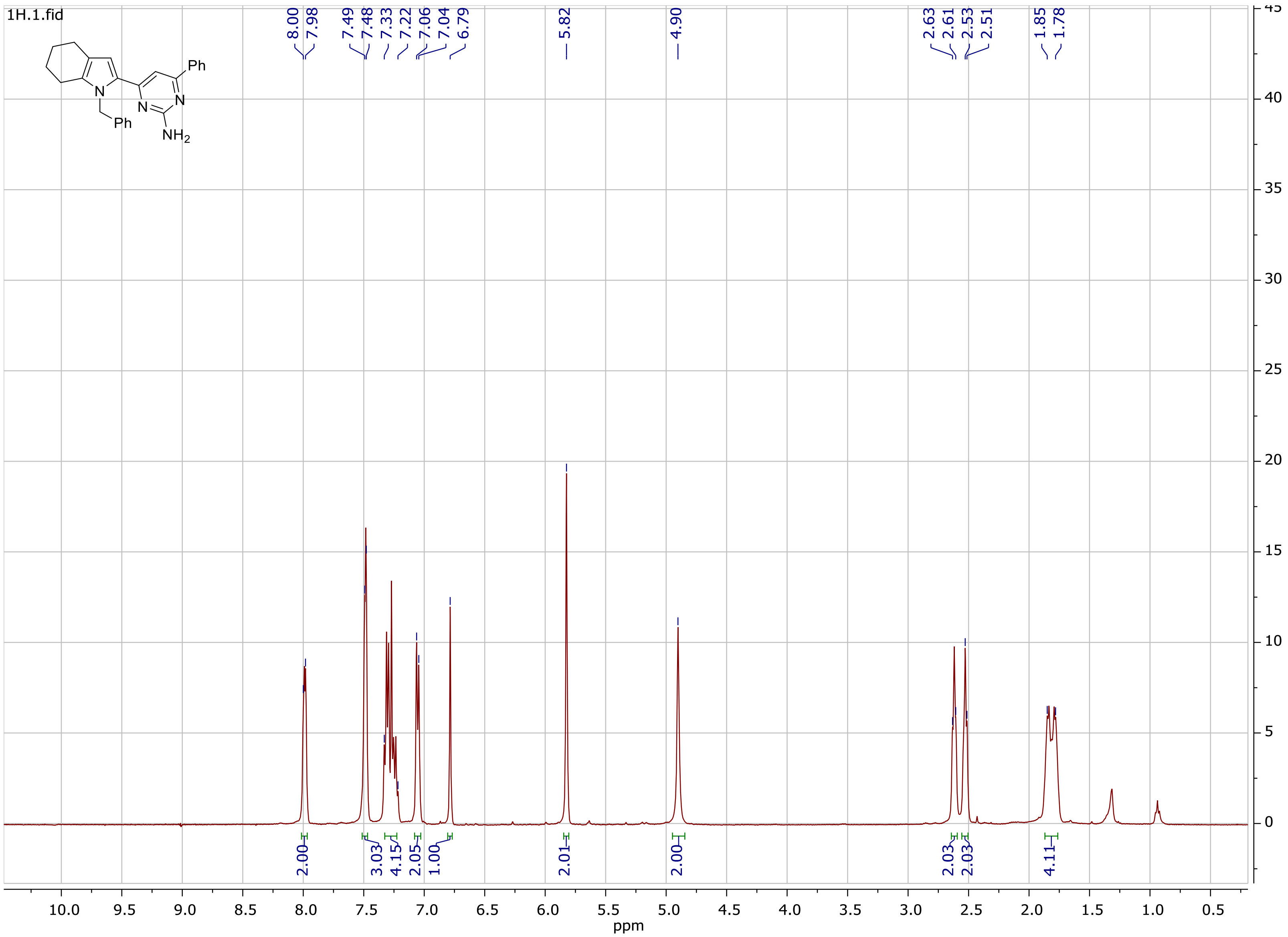

Figure S38: $^{13}$C NMR spectrum ($CDCl_3$) 4-(1-benzyl-4,5,6,7-tetrahydro-1*H*-indol-2-yl)-6-phenylpyrimidin-2-amine **(3i)**

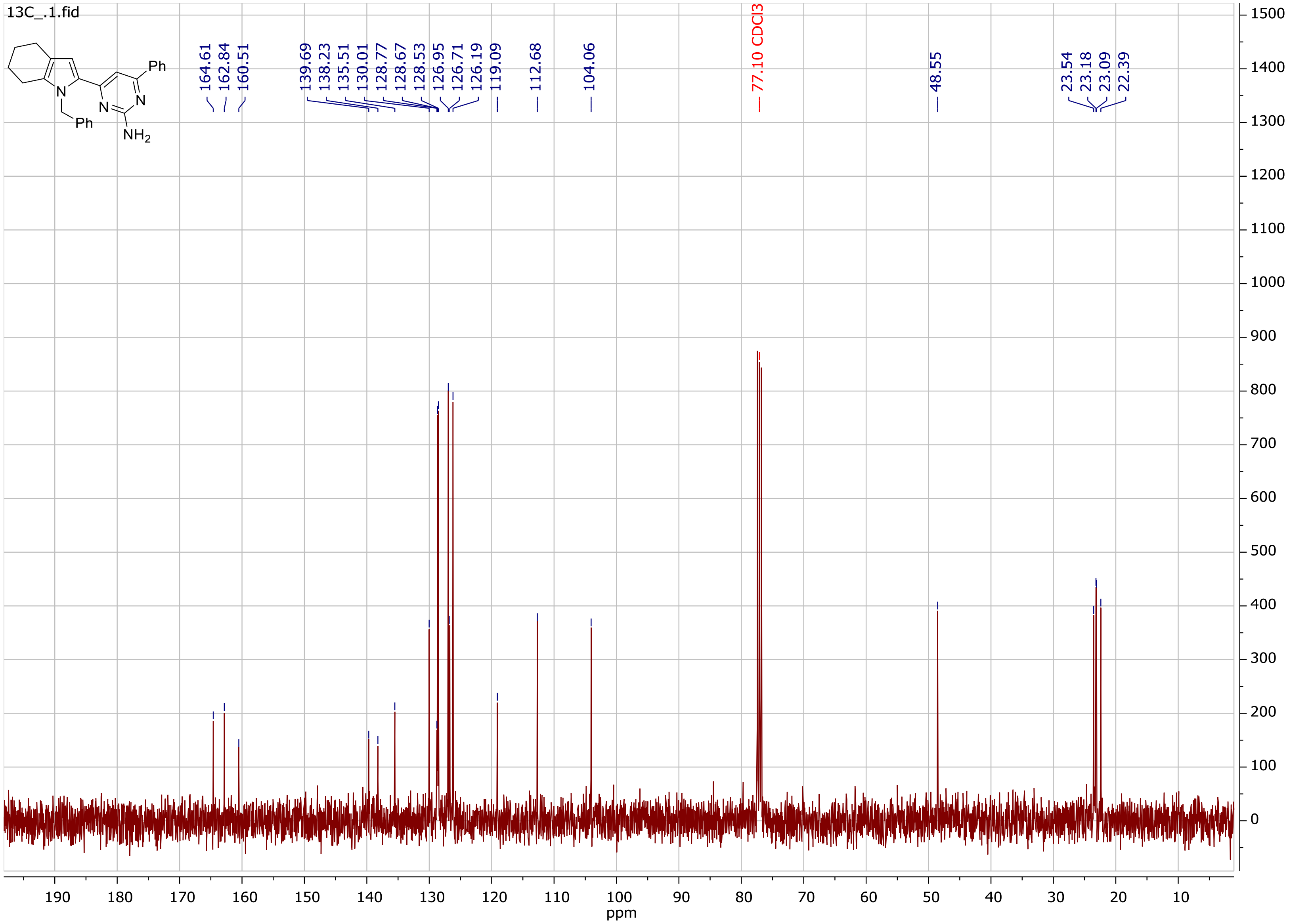

Figure S39: $^1$H NMR spectrum ($CDCl_3$) 4-(1-benzyl-4,5,6,7-tetrahydro-1*H*-indol-2-yl)-6-(2-furyl)pyrimidin-2-amine **(3j)**

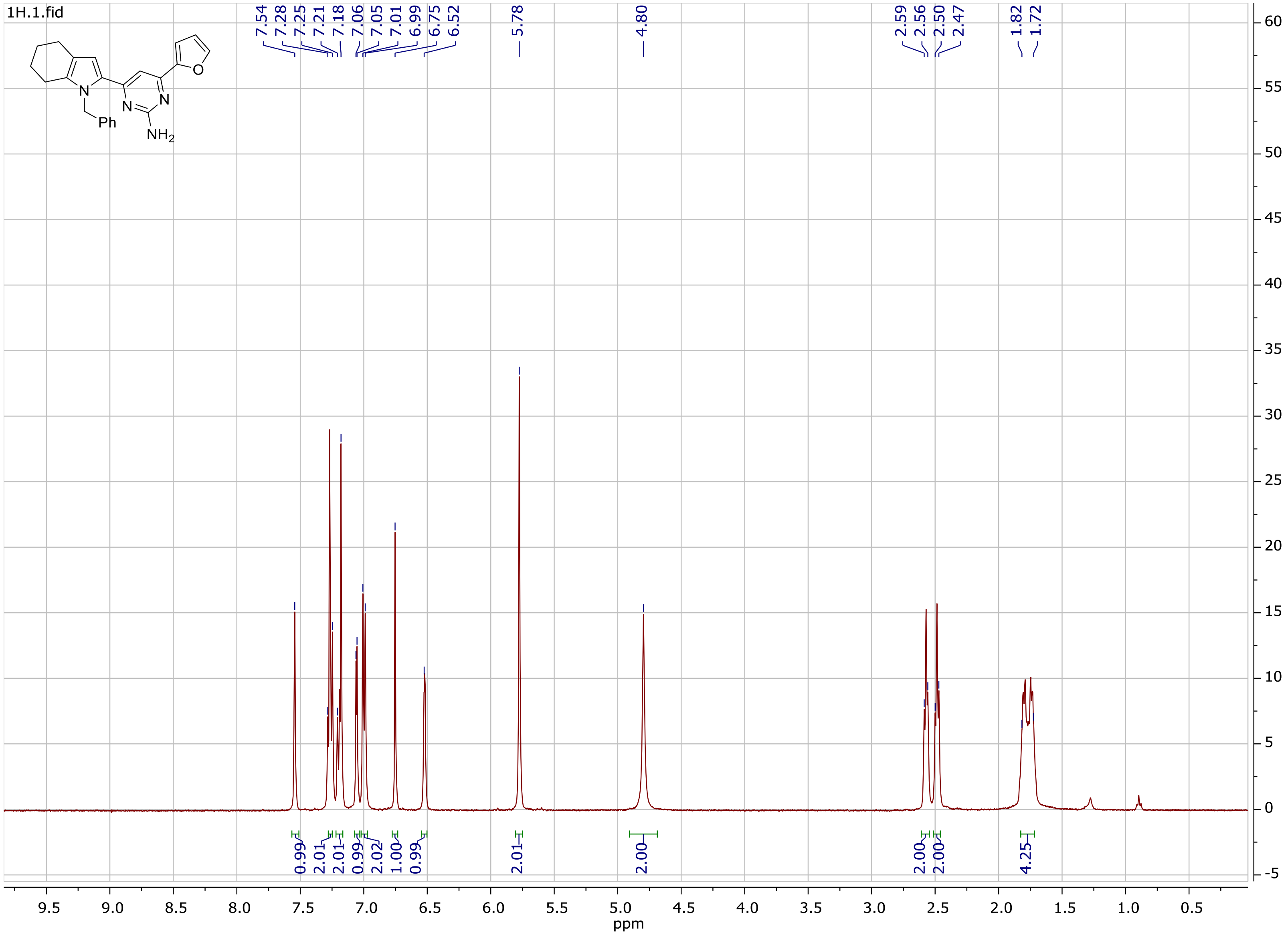

Figure S40: $^{13}$C NMR spectrum ($CDCl_3$) 4-(1-benzyl-4,5,6,7-tetrahydro-1*H*-indol-2-yl)-6-(2-furyl)pyrimidin-2-amine **(3j)**

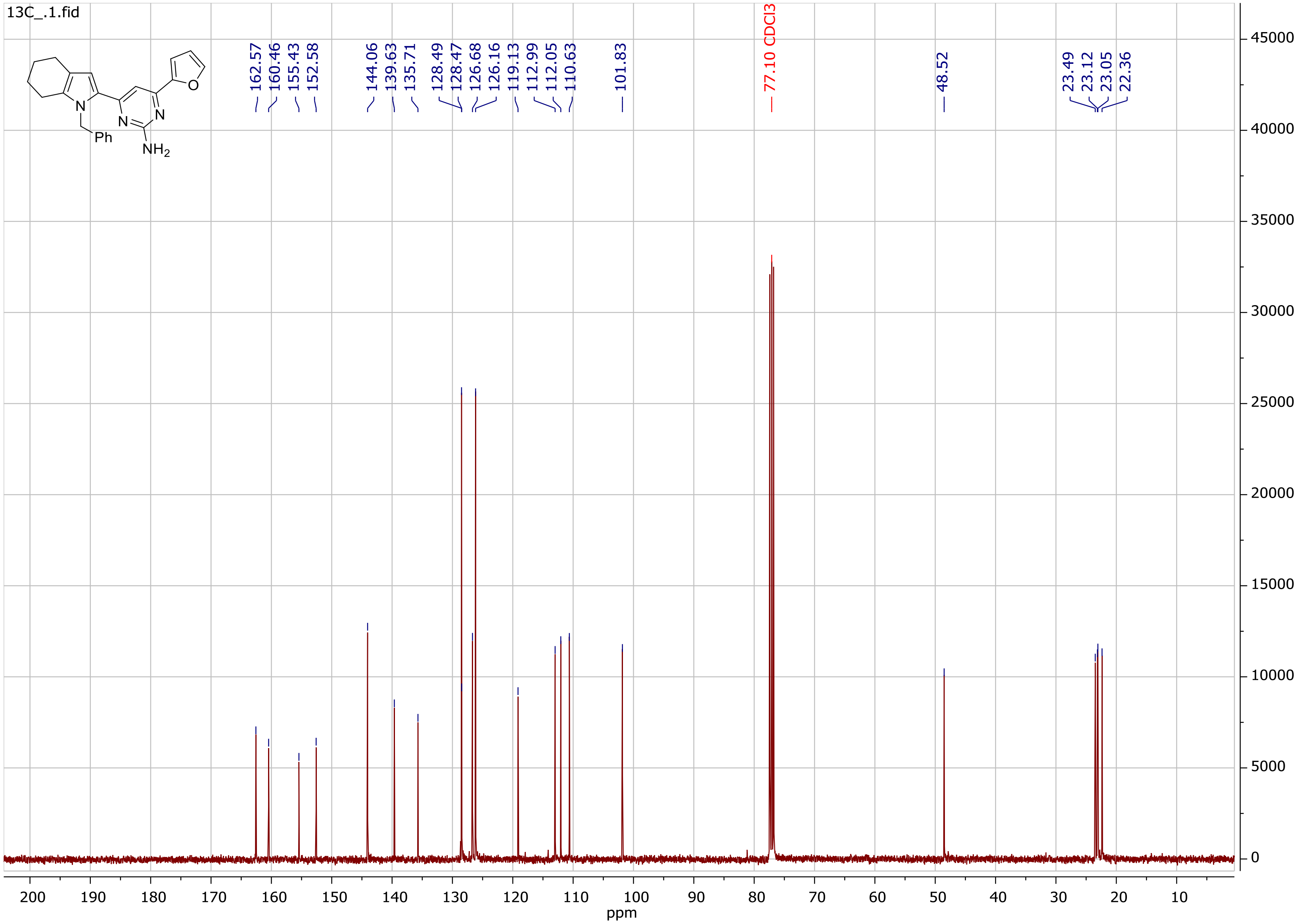

Figure S41: $^{1}$H NMR spectrum ($CDCl_3$) 4-phenyl-6-(1-vinyl-4,5,6,7-tetrahydro-1*H*-indol-2-yl)pyrimidin-2-amine **(3k)**

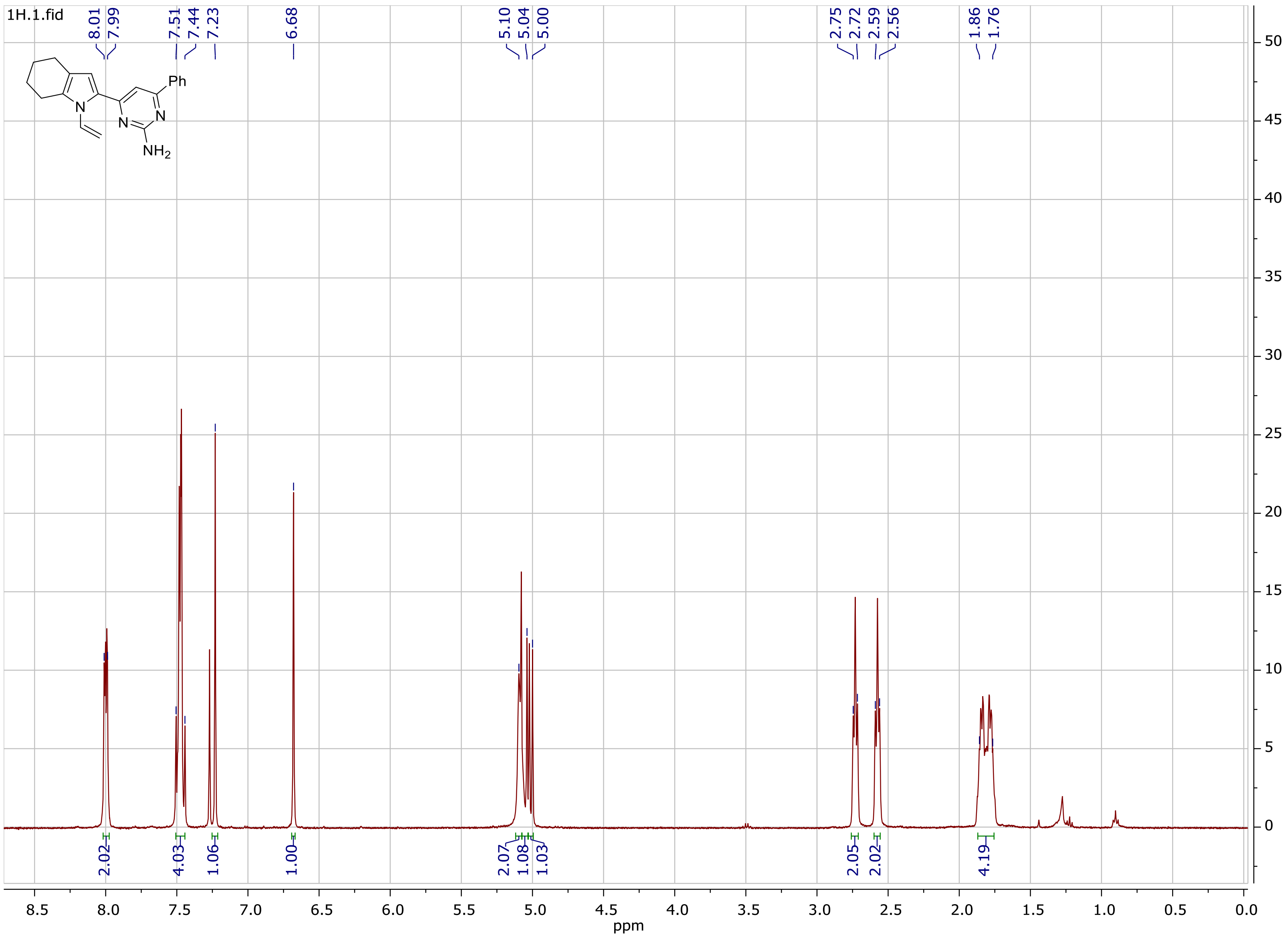

Figure S42: $^{13}C$ NMR spectrum ($CDCl_3$) 4-phenyl-6-(1-vinyl-4,5,6,7-tetrahydro-1*H*-indol-2-yl)pyrimidin-2-amine **(3k)**

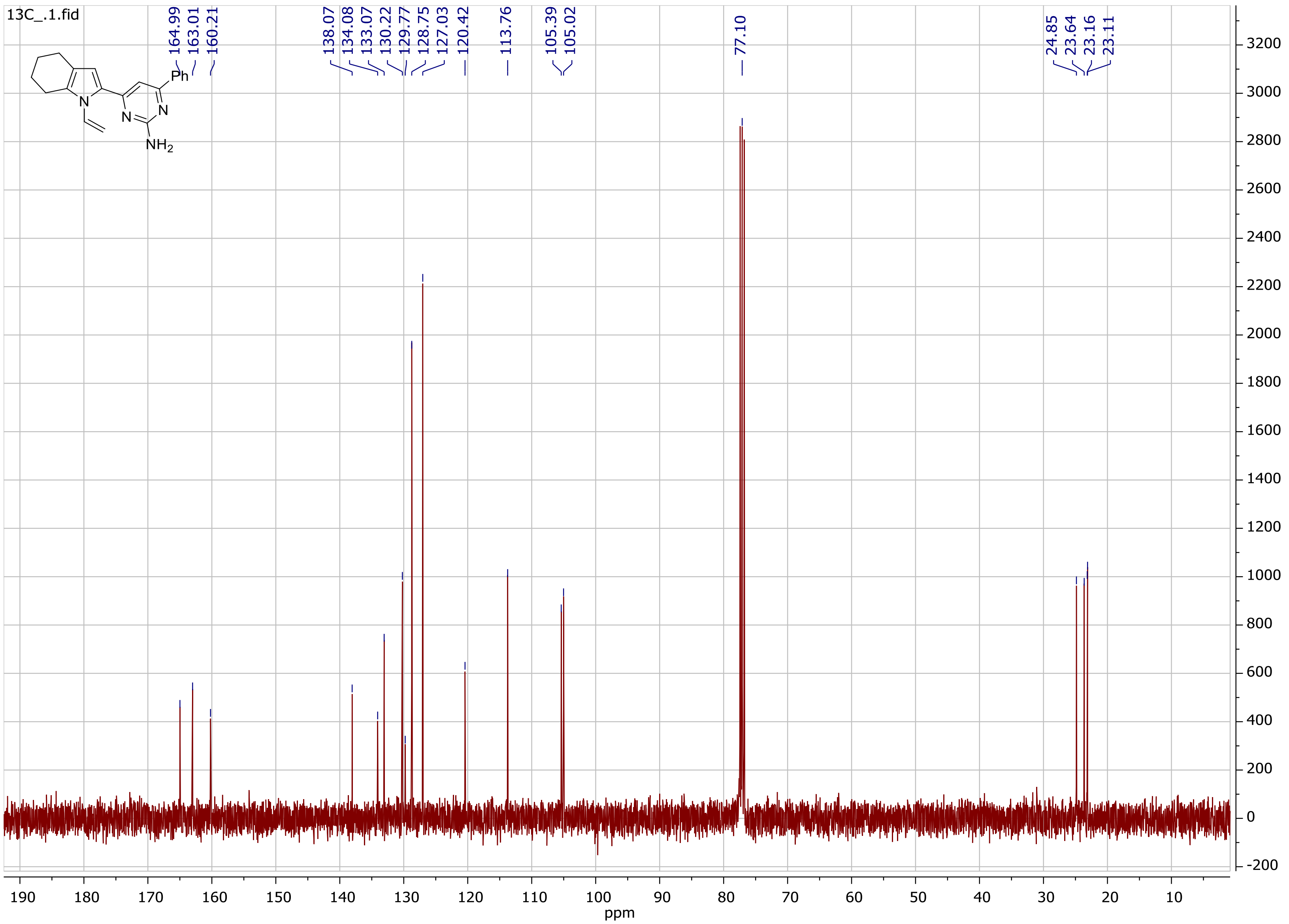

Figure S43: $^{1}$H NMR spectrum (DMSO-d$_6$) 4-phenyl-6-(5-phenyl-1*H*-pyrrol-2-yl)pyrimidin-2-amine **(3l)**

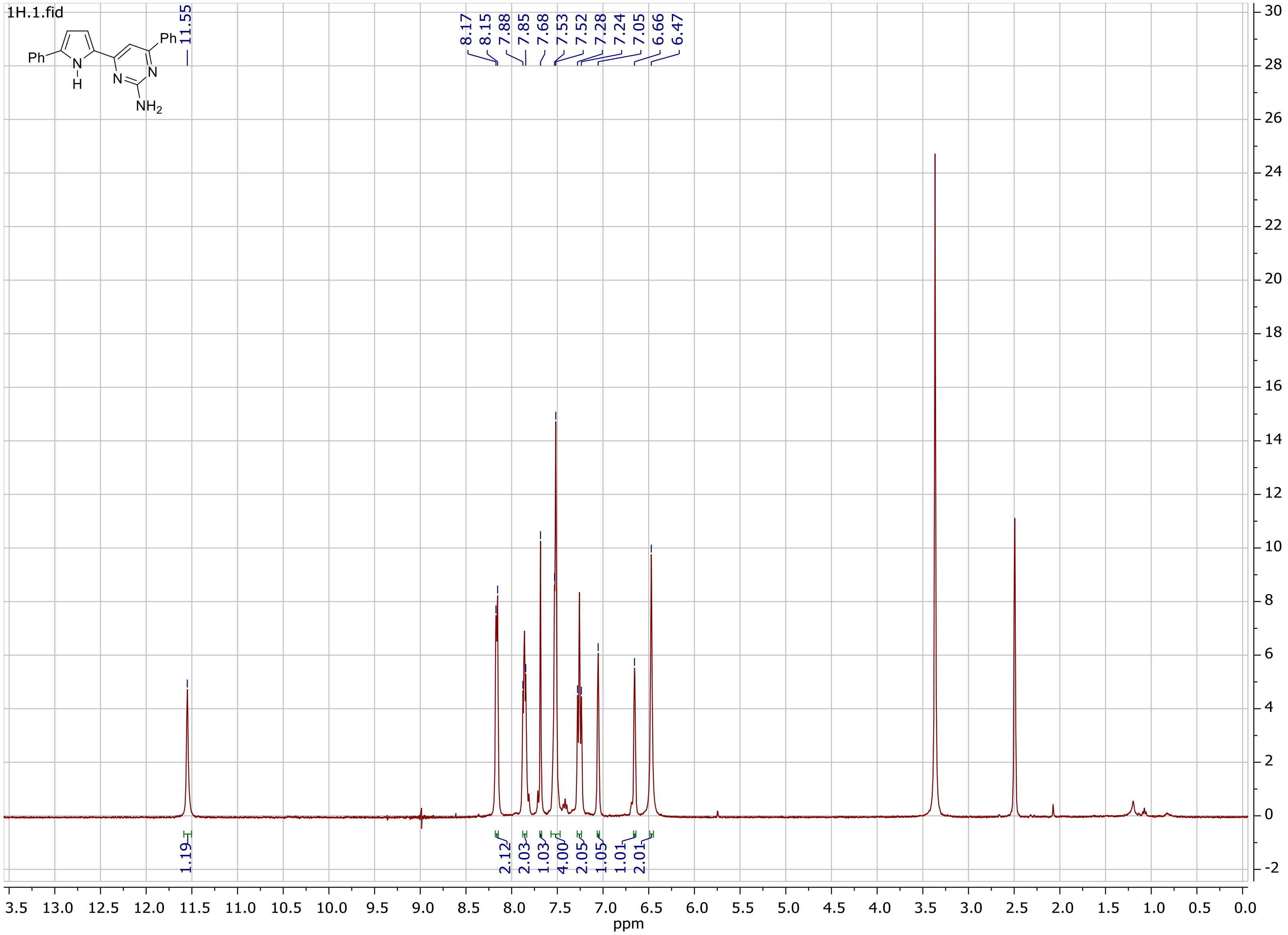

Figure S44: $^{13}$C NMR spectrum (DMSO-d$_6$) 4-phenyl-6-(5-phenyl-1*H*-pyrrol-2-yl)pyrimidin-2-amine **(3l)**

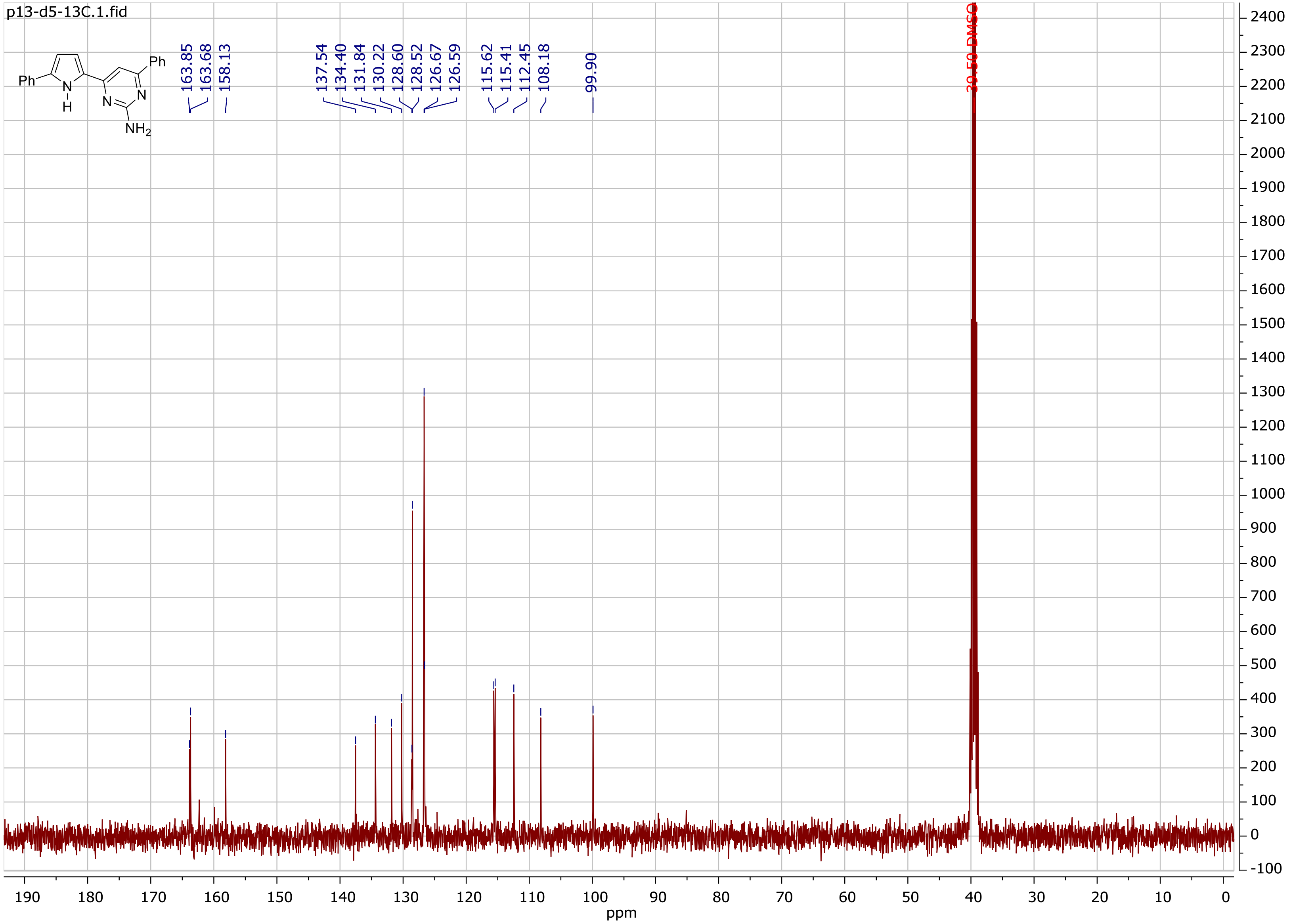

Figure S45: $^{1}$H NMR spectrum (DMSO-d$_6$) 4-(2-furyl)-6-(5-phenyl-1*H*-pyrrol-2-yl)pyrimidin-2-amine **(3m)**

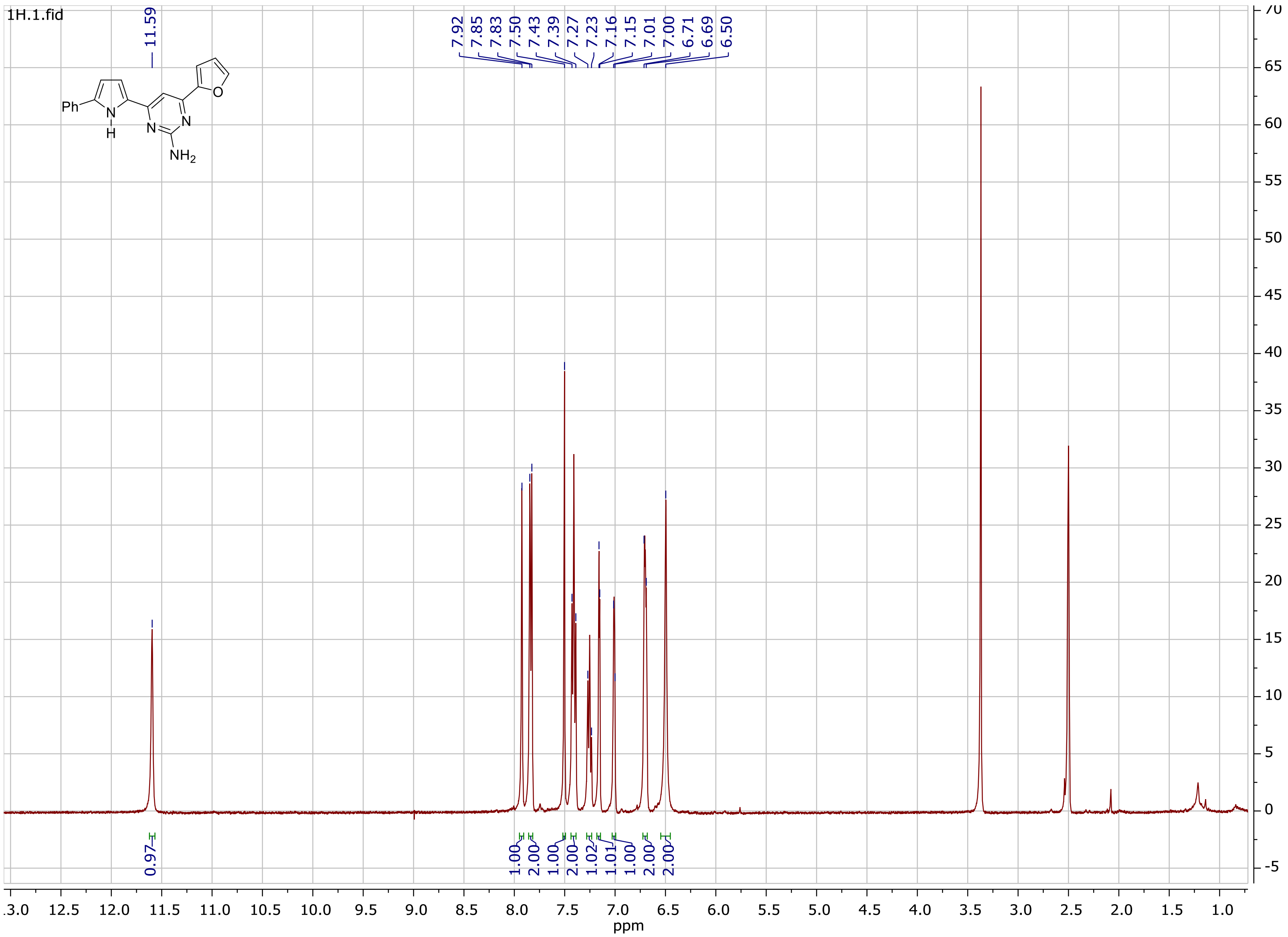

Figure S46: $^{13}$C NMR spectrum (DMSO-d$_6$) 4-(2-furyl)-6-(5-phenyl-1*H*-pyrrol-2-yl)pyrimidin-2-amine **(3m)**

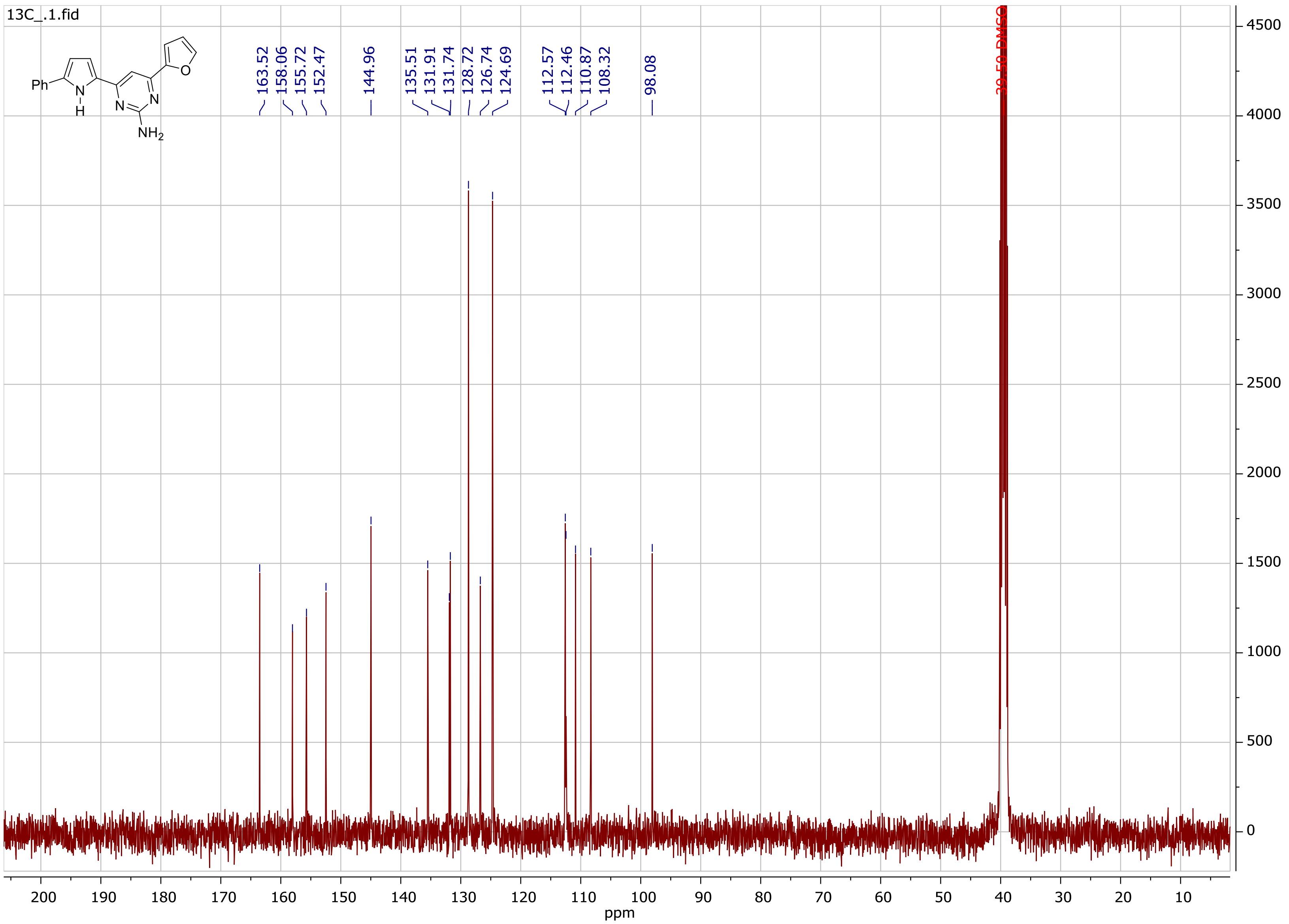

Figure S47: $^1$H NMR spectrum (CDCl$_3$) 4-[5-(4-fluorophenyl)-1*H*-pyrrol-2-yl]-6-phenylpyrimidin-2-amine **(3n)**

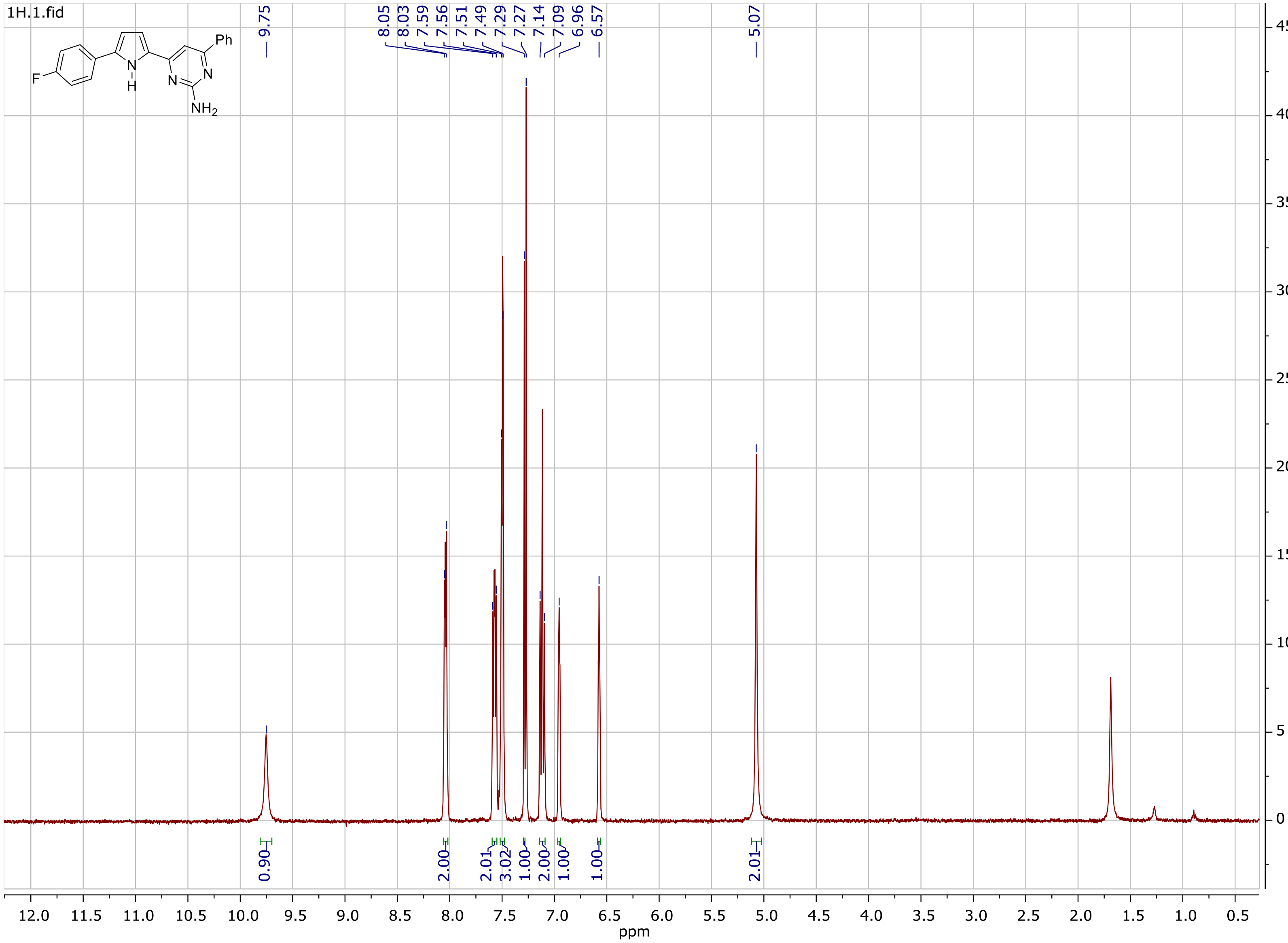

Figure S48: $^{13}$C NMR spectrum (DMSO-d$_6$) 4-[5-(4-fluorophenyl)-1*H*-pyrrol-2-yl]-6-phenylpyrimidin-2-amine **(3n)**

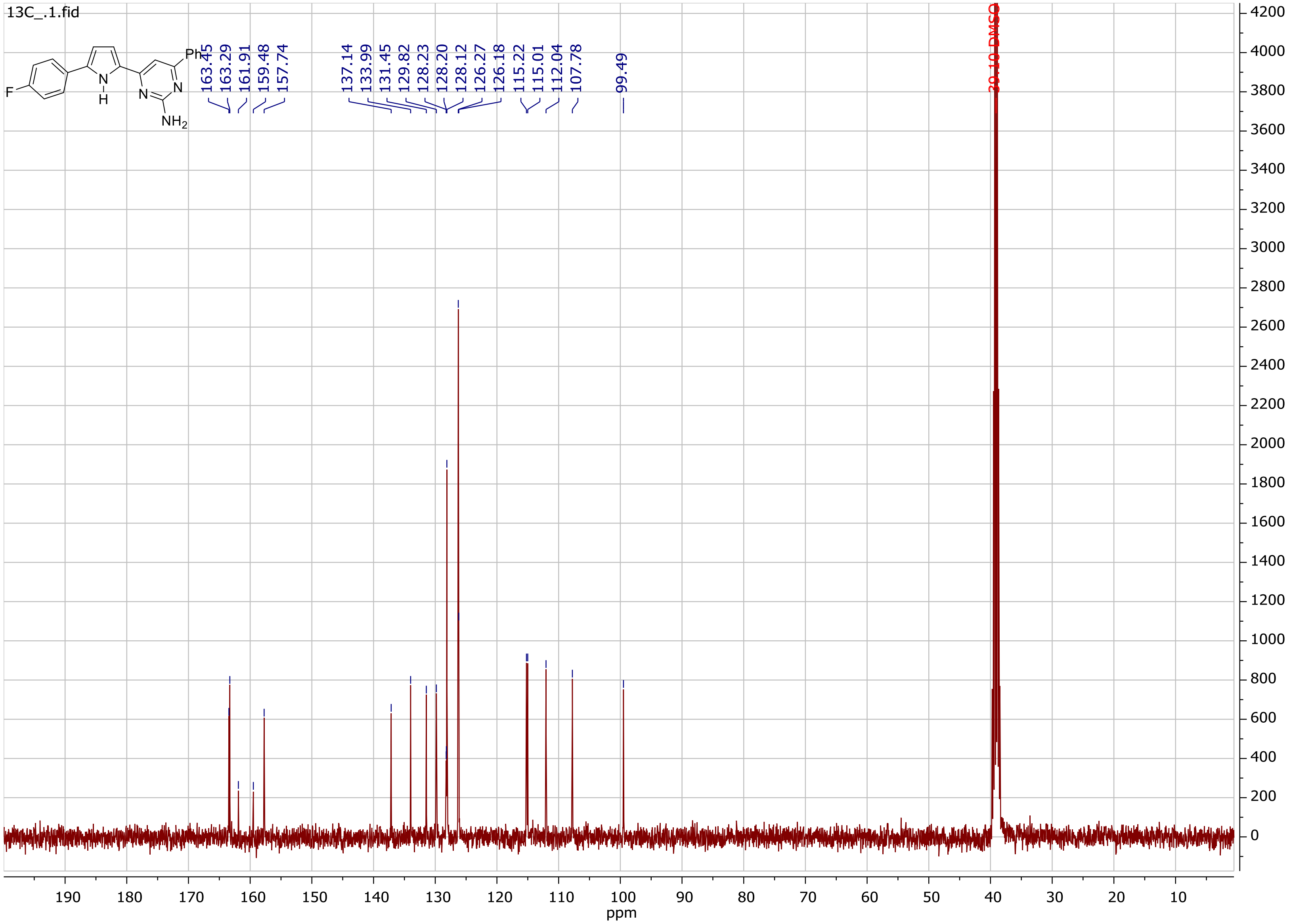

Figure S49: $^{1}$H NMR spectrum ($CDCl_3$) 4-[5-(4-chlorophenyl)-1*H*-pyrrol-2-yl]-6-phenylpyrimidin-2-amine **(3o)**

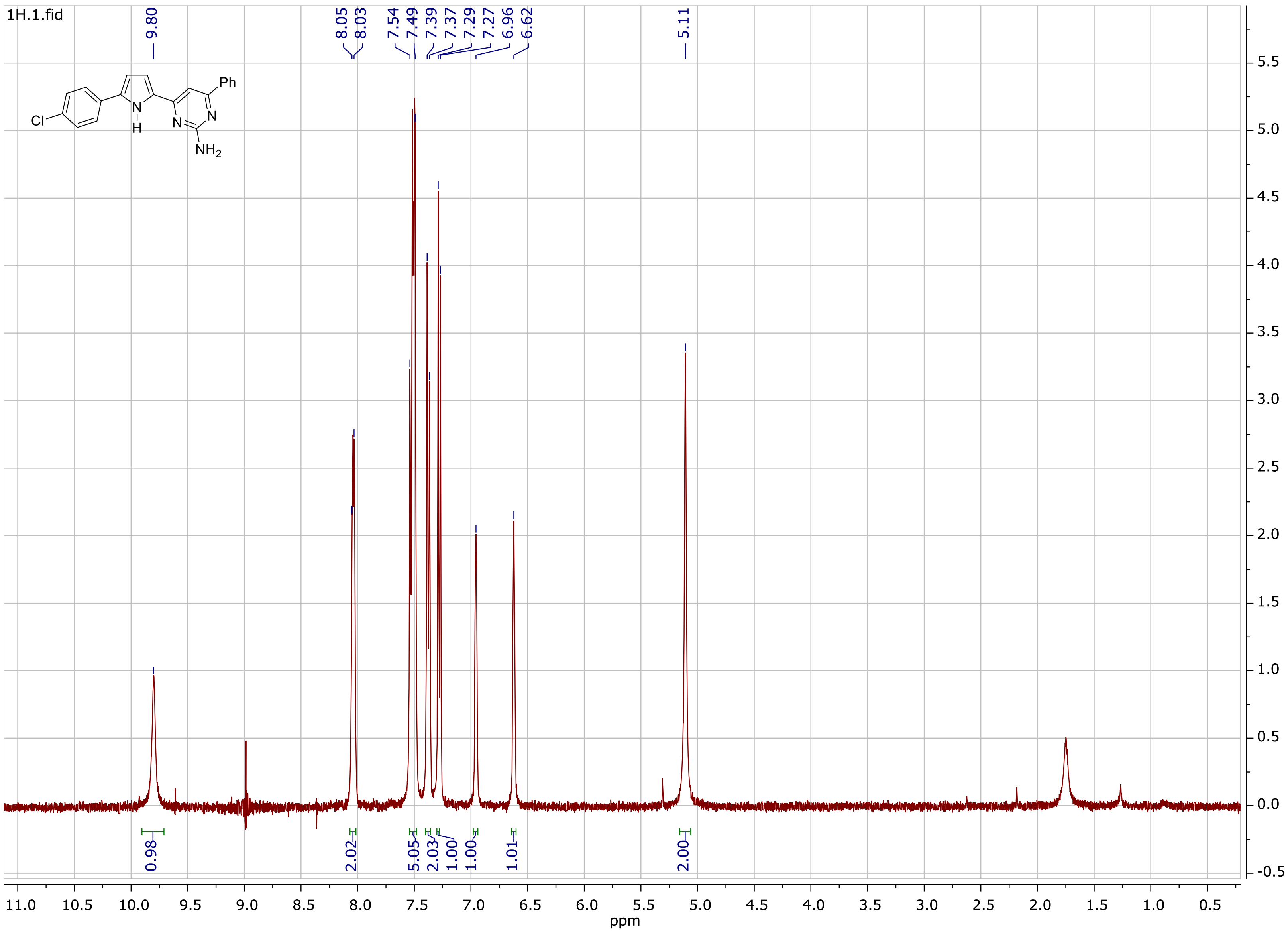

Figure S50: $^{13}$C NMR spectrum (DMSO-$d_6$) 4-[5-(4-chlorophenyl)-1*H*-pyrrol-2-yl]-6-phenylpyrimidin-2-amine **(3o)**

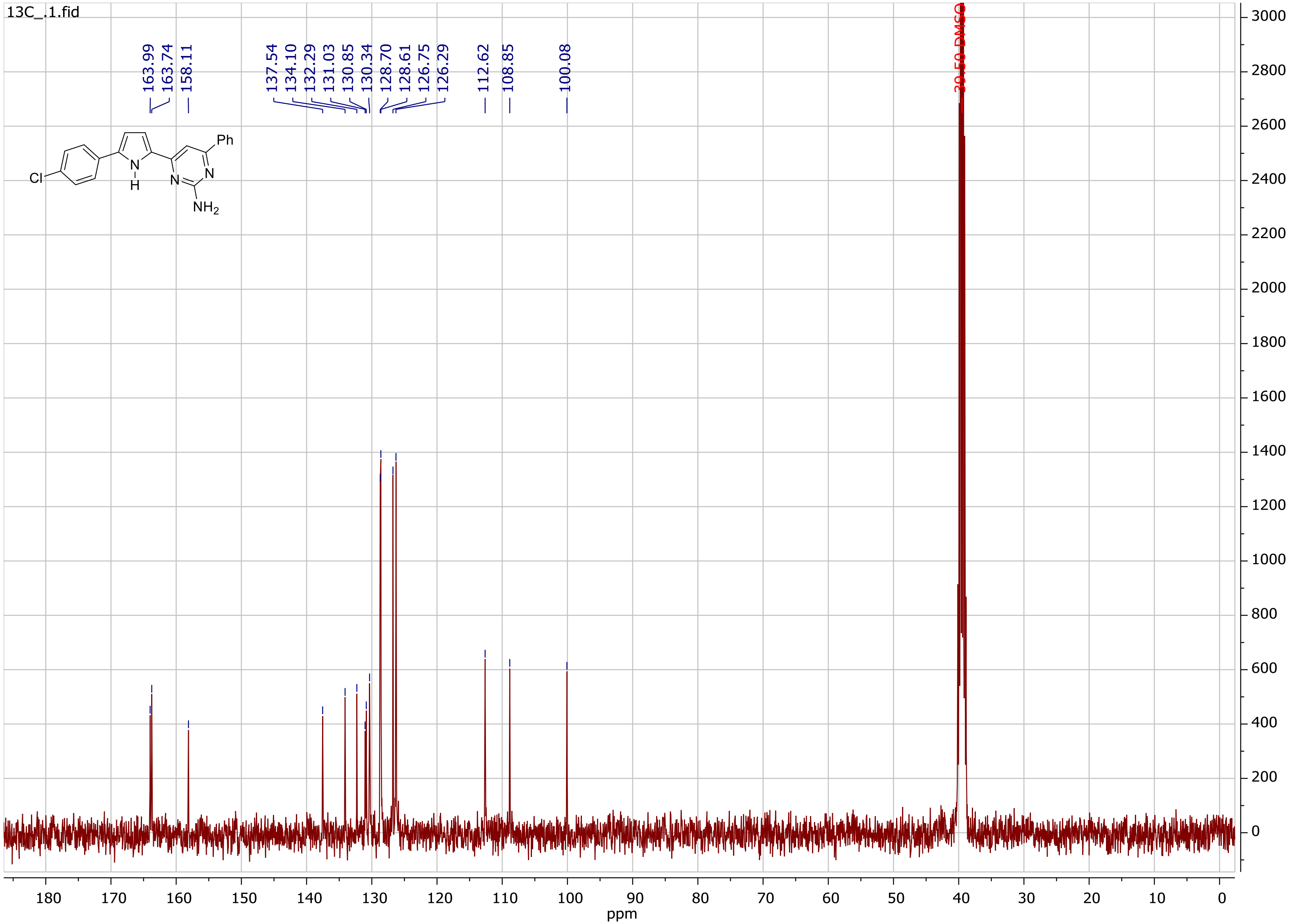

Figure S51: $^{1}$H NMR spectrum ($CDCl_3$) 4-[5-(4-methoxyphenyl)-1*H*-pyrrol-2-yl]-6-phenylpyrimidin-2-amine **(3p)**

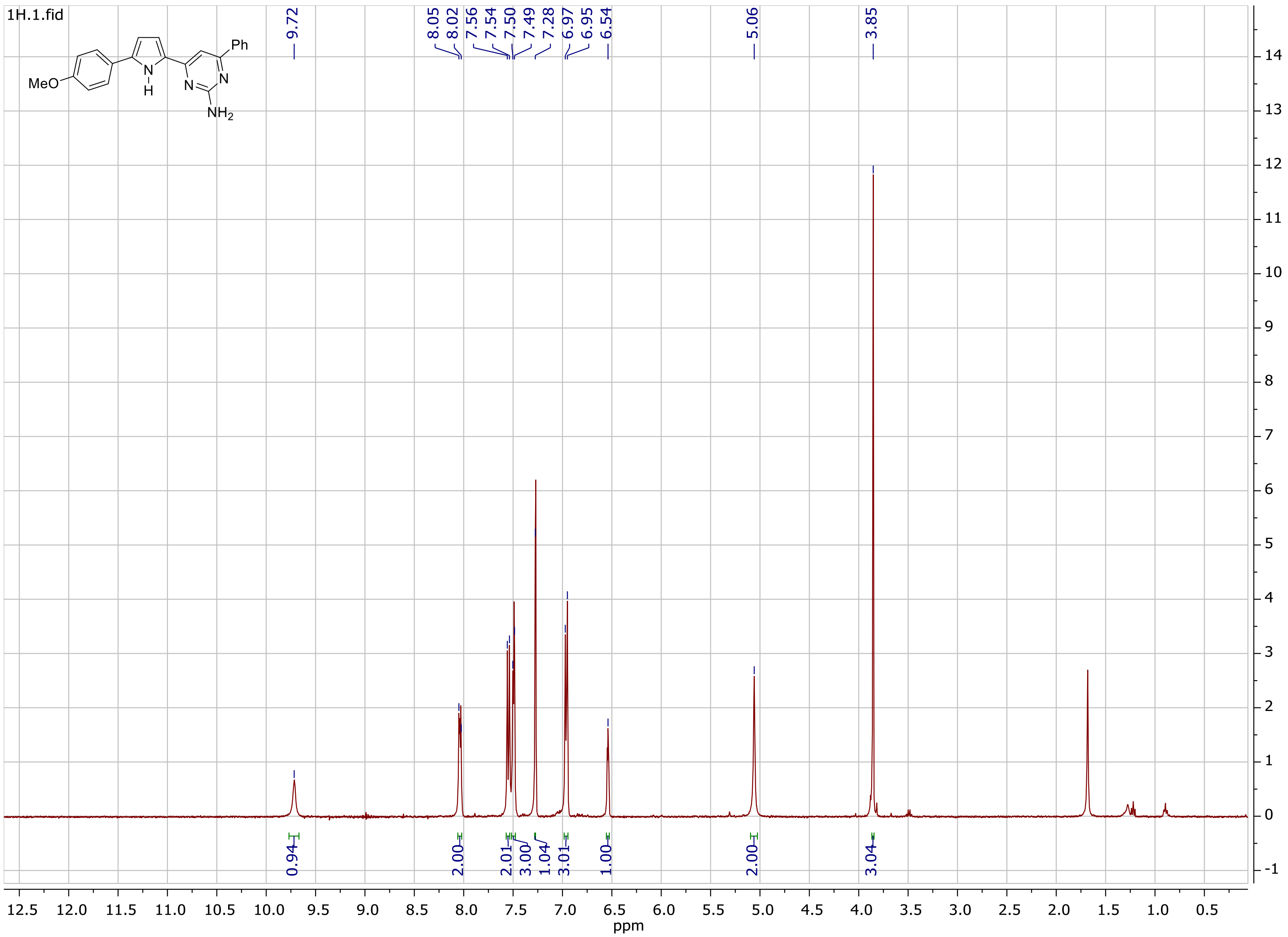

Figure S52: $^{13}$C NMR spectrum (DMSO-$d_6$) 4-[5-(4-methoxyphenyl)-1*H*-pyrrol-2-yl]-6-phenylpyrimidin-2-amine **(3p)**

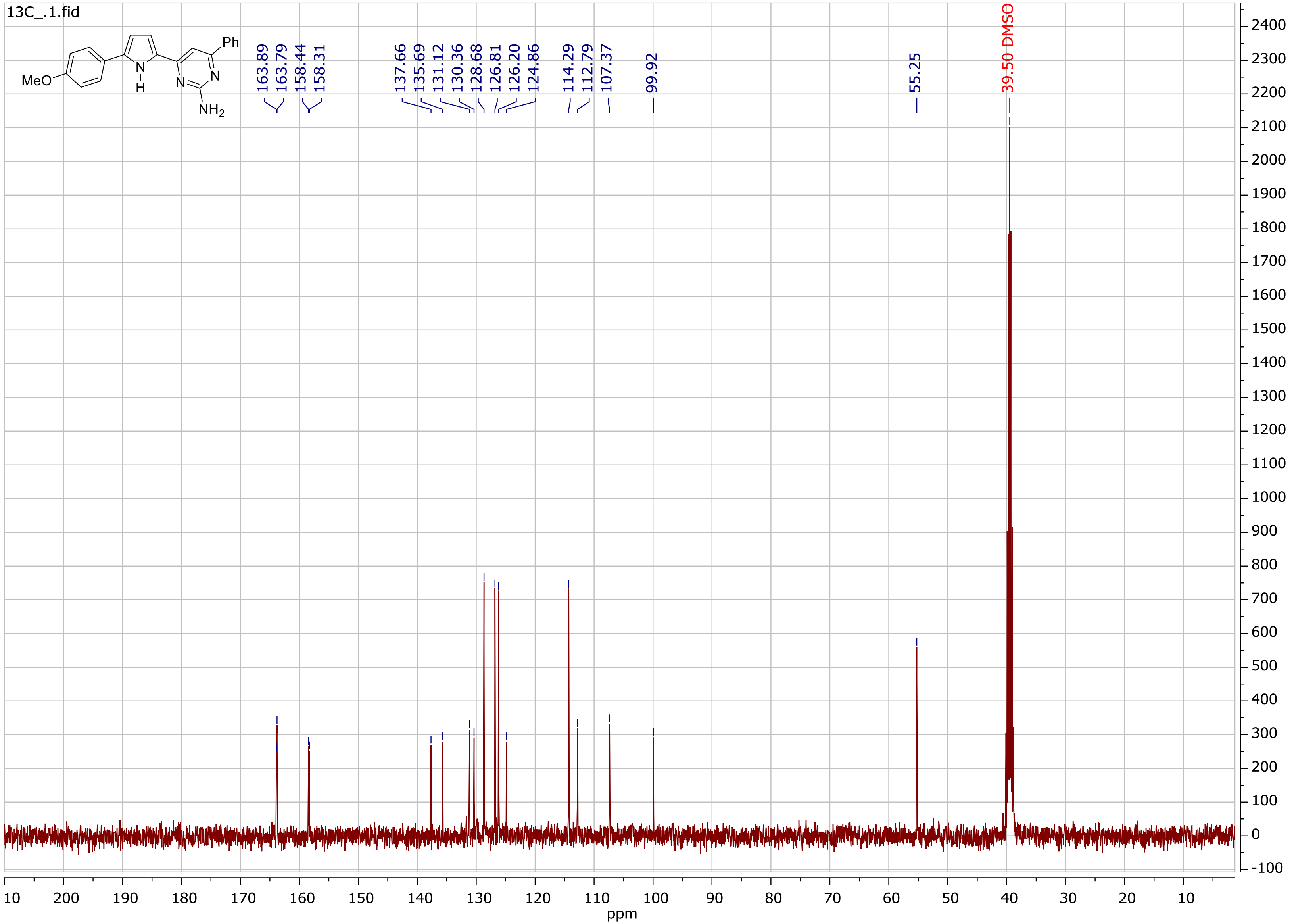

Figure S53: $^{1}$H NMR spectrum ($CDCl_3$) 4-phenyl-6-(1-methyl-5-phenyl-1*H*-pyrrol-2-yl)pyrimidin-2-amine **(3q)**

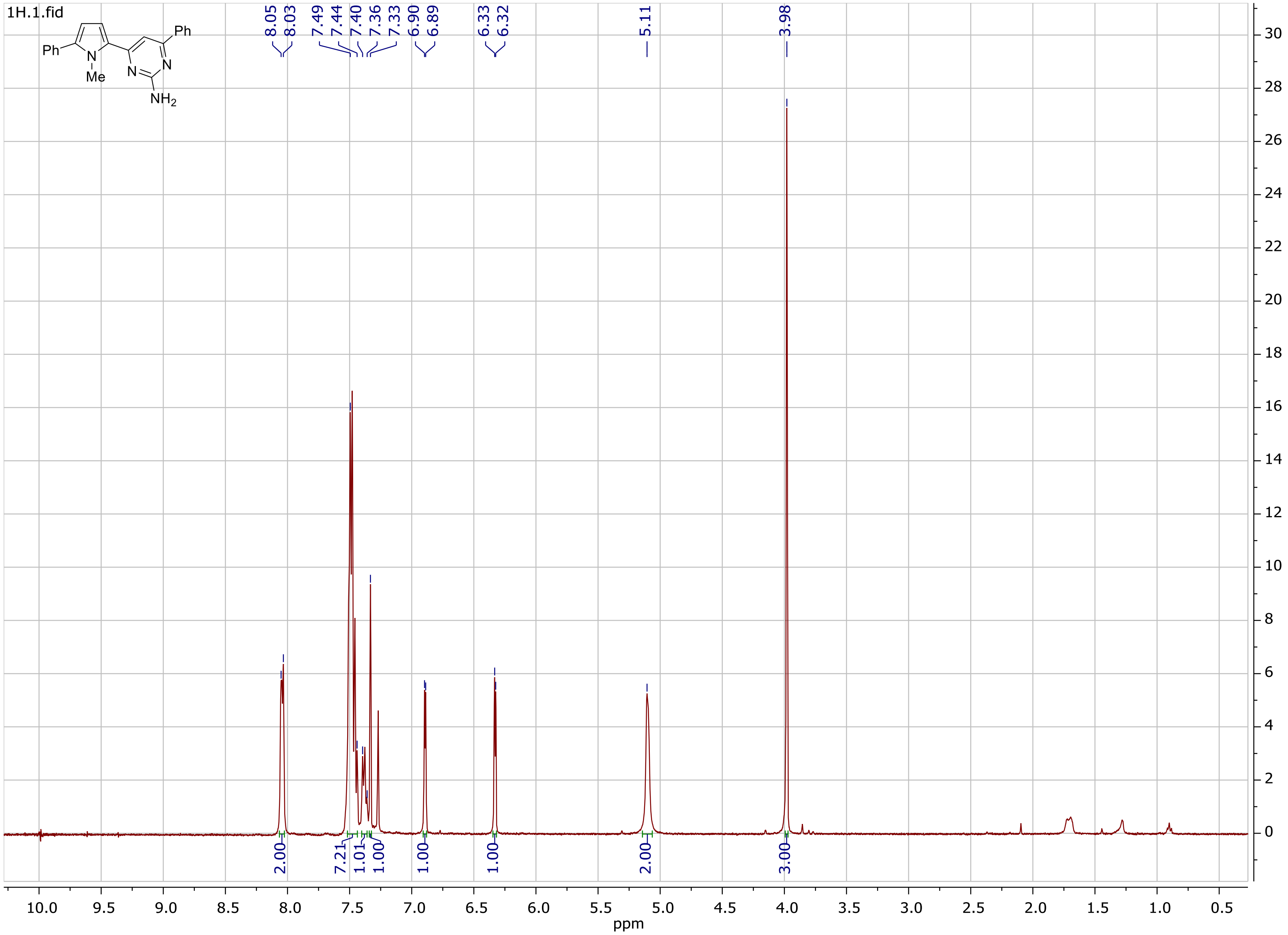

Figure S54: $^{13}$C NMR spectrum ($CDCl_3$) 4-phenyl-6-(1-methyl-5-phenyl-1*H*-pyrrol-2-yl)pyrimidin-2-amine **(3q)**

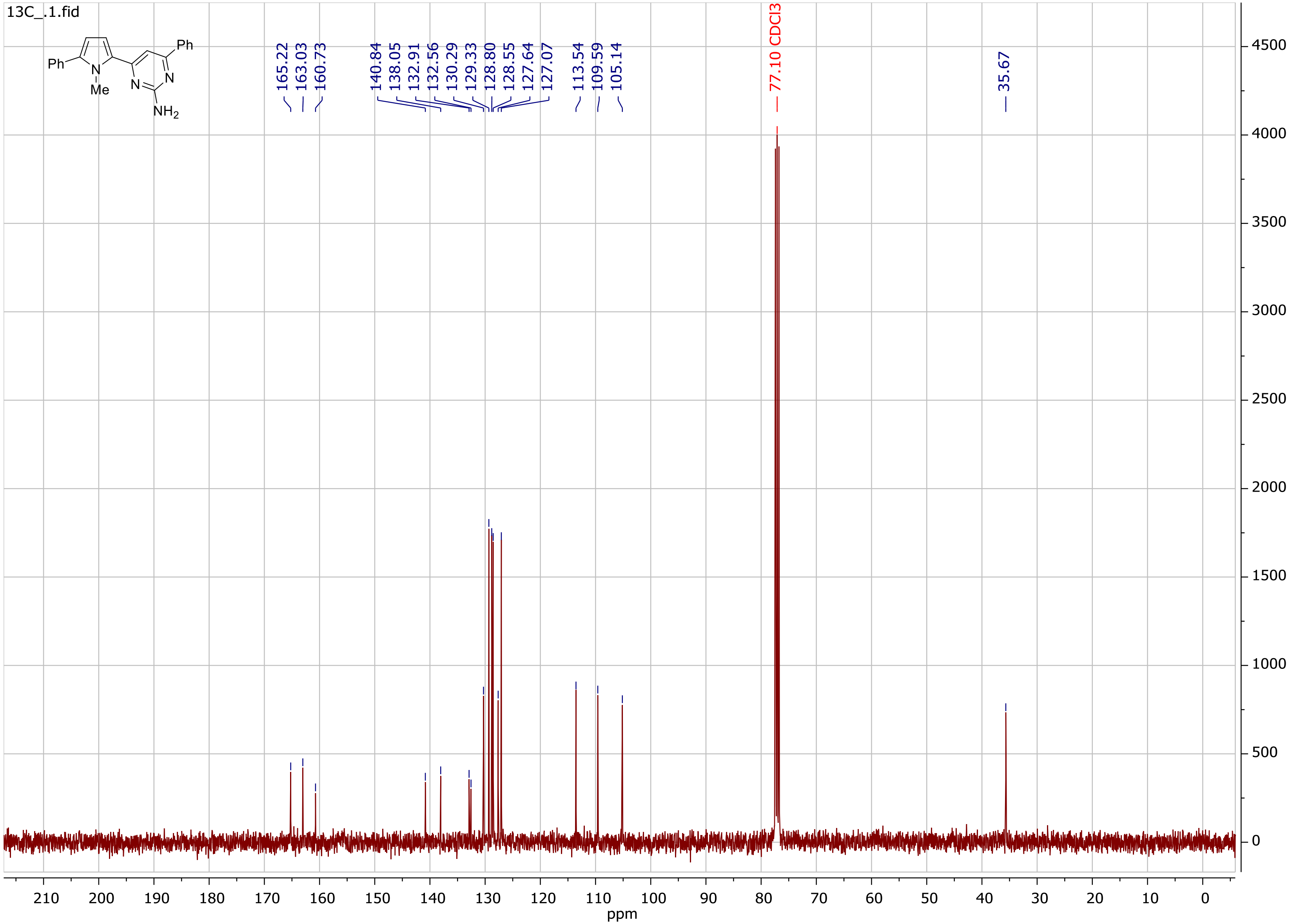

Figure S55: $^{1}$H NMR spectrum ($CDCl_3$) 4-phenyl-6-(5-phenyl-1-vinyl-1*H*-pyrrol-2-yl)pyrimidin-2-amine **(3r)**

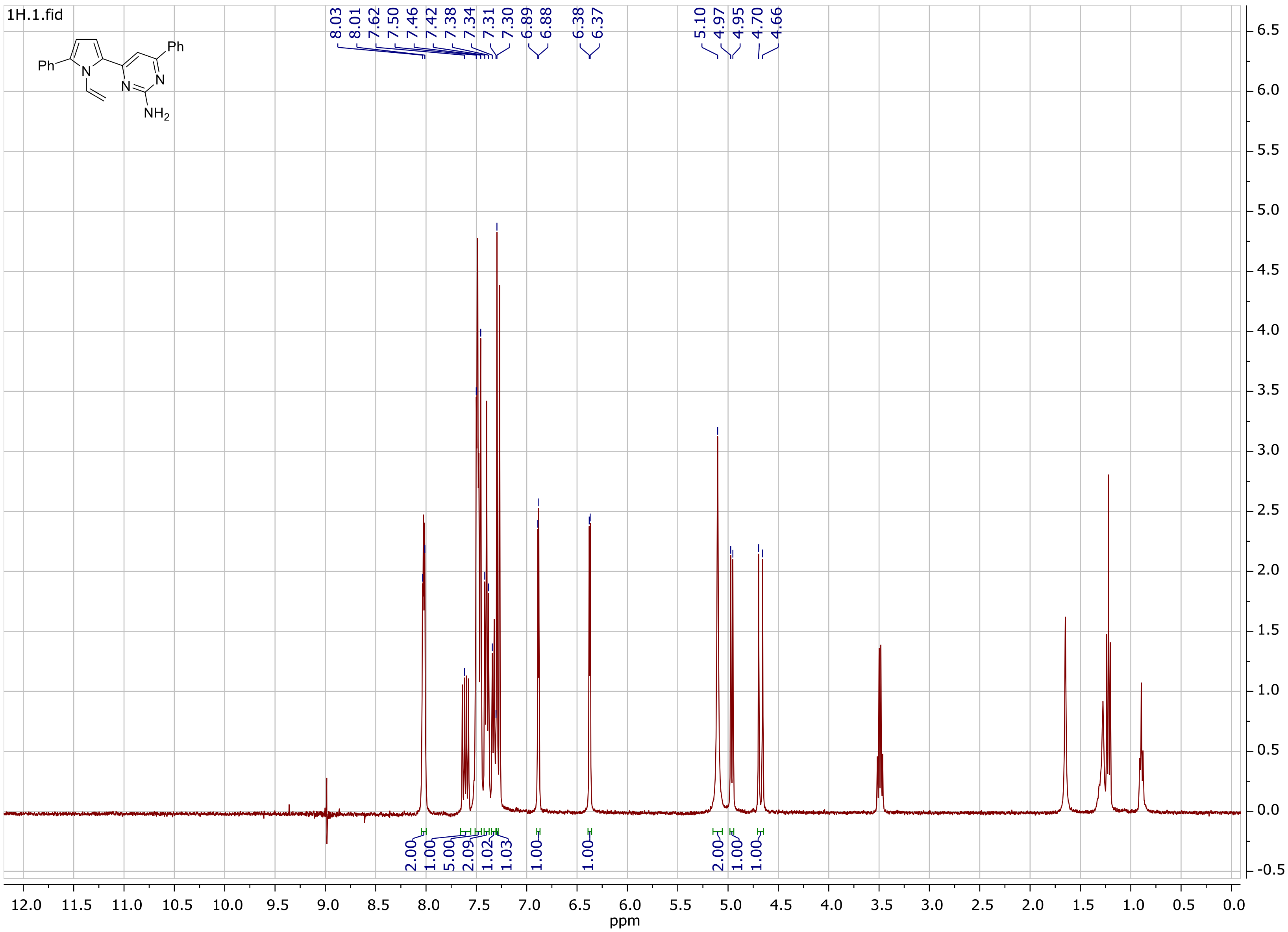

Figure S56: $^{13}$C NMR spectrum ($CDCl_3$) 4-phenyl-6-(5-phenyl-1-vinyl-1*H*-pyrrol-2-yl)pyrimidin-2-amine **(3r)**

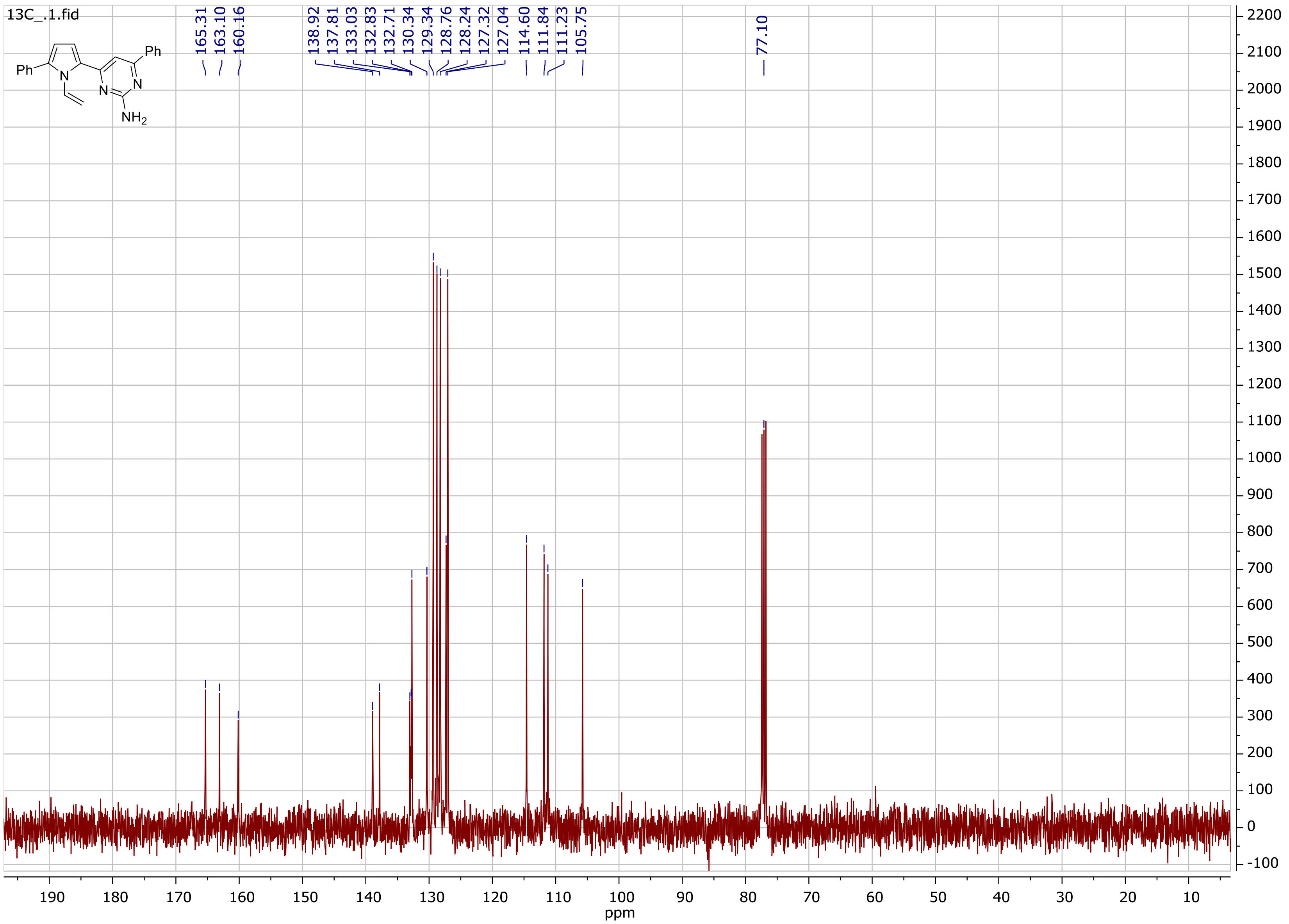

Figure S57: $^{1}$H NMR spectrum ($CDCl_3$) 4-(4,5-diphenyl-1-vinyl-1*H*-pyrrol-2-yl)-6-phenylpyrimidin-2-amine **(3s)**

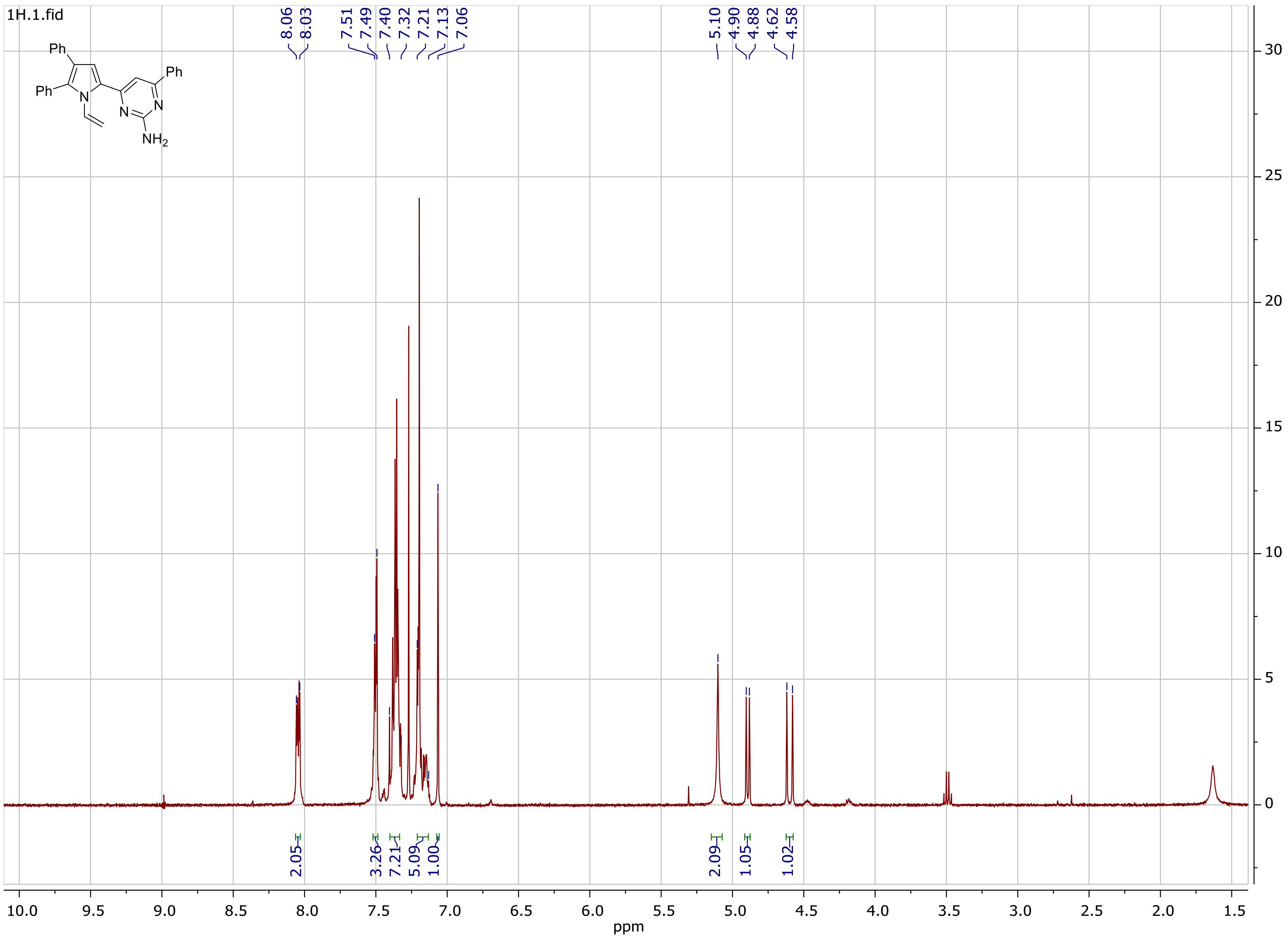

Figure S58: $^{13}C$ NMR spectrum ($CDCl_3$) 4-(4,5-diphenyl-1-vinyl-1*H*-pyrrol-2-yl)-6-phenylpyrimidin-2-amine **(3s)**

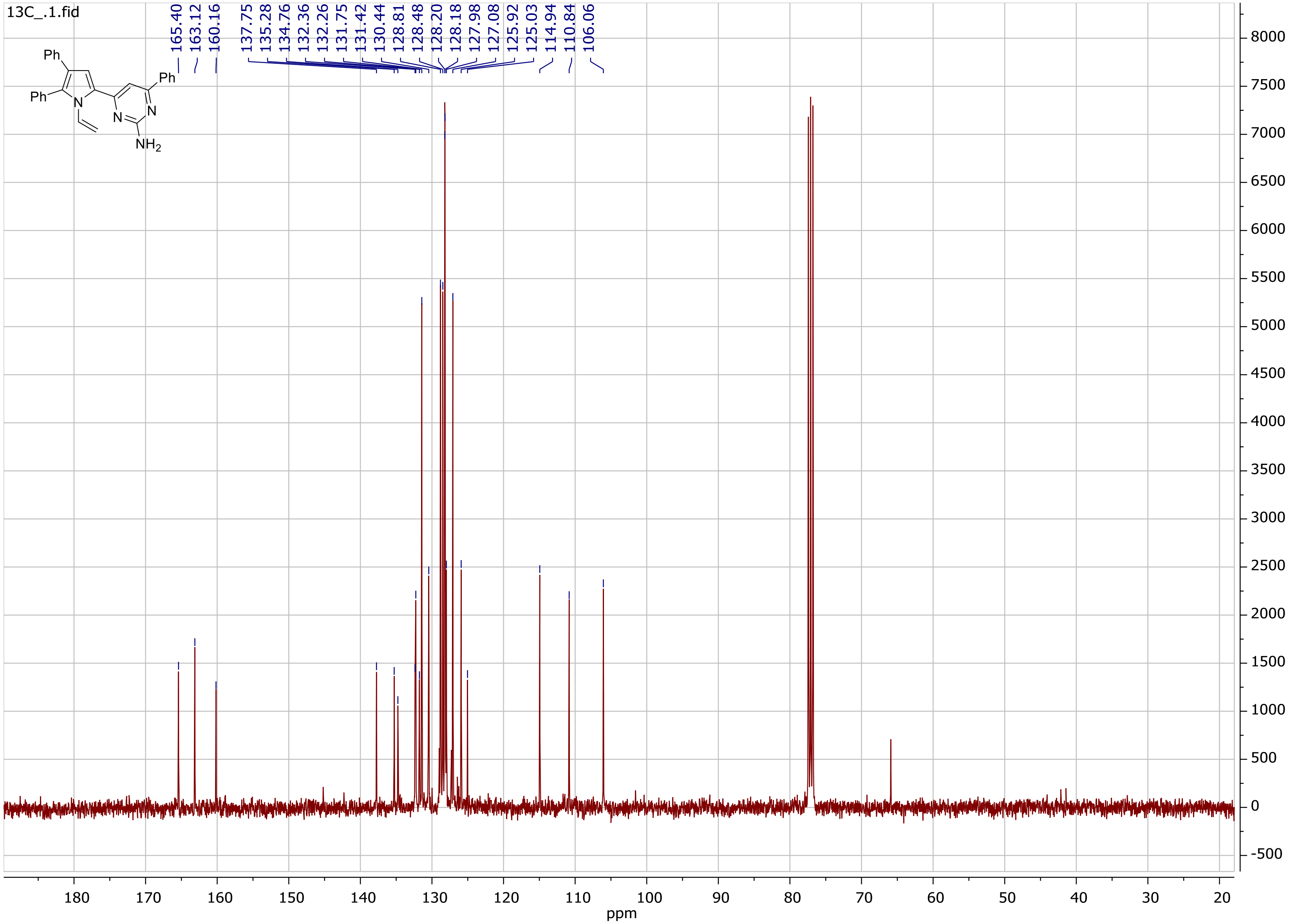

Figure S59: $^{1}$H NMR spectrum ($CDCl_3$) 4-[5-(4-chlorophenyl)-1-vinyl-1*H*-pyrrol-2-yl]-6-phenylpyrimidin-2-amine **(3t)**

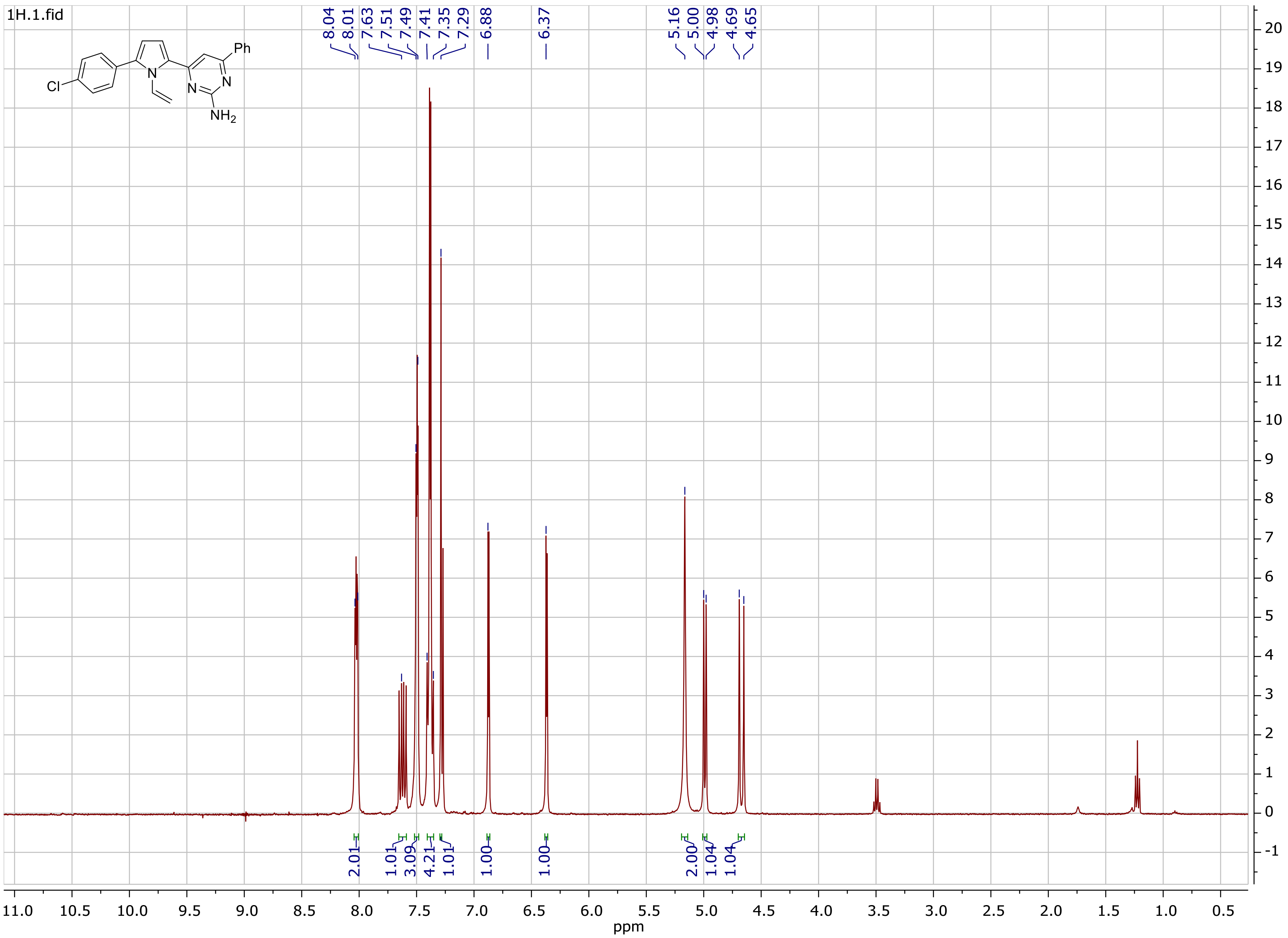

Figure S60: $^{13}C$ NMR spectrum ($CDCl_3$) 4-[5-(4-chlorophenyl)-1-vinyl-1*H*-pyrrol-2-yl]-6-phenylpyrimidin-2-amine **(3t)**

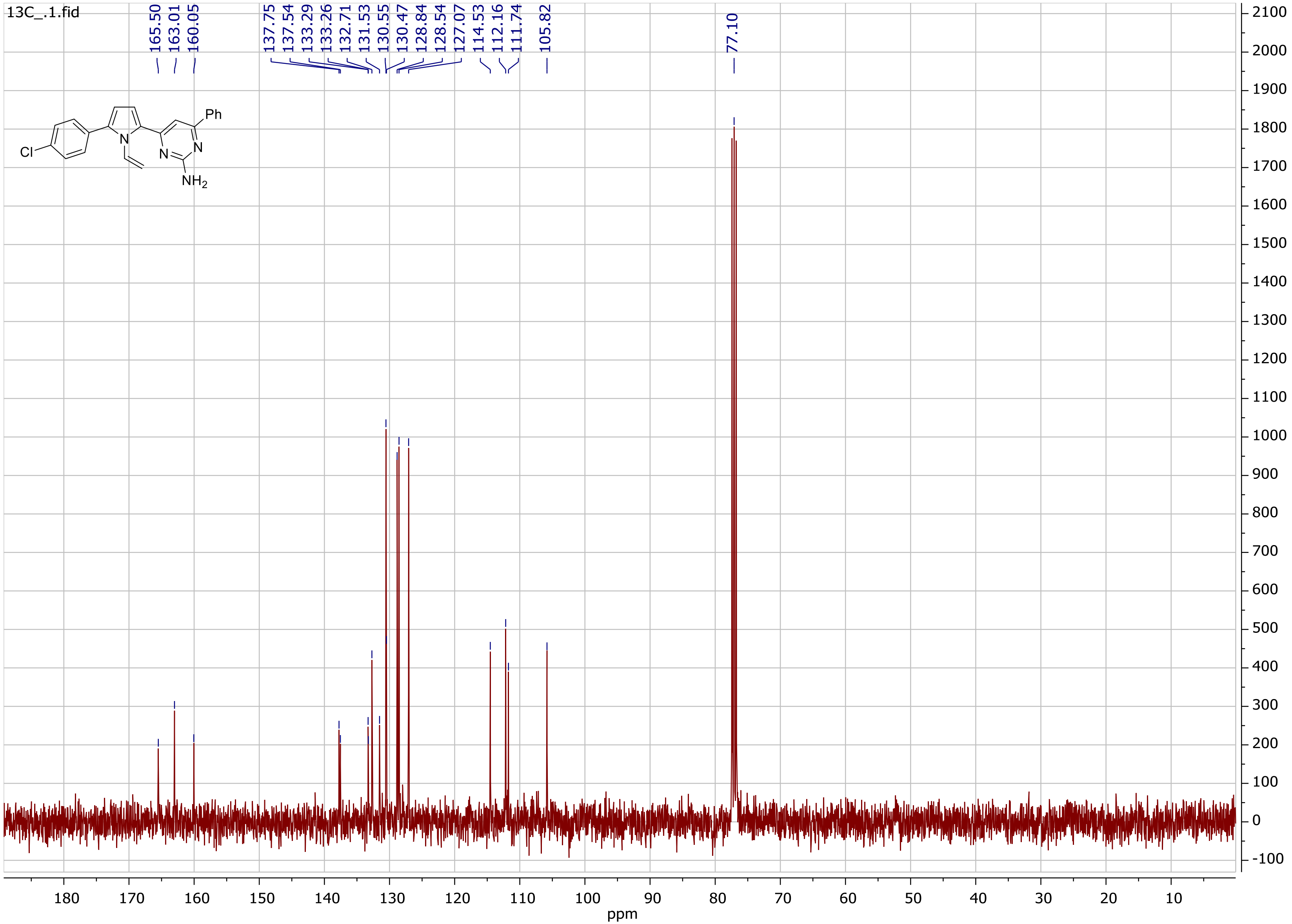

Figure S61: $^{1}$H NMR spectrum ($CDCl_3$) 4-[5-(2-fluorophenyl)-1-vinyl-1*H*-pyrrol-2-yl]-6-(2-furyl)pyrimidin-2-amine **(3u)**

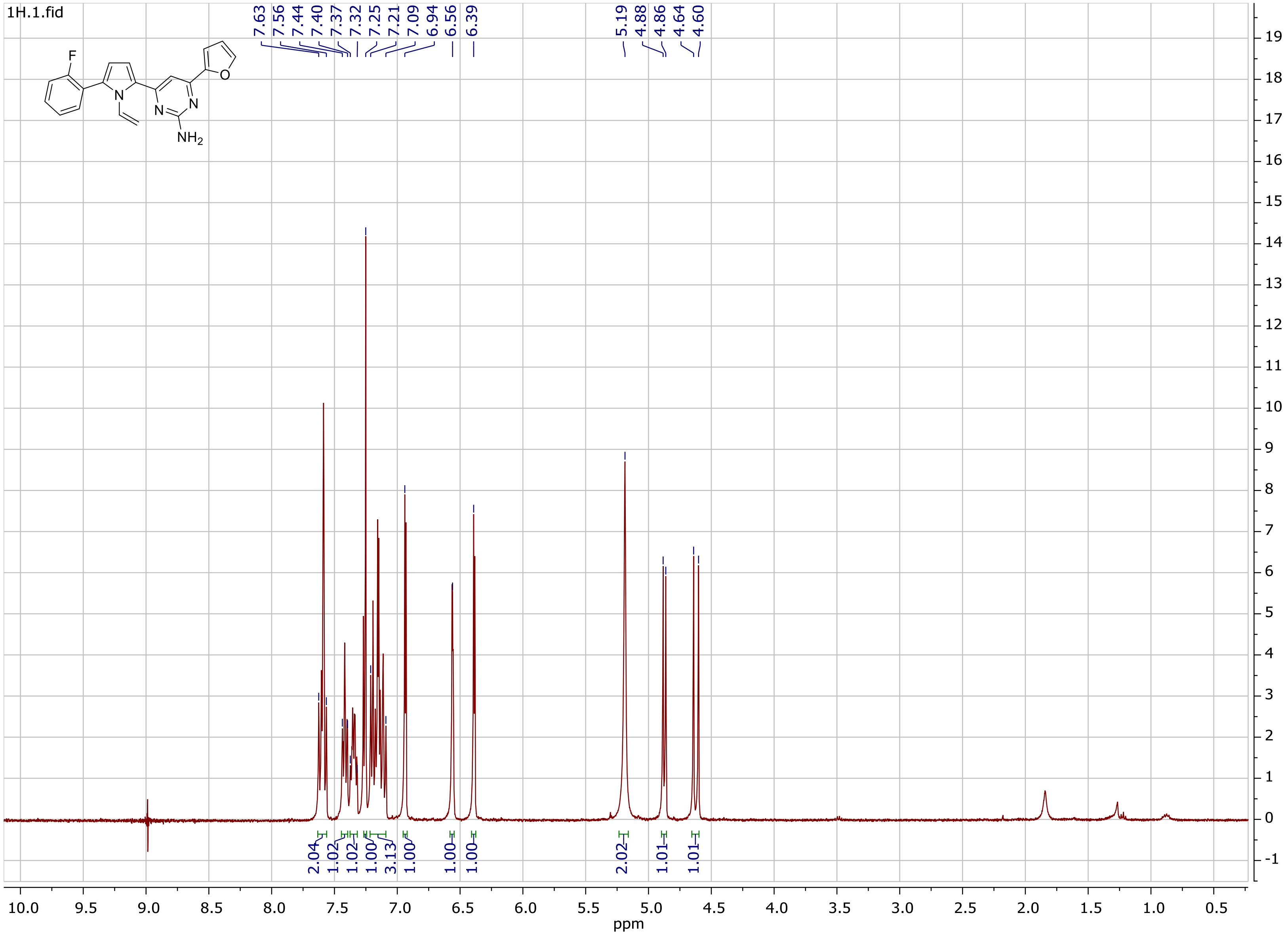

Figure S62: $^{13}$C NMR spectrum ($CDCl_3$) 4-[5-(2-fluorophenyl)-1-vinyl-1*H*-pyrrol-2-yl]-6-(2-furyl)pyrimidin-2-amine **(3u)**

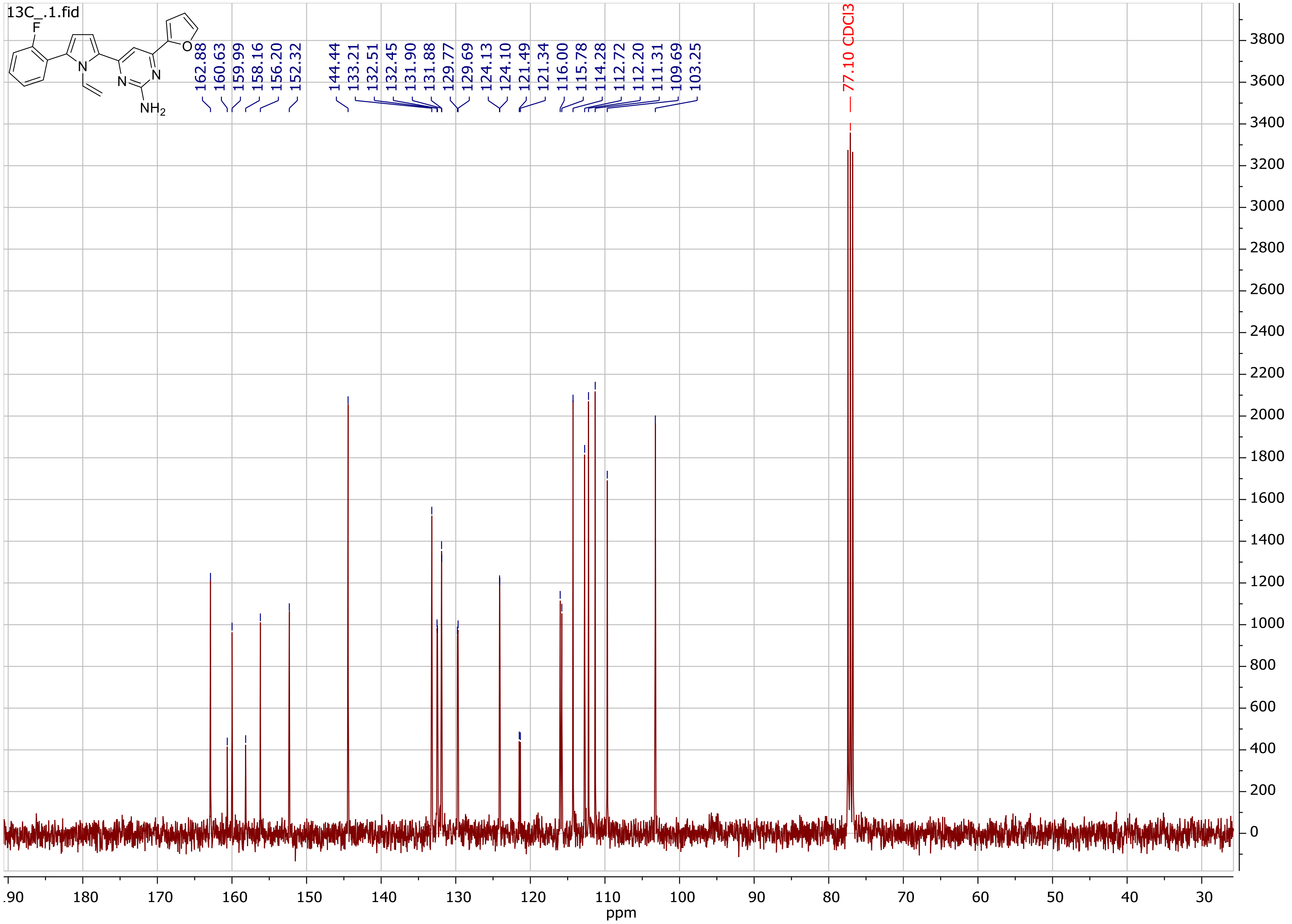

Figure S63: $^{1}$H NMR spectrum ($CDCl_3$) 4-[5-(2-fluorophenyl)-1-vinyl-1H-pyrrol-2-yl]-6-(2-thienyl)pyrimidin-2-amine **(3v)**

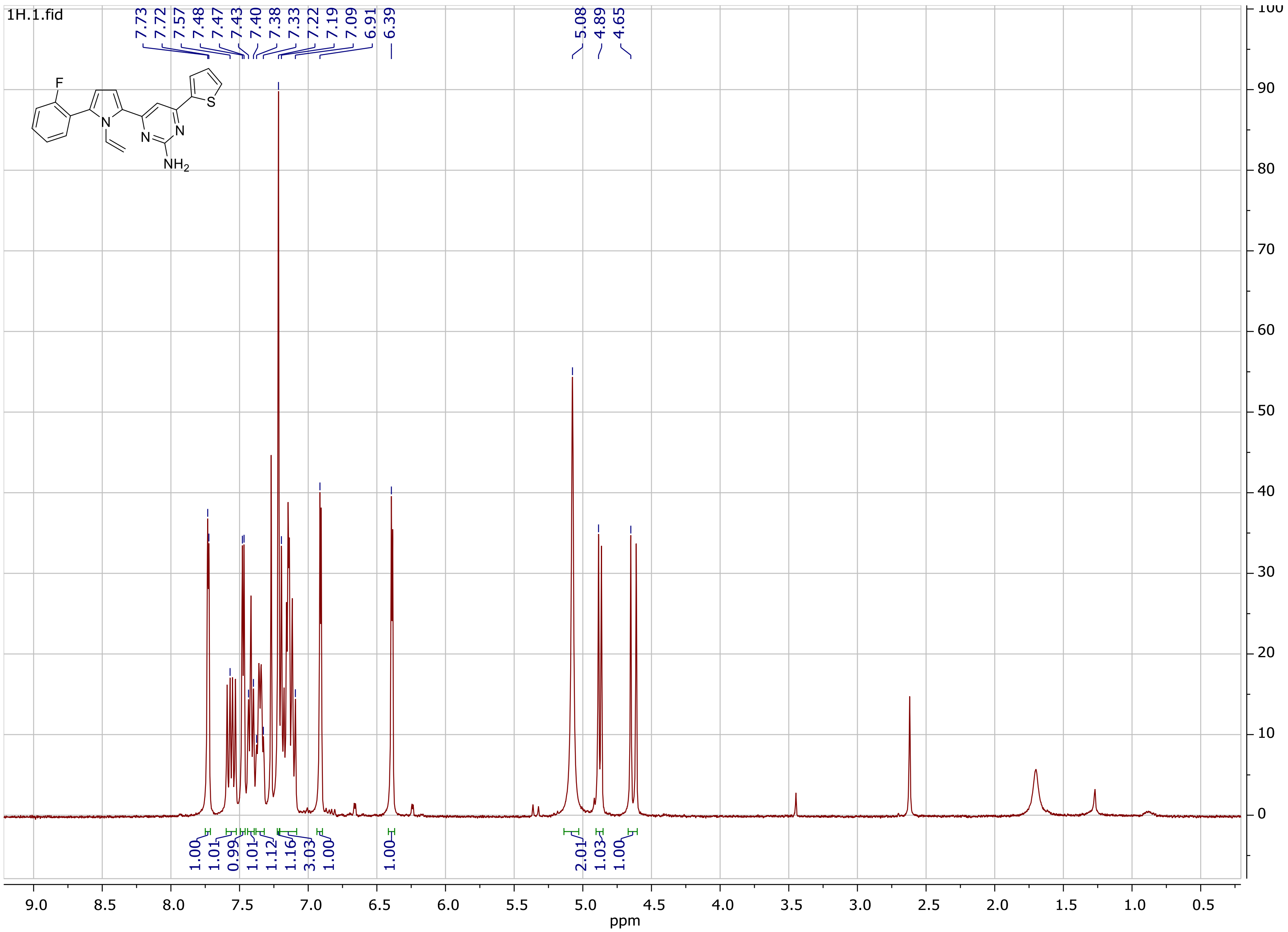

Figure S64: $^{1}$H NMR spectrum ($CDCl_3$) 4-[5-(2-fluorophenyl)-1-vinyl-1H-pyrrol-2-yl]-6-(2-thienyl)pyrimidin-2-amine **(3v)**

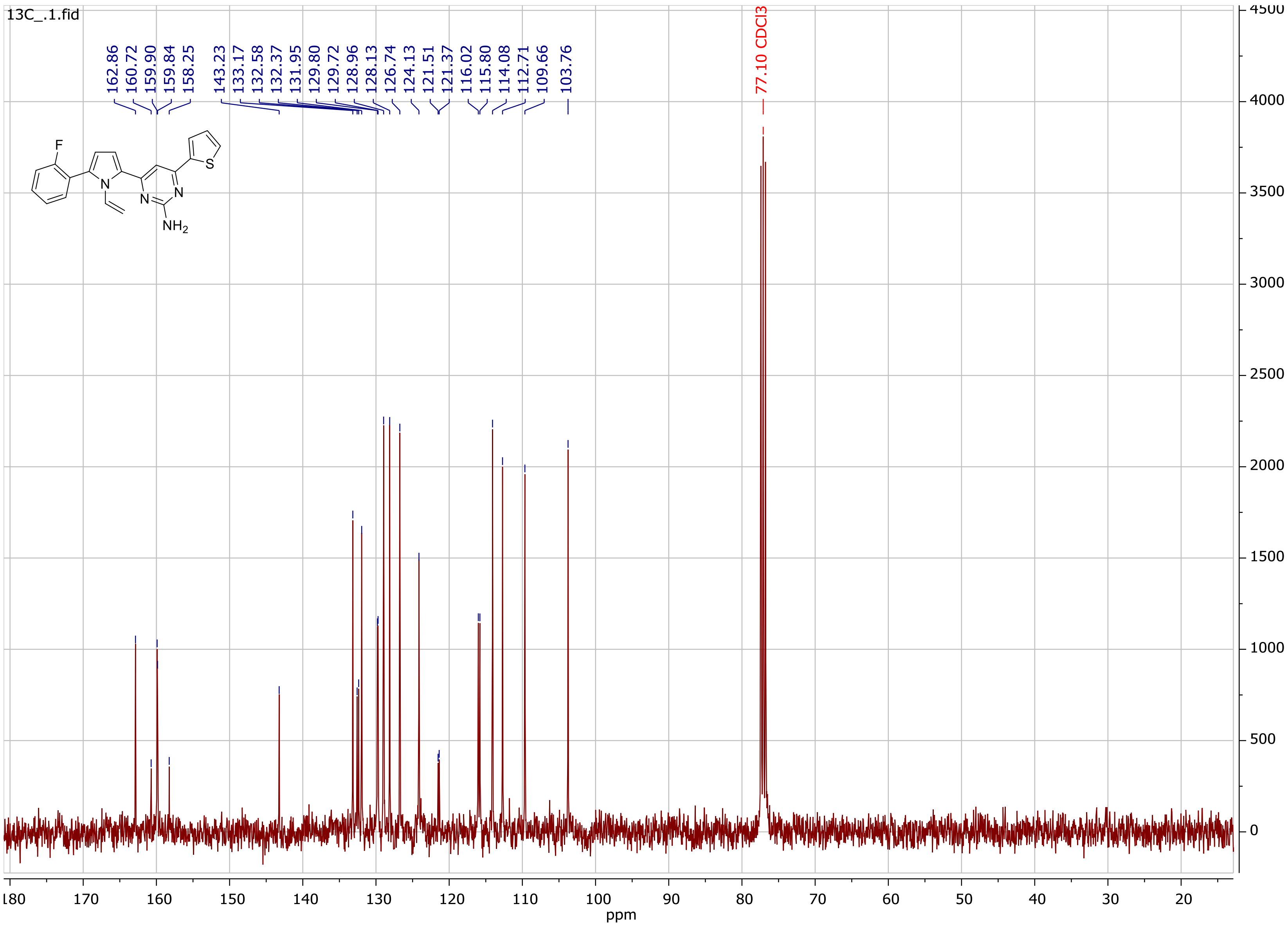

Figure S65: $^{1}$H NMR spectrum ($CDCl_3$) 3-[2-(2-amino-6-phenylpyrimidin-4-yl)-1*H*-pyrrol-1-yl]-1-phenyl-3-(1*H*-pyrrol-2-yl)prop-2-en-1-one (**4a**)

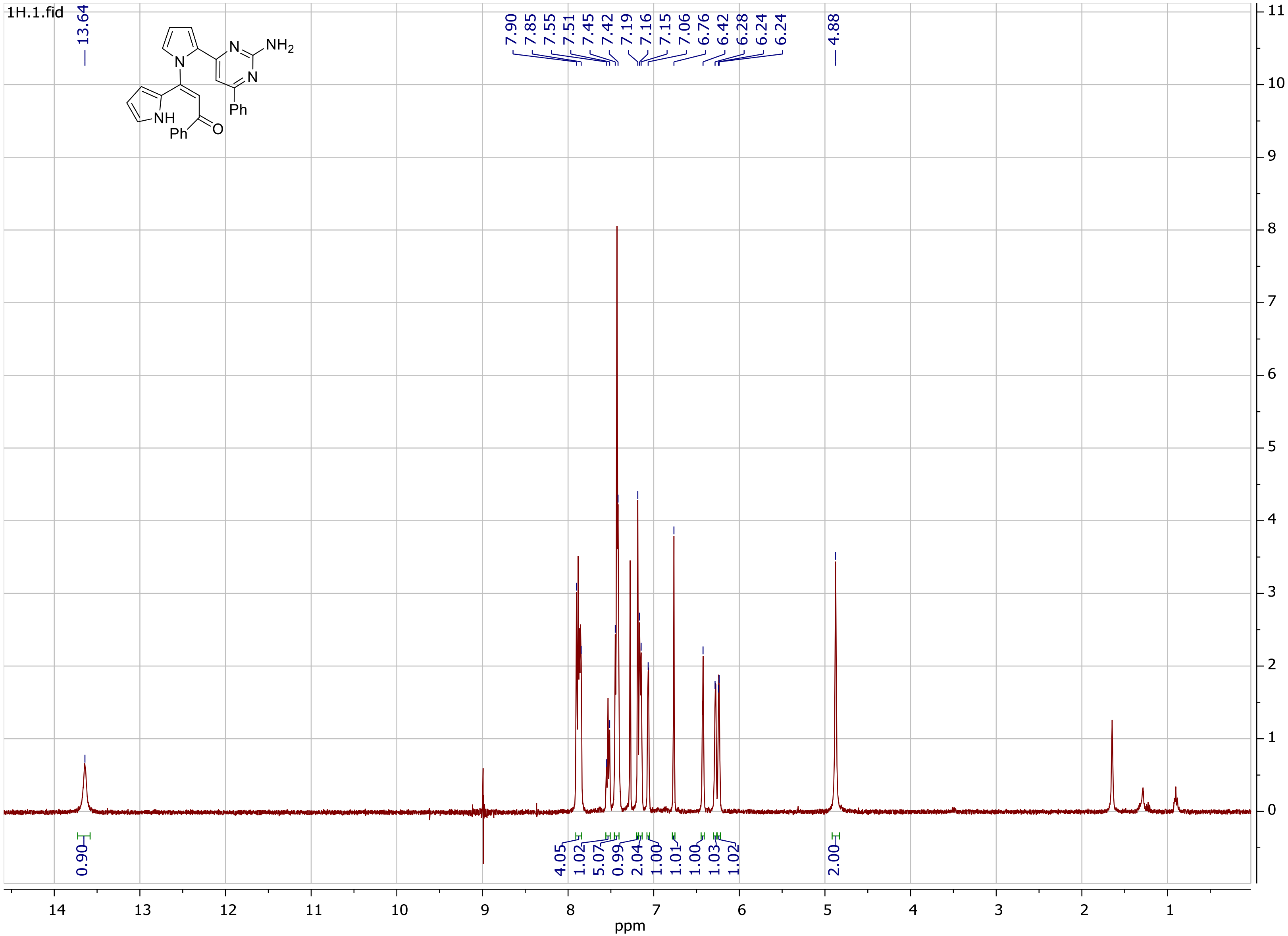

Figure S66: $^{13}$C NMR spectrum ($CDCl_3$) 3-[2-(2-amino-6-phenylpyrimidin-4-yl)-1*H*-pyrrol-1-yl]-1-phenyl-3-(1*H*-pyrrol-2-yl)prop-2-en-1-one (**4a**)

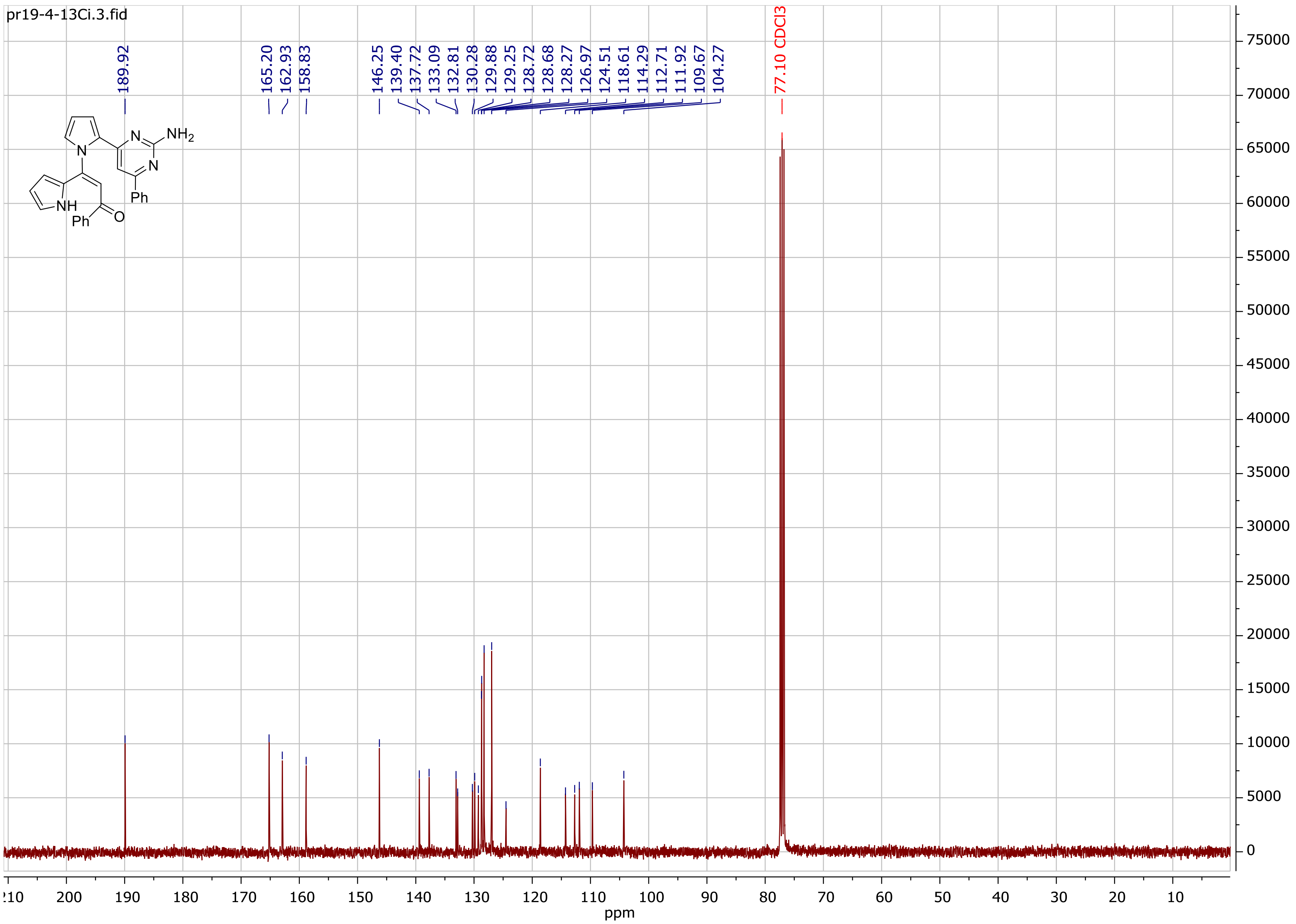

Figure S67: $^1$H NMR spectrum ($CDCl_3$) 3-[2-amino-4-phenyl-6-(1*H*-pyrrol-2-yl)pyrimidin-5-yl]-1-phenyl-3-(1*H*-pyrrol-2-yl)prop-2-en-1-one **(5a)**

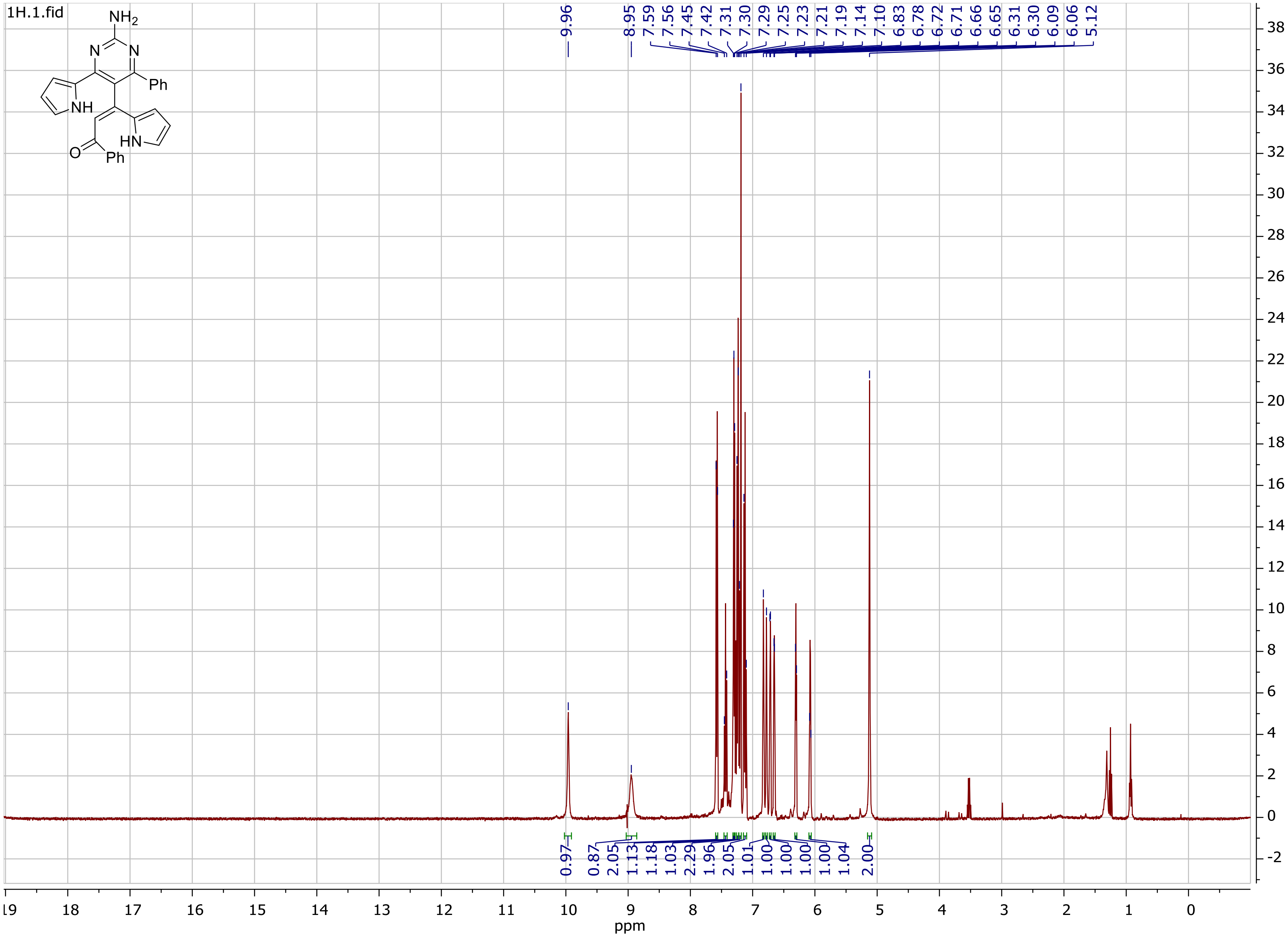

Figure S68: $^{13}$C NMR spectrum (DMSO-d$_6$) 3-[2-amino-4-phenyl-6-(1*H*-pyrrol-2-yl)pyrimidin-5-yl]-1-phenyl-3-(1*H*-pyrrol-2-yl)prop-2-en-1-one **(5a)**

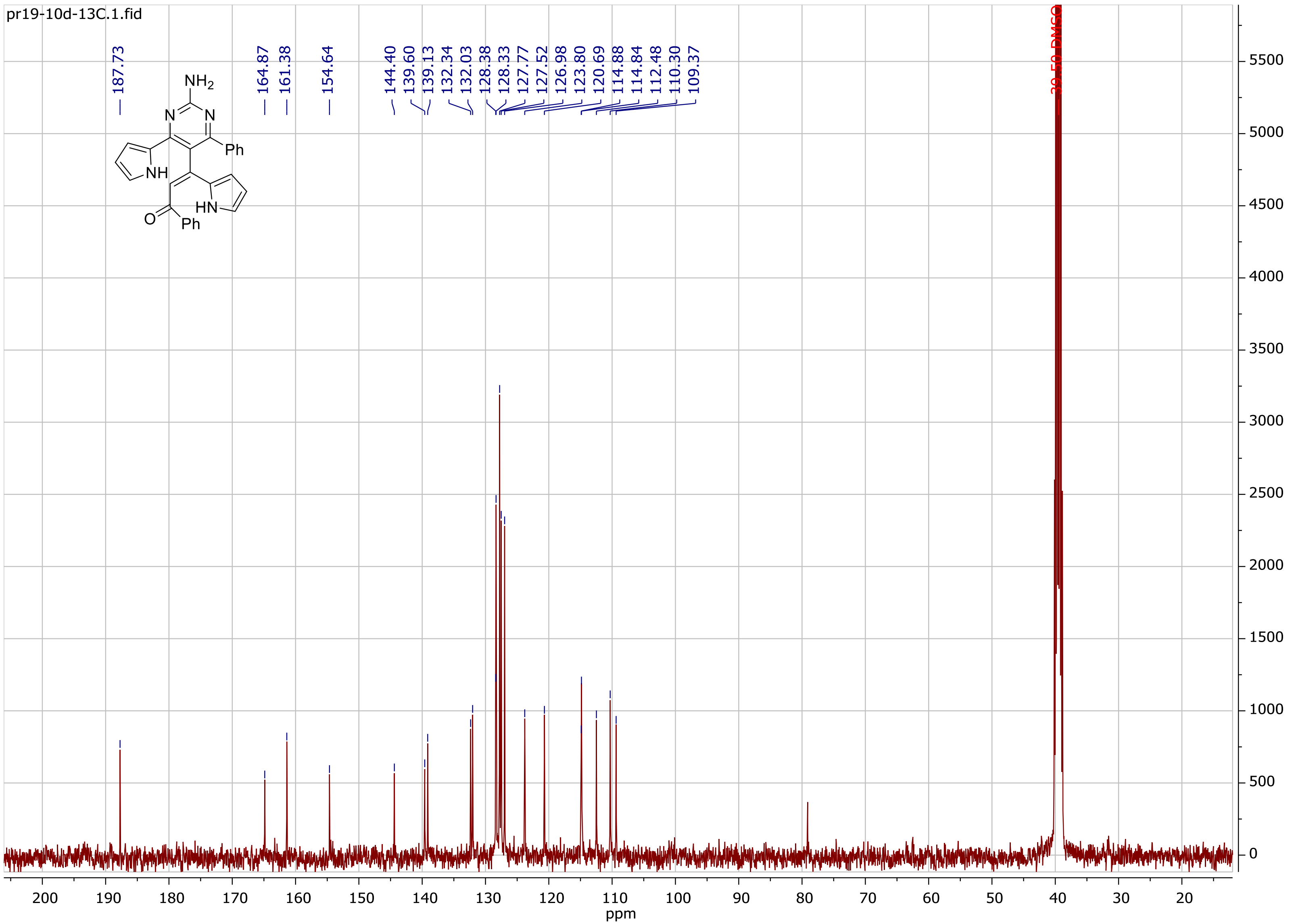

Supplement: Supplementary file 1 [file molecules-26-01692-s001.pdf]
